# Development and validation of a metastasis-related Gene Signature for predicting the Overall Survival in patients with Pancreatic Ductal Adenocarcinoma

**DOI:** 10.7150/jca.47629

**Published:** 2020-08-29

**Authors:** Mengwei Wu, Xiaobin Li, Rui Liu, Hongwei Yuan, Wei Liu, Ziwen Liu

**Affiliations:** Department of General Surgery, Peking Union Medical College Hospital, Chinese Academy of Medical Sciences & Peking Union Medical College, Beijing, China.

**Keywords:** Gene Expression Omnibus, The Cancer Genome Atlas Program, pancreatic ductal adenocarcinoma, overall survival, nomogram

## Abstract

**Background:** Pancreatic ductal adenocarcinoma (PDAC) is a highly fatal, aggressive cancer characterized by invasiveness and metastasis. In this study, we aimed to propose a gene prediction model based on metastasis-related genes (MTGs) to more accurately predict PDAC prognosis.

**Methods:** Differentially expressed MTGs (DE-MTGs) were identified via integrated analysis of gene expression omnibus (GEO) datasets and Human Cancer Metastasis Database (HCMDB). Overall survival (OS) related DE-MTGs were then identified and a prognostic gene signature was established using Lasso-Cox regression with TCGA-PAAD datasets. Tumor immunity was analyzed using ESTIMATE and CIBERSORT algorithms. Finally, a nomogram predicting 1-year, 2-year, and 3-year OS of PDAC patients was established based on the prognostic gene signature and relevant clinical parameters using a stepwise Cox regression model.

**Results:** A total of 36 DE-MTGs related to OS were identified in PDAC. Consequently, an MTG-based gene signature comprising of *RACGAP1*, *RARRES3*, *TPX2*, *MMP28*, *GPR87*, *KIF14*, and *TSPAN7* was established to predict the OS of PDAC. The MTG-based gene signature was able to distinguish high-risk patients with significantly poorer prognosis and accurately predict OS of PDAC in both the training and external validation datasets. Cox regression analysis indicated that the MTG-based gene signature was an independent prognostic factor in PDAC. The gene set enrichment analysis (GSEA) showed that molecular alterations in the high-risk group were associated with multiple oncological pathways. Moreover, analysis of tumor immunity revealed significantly higher levels of follicular helper T cells and M0 macrophage infiltration, and lower levels of infiltrating naïve B cells, CD8 T cells, monocytes, and resting dendritic cells in the high-risk group. Immune cell infiltration levels were significantly associated with the expression of the seven DE-MTGs. Finally, a nomogram was established by incorporating the prognostic gene signature and clinical parameters, which was superior to the AJCC staging system in predicting the OS of PDAC patients.

**Conclusions:** The DE-MTGs we identified were closely associated with the progress and prognosis of PDAC and are potential therapeutic targets. The MTG-based gene signature and nomogram may serve to improve the individualized prediction of survival, assisting in clinical decision-making.

## Introduction

Pancreatic ductal adenocarcinoma (PDAC) is a lethal type of cancer with a five-year overall survival (OS) of less than 10% 1. Lacking typical clinical manifestations and sensitive screening methods at the early stages, PDAC is the fourth leading cause of cancer-related deaths in the USA, and by 2030, it is predicted to become the second most common cause of cancer-related deaths in the USA because of its globally rising mortality rate 2. Surgical resection continues to be the only radical treatment option; however, less than 20% of patients are eligible for this procedure 3.

Individualized systemic therapy is now recommended for PDAC, and it is therefore necessary to establish an effective individual prognostic predictive model. To date, models based on clinical and pathological parameters, such as the AJCC staging system, have been applied to evaluate prognosis 4; however, they cannot be dynamically adjusted as the patient condition changes. In addition, the AJCC staging system does not reflect the biological behavior of tumors at the molecular level. It is, therefore, necessary to develop new more accurate and personalized prognostic prediction tools for patient survival. By utilizing public databases, such as TCGA and GEO, multiple prediction models based on gene expression have been established that can reliably predict prognosis of PDAC patients following surgical resection and reflect the biological behavior of the disease 5, 6. Recently, Zhou C et al. established a robust six-gene signature to predict the OS of PDAC through comprehensive mining of OS-related DEGs 7. Rigorous validations revealed that the proposed gene signature was able to distinguish high-risk and low-risk patients with significantly different OS. Therefore, mining genes related to the prognosis of PDAC is an effective strategy to establish prediction models.

A significant proportion of PDAC patients are diagnosed at a progressive stage, with established invasion and metastasis of important nearby structures, causing them to be ineligible for surgical interventions 8. Metastasis is a biological process involving multiple steps leading to cancer cell dissociation from the primary site, invasion via the extracellular matrix to the blood or lymphatic system, and extravasation and colonization at distant organs 9. Epithelial-to-mesenchymal transition (EMT), cytoskeletal reorganization, invadopodium formation, increased cellular motility, and extracellular remodeling of the basement membrane are the primary metastasis-associated features. Metastasis at a very early stage is one of the main biological characteristics of PDAC, which is also the leading cause of its poor prognosis. Hence, gene prediction models based on metastasis-related genes (MTGs) may accurately reflect the metastatic behavior of PDAC, thus aiding the accurate prediction of patient prognosis.

In this study, we integrated four GEO gene expression datasets of PDAC to identify genes with differential expression. In combination with the Human Cancer Metastasis Database (HCMDB), differentially expressed MTGs (DE-MTGs) in PDAC were identified. A prognostic gene prediction model was proposed, prognostic factors were identified, and a prognostic nomogram was established. The relevance of the MTG-based gene signature with tumor immunity was also evaluated. The resulting novel MTG-based gene signature and the nomogram may provide a powerful tool for evaluating the OS of patients with PDAC.

## Materials and Methods

### Acquisition of TCGA clinical samples and expression data

Normalized RNA sequencing data (transcripts per million, TPM) and corresponding clinical and pathological data of pancreatic cancer were obtained from TCGA (https://portal.gdc.cancer.gov/) up to March, 20th, 2020. A total of 150 PDAC cases with pairwise tumor samples were selected based on the official TCGA publication 10. After removing four cases with history of metastasis, and an additional five with follow-up ≤ 30 days, 141 cases with tumor samples and clinical data were ultimately included in analysis. Genes were regarded to be expressed in the tissue when TPM was above 0.5. Cbioportal database (http://www.cbioportal.org/) was used to evaluate the mutation and copy number variation of tumor tissues.

### Integrated analysis of GEO gene expression datasets and identification of DE-MTGs

To identify DEGs in PDAC, the GEO database (https://www.ncbi.nlm.nih.gov/geo/) was used to identify PDAC datasets with mRNA expression and clinical data. "Pancreatic cancer", "PDAC", and "pancreatic adenocarcinoma" were used as keywords. Only datasets generated using the GPL570 platform (Affymetrix Human Genome U133 Plus 2.0 Array) and providing raw data as CEL files were included to minimize inter-platform variation. Human pancreatic tumor tissue and normal control samples were further selected. Research with primary focus on "cell lines" and "xenografts" were excluded. Ultimately, four independent array datasets (GSE15471, GSE16515, GSE32676, and GSE22780) containing 108 tumor and 70 non-tumor samples were selected for identification of DEGs in PDAC. A list of MTGs was also derived via the Human Cancer Metastasis Database (HCMDB) 11. The four datasets were then merged into a meta-dataset to increase sample size. Raw gene expression data was imported into R Bioconductor using the affy software package 12, normalized and background-corrected using the robust multi-array average (RMA) function. Batch effects were removed using the ComBat function of the inSilicoMerging package. An officially provided annotation file from the manufacturers was used to match probes with gene symbols. If multiple probes matched one single gene symbol, the median ranking value was then used. DEGs were identified using LIMMA R package 13 with cut-off values of *p* < 0.05, false discovery rate (FDR) < 0.05 and |Log2FC| > 1. DE-MTGs in PDAC were identified after intersection with the DEGs identified. GEPIA (http://gepia.cancer-pku.cn), an interactive web server for analyzing RNA sequencing expression data from 9,736 tumors and 8,587 normal samples from TCGA and Genotype-Tissue Expression (GTEx) projects 14, was used for external validation of DE-MTGs.

### Bioinformatics analysis of DE-MTGs

Potential biological processes, cellular components, molecular functions, and significantly relevant signal pathways of DEGs were explored with Gene Ontology and KEGG enrichment analyses using DAVID (https://david.ncifcrf.gov/) 15. A *p* < 0.05 was regarded as statistically significant.

### Survival analysis and establishment of a prognostic MTG-based gene signature

TCGA-PAAD dataset was used to evaluate associations between DE-MTGs and OS. Normalized gene expression data were base-2 logarithm transformed. Cox regression model was used to identify OS-related DE-MTGs in TCGA dataset. DE-MTGs with *p* < 0.05 were further used to establish a prognostic gene signature. To select prognostic DE-MTGs, Lasso penalized Cox regression analysis was applied and a prognostic gene signature was established in patients with PDAC based on a linear combination of the regression coefficient derived from the Lasso-Cox regression model coefficients (β) multiplied by its normalized mRNA expression. Optimal cut-off value of the MTG-based gene signature was determined using X-Tile 16. Patients were then separated into low and high-risk groups. Area under the curve (AUC) of the receiver operating characteristic (ROC) curve, Kaplan-Meier analysis, and Harrell's concordance index were used to assess the power of the MTG-based gene signature. ROC analysis was performed using the 'timeROC' R package and the statistical differences in the AUCs were compared using the methods of Delong et al. 17. The GSE62452 dataset and PACA-AU dataset of the International Cancer Genome Consortium (ICGC) with survival information were used for external validation 18, 19. The same formula was used to calculate a risk score for each case.

### Identification of independent prognostic parameters in PDAC

Univariate and multivariate Cox regression analyses were performed on the MTG-based gene signature and clinical parameters, including *KRAS, TP53, CDKN2A, SMAD4, BRCA1,* and *BRCA2* mutations, sex, age, tumor size, anatomical site, Grade, T stage, N stage, AJCC stage, histological subtype, residual tumor status, surgical treatment, history of radiation therapy, targeted molecular therapy, chemotherapy, tobacco smoking, alcohol consumption, chronic pancreatitis, diabetes, and prior malignancy in TCGA dataset. Parameters with *p* < 0.25 in the univariate analysis were further included in the multivariate Cox regression analysis to identify independent prognostic parameters of PDAC and to validate the prognostic role of the MTG-based gene signature. A* p* < 0.05 was regarded statistically significant.

### Development and verification of a prognostic nomogram

After performing a test of collinearity, a prognostic nomogram predicting 1-year, 2-year, and 3-year OS of PDAC patients was established based on independent prognostic parameters and relevant clinical parameters using stepwise Cox regression model. ROC curve, Kaplan-Meier analysis, C-index and calibration plots were used to assess the predictive power of the prognostic nomogram. Kaplan-Meier analysis was used to evaluate the ability to differentiate patients with different OS risk. Patients were separated into two groups according to the optimal cut-off values determined by X-Tile according to the total points of the nomogram. C-index was calculated using 1000 resamples of a bootstrap method. A calibration curve was plotted to demonstrate the predicted against observed OS.

### GSEA and analysis of tumor immunity

Potential mechanisms of the MTG-based gene signature were explored using GSEA 20. Samples from TCGA dataset were separated into high and low-risk groups based on the optimal cut-off value determined by X-Tile. JavaGSEA v3.0 was then applied to the Molecular Signatures Database v6.2 including C2: curated gene sets, C5: GO gene sets and C6: oncogenic signatures to identify enriched KEGG pathways, GO terms and dis-regulated oncogenic signatures related to poor survival of the high-risk group. FDR < 0.05 with |NES| > 1 were regarded as significantly enriched.

Stromal, immune, and estimate scores were calculated to evaluate tumor purity and immune cell infiltration in tumor tissues using ESTIMATE (Estimation of STromal and Immune cells in MAlignant Tumor tissues using expression data) algorithm based on the tumor expression data (https://bioinformatics.mdanderson.org/public-software/estimate/) 21. The proportion of 22 human hematopoietic cell phenotypes in PDAC tumor tissues (including seven T cell types, naïve and memory B cells, plasma cells, NK cells, and myeloid subsets) were further analyzed with the CIBERSORT algorithm (https://cibersort.stanford.edu/) 22. To identify associations between DE-MTGs and tumor immunity in PDAC, Pearson coefficients of correlation were calculated. A *p* < 0.05 was regarded as statistically significant.

### Statistical analysis

Statistical analysis was performed based on R software v3.6.1 (https://www.r-project.org/) and GraphPad Prism v8.01 (https://www.graphpad.com/). Categorical variables were analyzed using χ^2^ test or Fisher's exact test. Continuous variables for paired samples were analyzed using Student's *t* test. Multiple groups of continuous variables were analyzed using one-way ANOVA. Survival analysis was performed based on the univariate and multivariate Cox regression. To identify DE-MTGs associated with OS, hazard ratio (HR) and 95% confidence interval (CI) were measured. Pearson coefficient of correlation was calculated to measure the correlation between two variables. Unless stated otherwise, two-tailed *p* < 0.05 was regarded as statistically significant.

## Results

### Identification of DE-MTGs

**Figure 1:**
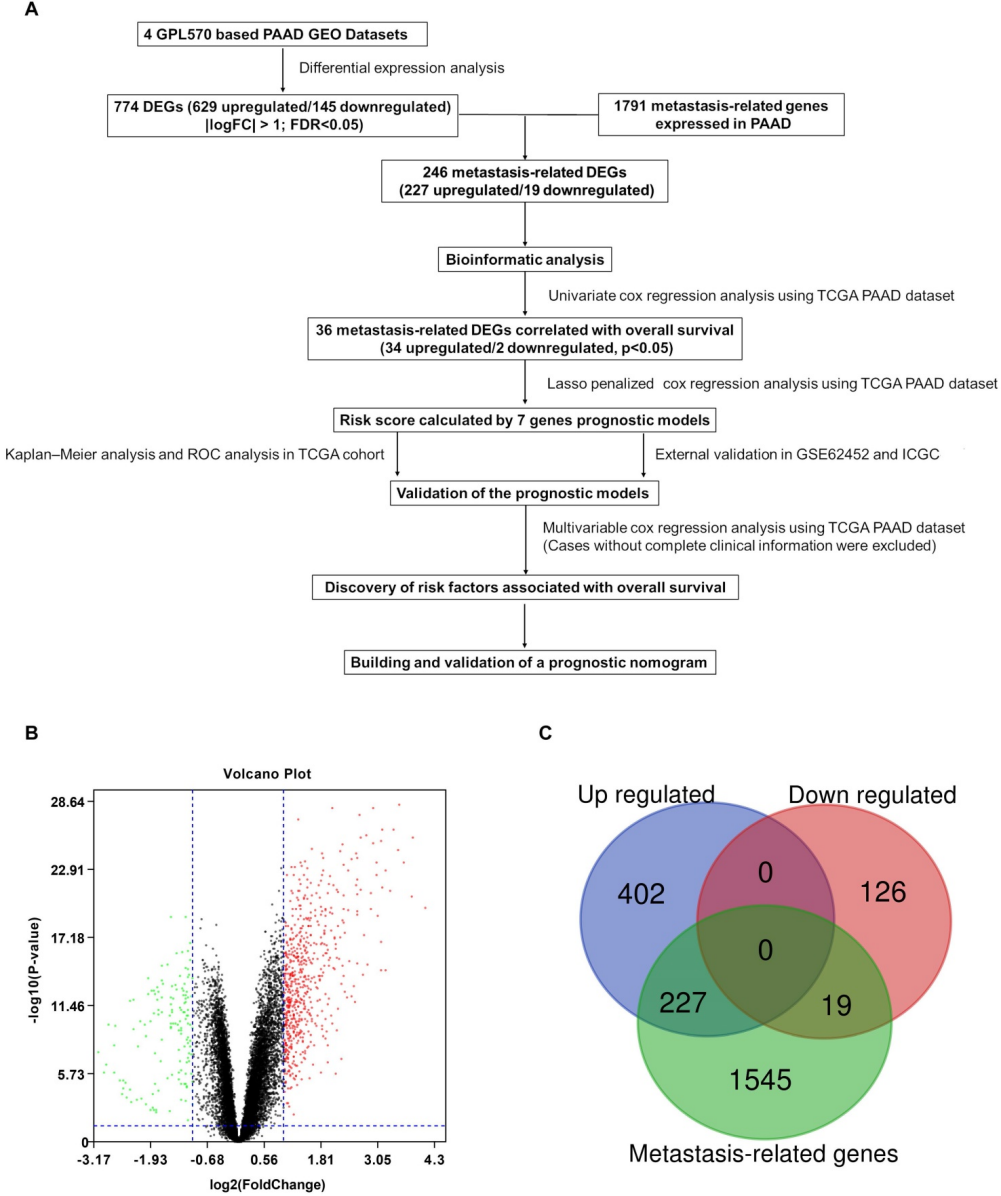
Identification of differentially expressed metastasis-related genes (DE-MTGs) in pancreatic ductal adenocarcinoma (PDAC). (**A**) Flowchart describing the process of establishment of an MTG-based gene signature and prognostic nomogram in PDAC. (**B**) Differential expression of genes between tumor and normal tissue in PDAC after the integrated analysis of the GEO datasets. (**C**) A total of 246 DE-MTGs in PDAC (including 227 upregulated and 19 downregulated) were identified based on the intersection between GEO result and potential MTGs derived from the Human Cancer Metastasis Database (HCMDB).

A flowchart of the study is described in Figure 1A. Detailed information on the GEO datasets used is presented in Table 1 18, 19, 23-25. A total of 774 DEGs, including 629 upregulated and 145 downregulated, were identified between tumor and normal pancreatic tissues (Figure 1B and Table S1). A total of 1791 MTGs with expression levels ≥ 0.5 TPM were selected (Table S2). After intersection with the identified DEGs, 246 DE-MTGs were identified (Figure 1C and Table S3), of which 227 were upregulated and 19 were downregulated.

### Functional enrichment analysis of DE-MTGs

**Figure 2:**
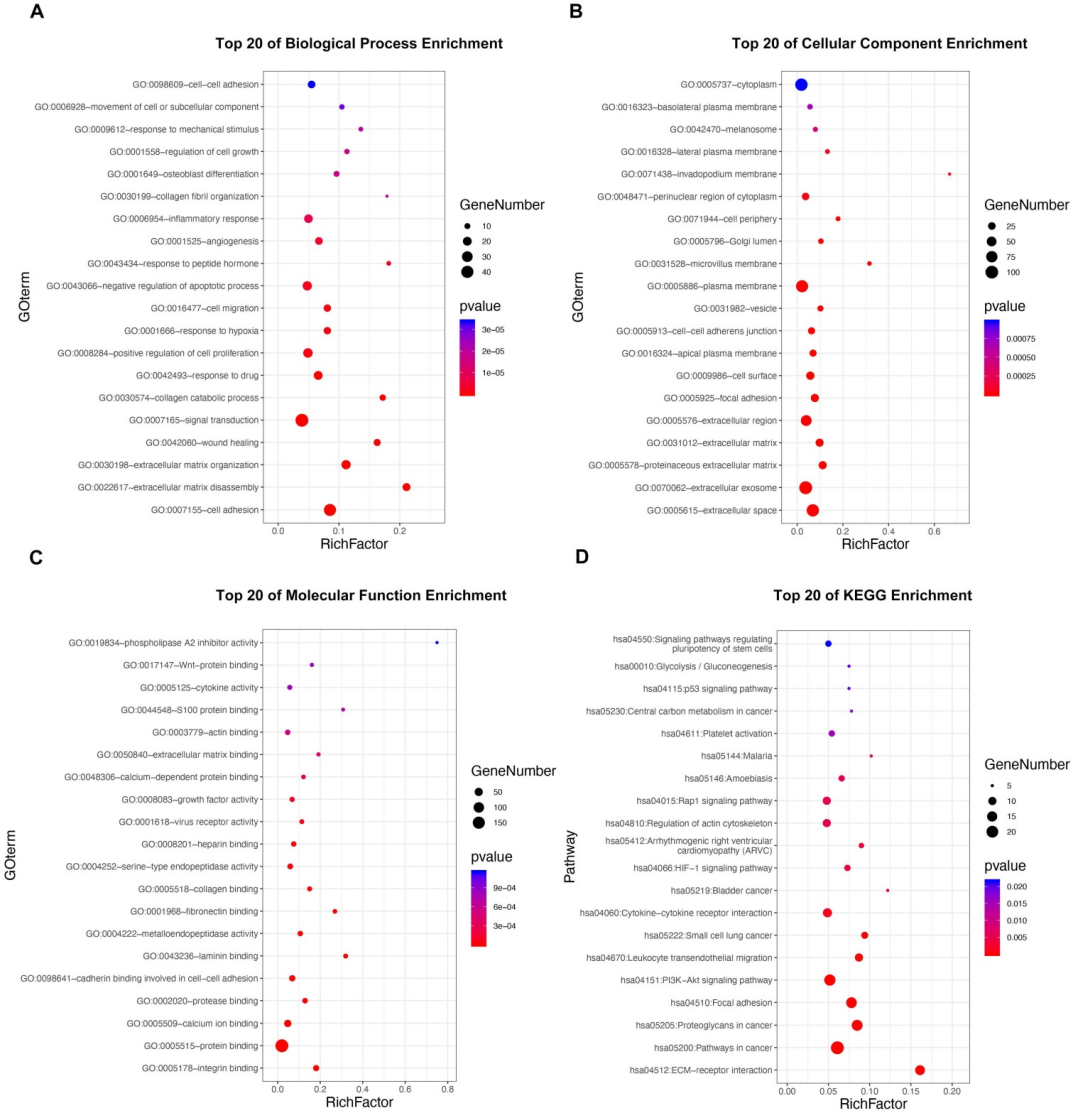
Functional enrichment analysis of the DE-MTGs. (**A**) Top 20 enriched biological processes of the DE-MTGs in PDAC. (**B**) Top 20 enriched cellular components of the DE-MTGs in PDAC. (**C**) Top 20 enriched molecular functions of the DE-MTGs in PDAC. (**D**) Top 20 enriched pathways of the DE-MTGs in PDAC. *p* < 0.05 was considered statistically significant.

GO and KEGG pathway enrichment analyses were applied to discover potential functions and relevant pathways of the 295 DE-MTGs (Figure 2A-2D and Table S4). In terms of biological process, identified DE-MTGs were most significantly enriched in migration and invasion of cancer, including cell adhesion, extracellular matrix disassembly and organization, wound healing, collagen catabolic process, collagen fibril organization, and movement of cell or subcellular components (Figure 2A). They were also significantly enriched in biological processes associated with other malignant properties of PDAC, including positive regulation of cell proliferation, response to hypoxia, angiogenesis, and negative regulation of apoptotic processes. Further, DE-MTGs were significantly enriched in immune-related processes such as negative regulation of the T cell receptor signaling pathway. KEGG analysis further revealed that the metastasis-related DEGs primarily participated in the ECM-receptor interaction and in the PI3K-Akt, HIF-1, Rap1, and p53 signaling pathways (Figure 2D).

### Identification of DE-MTGs associated with OS and establishment of a prognostic MTG-based gene signature

**Figure 3:**
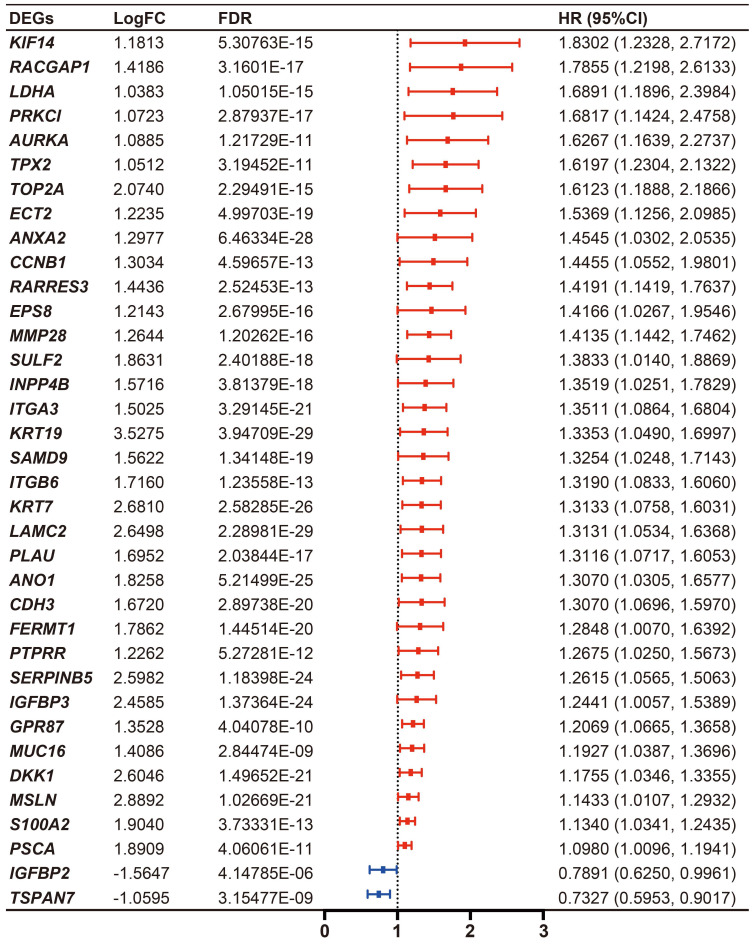
Differential expression level and forest plot of hazard ratio (HR) presenting the prognostic values of the 36 DE-MTGs associated with overall survival in PDAC.

**Figure 4:**
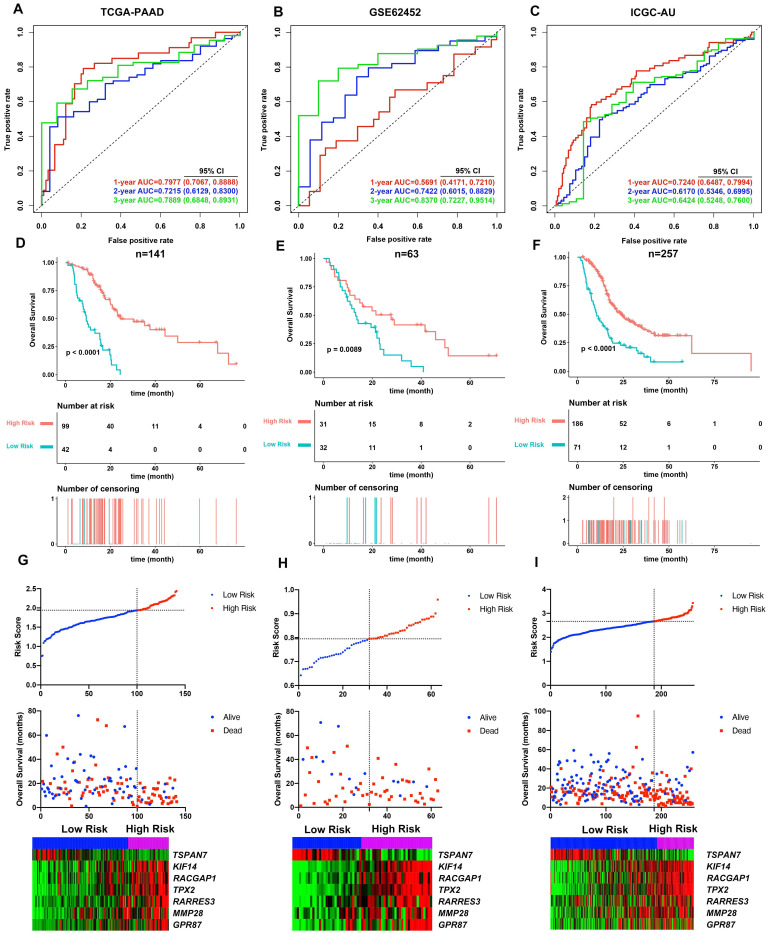
Evaluation of the performance of the MTG-based gene signature in TCGA-PAAD dataset and external validation in the GSE62452 and ICGC datasets. (**A**) Time-dependent ROC for 1-, 2- and 3-year predictions of overall survival for the MTG-based gene signature in the TCGA-PAAD dataset. (**B**) Time-dependent ROC for 1-, 2- and 3-year predictions of overall survival for the MTG-based gene signature in GSE62452. (**C**) Time-dependent ROC for 1-, 2- and 3-year predictions of overall survival for the MTG-based gene signature in ICGC. (**D**) Kaplan-Meier survival curves of the MTG-based gene signature. Patients from TCGA-PAAD dataset are stratified into two groups according to the optimal cut-off values for the risk scores determined by X-Tile software. (**E**) Kaplan-Meier survival curves of the MTG-based gene signature. Patients from the GSE62452 dataset are stratified into two groups according to the optimal cut-off values for the risk scores determined by X-Tile software. (**F**) Kaplan-Meier survival curves of the MTG-based gene signature. Patients from the ICGC dataset are stratified into two groups according to the optimal cut-off values for the risk scores determined by X-Tile software. (**G**) Relationship between the risk score (upper), survival status of patients in different groups (middle), and the expression profiles of the seven prognostic DE-MTGs (bottom) in TCGA-PAAD dataset. (**H**) Relationship between the risk score (upper), survival status of patients in different groups (middle), and the expression profiles of the seven prognostic DE-MTGs (bottom) in the GSE62452 dataset. (**I**) Relationship between the risk score (upper), survival status of patients in different groups (middle), and the expression profiles of the seven prognostic DE-MTGs (bottom) in the ICGC dataset.

Cumulatively, 141 PDAC cases were included in the survival analysis from TCGA dataset with follow-up >30 days; for whom, the baseline clinical information is presented in Table 2. A total of 36-OS related DE-MTGs were identified (Figure 3) and a prognostic gene signature consisting of seven DE-MTGs, Rac GTPase-activating protein 1 (*RACGAP1*), retinoic acid receptor responder protein 3 (*RARRES3*), targeting protein for Xklp2 (*TPX2*), matrix metalloproteinase-28 (*MMP28*), G-protein coupled receptor 87 (*GPR87*), tetraspanin-7 (*TSPAN7*), and kinesin-like protein KIF14 (*KIF14*), was constructed using LASSO-COX regression (Figure S1). Among these DE-MTGs, upregulated *RACGAP1, RARRES3, TPX2, MMP28, GPR87,* and *KIF14* with HR > 1 were regarded as oncogenes, whereas downregulated *TSPAN7* with HR < 1 was regarded as a tumor suppressor. The following formula was then used to calculate the risk score: [(0.00235)*expression value of *RACGAP1*] + [(0.11562)*expression value of *RARRES3*] + [(0.11356)*expression value of *TPX2*] + [(0.07972)*expression value of *MMP28*] + [(0.00972)*expression value of *GPR87*] - [(0.07109)*expression value of *TSPAN7*] + [(0.09209)*expression value of *KIF14*]. The optimal cut-off value was determined with X-Tile, and patients were separated into high and low-risk groups accordingly. The high-risk group was identified to have significantly poorer survival using Kaplan-Meier analysis (*p* < 0.0001; Figure 4D). Time-dependent ROC and C-index were then used to assess the predictive power of the MTG-based gene and the resulting AUCs of 1-year, 2-year, and 3-year OS prediction of the MTG-based gene signature were 0.798 (95% CI: 0.707-0.889), 0.722 (95% CI: 0.613-0.830), and 0.789 (95% CI: 0.685-0.893), respectively (Figure 4A). The C-index for the risk score was 0.690 (95% CI: 0.632-0.749). Expression of the seven DE-MTGs changed with increasing risk score. The correlation between the risk scores, gene expression data, and OS are presented in Figure 4G.

The predictive power of the MTG-based gene signature on OS was further assessed using the AJCC staging system and three previously proposed gene signatures as references. The AUCs of 1-year and 3-year OS prediction of the MTG-based gene signature were significantly higher (*p* < 0.05) than those of the AJCC staging system (Figures S2A-C). Regarding previously proposed gene signatures, the MTG-based gene signature had a significantly higher AUC in predicting 1-year OS than the gene signatures proposed by Chen H et al. and Liao X et al., higher AUC in predicting 2-year OS than the gene signature proposed by Liao X et al., and higher AUC in predicting 3-year OS than all three gene signatures (*p* < 0.05) (Figures S2A-C).

### Validating the performance of the MTG-based gene signature performance in external dataset

The MTG-based gene signature was then assessed in the external datasets with survival information GSE62452 and ICGC datasets. The same formula was applied to calculate the risk score for each case. Using X-Tile the optimal cut-off value was calculated for each dataset. Patients were then divided accordingly into high and low-risk groups. Kaplan-Meier survival curves identified significantly worse prognosis in the high-risk group (Figure 4E and 4F).

The prognosis predictive power of the MTG-based gene signature was also evaluated using ROC curve and C-index. In the GSE62452 dataset, the AUCs of 1-year, 2-year, and 3-year OS prediction of the MTG-based gene signature were 0.569 (95% CI: 0.417-0.721), 0.742 (95% CI: 0.602-0.883), and 0.837 (95% CI: 0.723-0.951), respectively (Figure 4B), while the C-index of the MTG-based gene signature was 0.570 (95% CI: 0.463-0.678). Furthermore, in the ICGC dataset, the AUCs of 1-year, 2-year, and 3-year OS prediction of the MTG-based gene signature were 0.724 (95% CI: 0.649-0.799), 0.617 (95% CI: 0.535-0.700), and 0.642 (95% CI: 0.525-0.760), respectively (Figure 4C), and the C-index of the MTG-based gene signature was 0.650 (95% CI: 0.549-0.750). The correlation between the risk scores, gene expression data, and OS are presented in Figure 4H-I.

The predictive power of the MTG-based gene signature was also assessed using the AJCC staging system and three previously proposed gene signatures as references. In the GSE62452 dataset, ROC analysis revealed that the MTG-based gene signature was comparable to the AJCC staging system and gene signatures proposed by Zhou C and Chen H, and had significantly higher AUCs than the gene signature proposed by Liao X et al. in terms of predicting the 2-year, and 3-year OS (*p* < 0.05) (Figure S2D-S2F). Additionally, in the ICGC dataset, the MTG-based gene signature had a significantly higher AUC in predicting 1-year OS than all three gene signatures, as well as a higher AUC in predicting 3-year OS than the gene signature proposed by Liao X et al. (*p* < 0.05) (Figure S2G-I).

### Validation of gene expression and genetic alterations of the seven DE-MTGs

**Figure 5:**
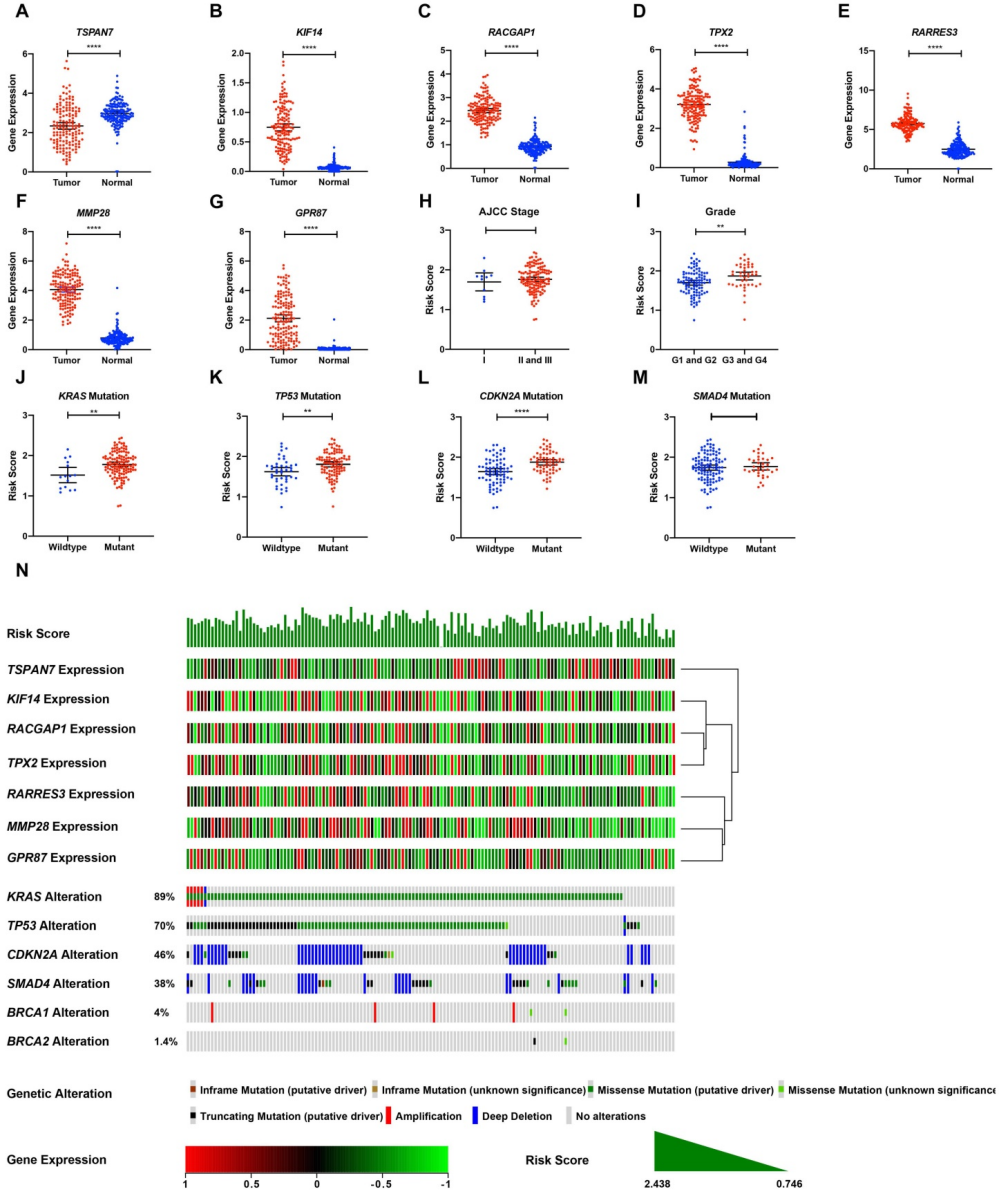
Expression levels of the seven DE-MTGs in PDAC and the mutation landscape of PDAC. (**A-G**) External validation of differential mRNA expression of the seven DE-MTGs in TCGA PADC tumor tissue and matching normal tissue from TCGA and GTEx data using GEPIA (http://gepia.cancer-pku.cn/). (**H, I**) Distribution of the MTG-based gene signature in different AJCC stages and grades in TCGA-PAAD dataset. (**J-M**) Distribution of the MTG-based gene signature for different mutation statuses of *KRAS*, *TP53, CDKN2A,* and *SMAD4* in TCGA-PAAD dataset. (**N**) The relationship among the MTG-based gene signature, transcriptome profiles and mutational profiles (*KRAS, TP53, CDKN2A, SMAD4, BRCA1*, and *BRCA2*) of PDAC. Data were obtained from the cBioPortal for Cancer Genomics (https://www.cbioportal.org). * *p* < 0.05, ** *p* < 0.01. *** *p* < 0.001. **** *p* < 0.0001.

TCGA tumor samples with matched TCGA normal and GTEx data were used to verify the differential expression of the seven DE-MTGs using GEPIA. Consistent with the results of integrated analysis, mRNA expression of *RACGAP1, RARRES3, TPX2, MMP28, GPR87*, and *KIF14* was significantly upregulated in PDAC tumor tissues, whereas *TSPAN7* was significantly downregulated (Figure 5A-G). Although the risk scores were comparable between stage II and III patients versus stage I patients, pathological grade 3 and 4 patients had significantly higher risk scores than did grade 1 and 2 patients (Figure 5H-I). The association between the seven-gene signature and the most frequent mutations in PDAC (*KRAS, TP53, CDKN2A,* and* SMAD4*) was further analyzed. Risk scores of PDAC cases with *KRAS, TP53*, and* CDKN2A* mutations were significantly higher than cases with wild-type genes. In contrast, the risk score was comparable between cases with and without *SMAD4* mutation (Figure 5J-M). The relationship between the transcriptome profiles, mutational profiles (*KRAS, TP53, CDKN2A, SMAD4, BRCA1,* and* BRCA2)* of PDAC and the MTG-based gene signature were further analyzed and are presented in Figure 5N.

### Assessment of prognostic parameters related to OS in PDAC

A total of 77 patients from TCGA dataset with complete clinical data were included for further analysis (Table 3, Table S5). Patients in the high-risk group showed significantly shorter OS (*p* < 0.001), larger tumor size (*p* = 0.013), and a greater portion of microscopic or macroscopic residual tumor (*p* = 0.017) (Table 3). Prognostic factors related to OS in PDAC were identified using univariate and multivariate Cox regression. Risk score, tumor size, *CDKN2A* mutation,* BRCA1* mutation, N stage, residual tumor, history of radiation therapy, targeted molecular therapy, and chemotherapy were identified to be significantly related to OS of PDAC in univariate Cox analysis, with *p* < 0.05 (Table 4). The multivariate Cox analysis further integrated parameters with *p* < 0.25 in the univariate analysis and found that risk score (*p* = 0.0037), history of targeted molecular therapy (*p* = 0.0260) and diabetes (*p* = 0.0141) were independent risk factors of OS in PDAC (Table 5).

### Establishment and validation of a prognostic nomogram

**Figure 6:**
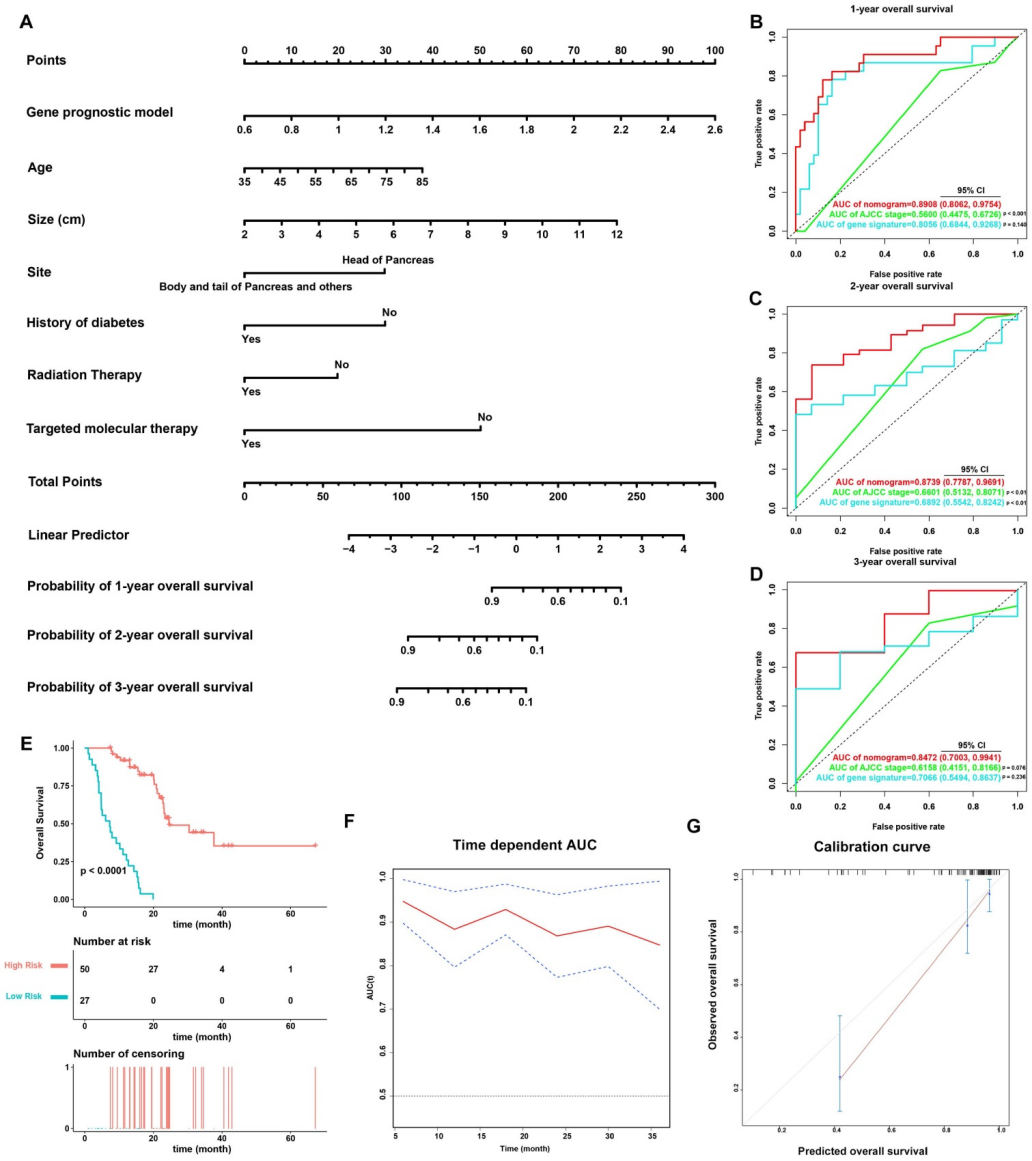
Validation of the nomogram in predicting overall survival of PDAC in TCGA-PAAD dataset. (**A**) A prognostic nomogram incorporating MTG-based gene signature predicting 1-, 2- and 3-year overall survival of PDAC. (**B-D**) Prognostic performance of the gene signature-based nomogram, MTG-based gene signature and AJCC staging system using time-dependent ROC for predicting the 1-, 2- and 3-year overall survival of PDAC. (**E**) Kaplan-Meier survival curve of the nomogram. Patients from TCGA-PAAD dataset are stratified into two groups of different level of risk according to the optimal cut-off value for the nomogram determined by X-Tile software. (**F**) The time-dependent AUC of the nomogram in predicting overall survival of PDAC. (**G**) The calibration plot for internal validation of the nomogram. The Y axis represents the actual overall survival, whereas the X axis represents the predicted overall survival.

On the basis of the 77 patients with complete clinical details from TCGA-PAAD dataset, a prognostic nomogram predicting 1-year, 2-year, and 3-year OS of PDAC patients was created using a stepwise model of Cox regression. Risk score, age, tumor size, tumor site, history of diabetes, history of radiation therapy, and history of targeted molecular therapy were parameters included in the nomogram (Figure 6A). The AUCs of 1-year, 2-year, and 3-year OS were 0.891 (95% CI: 0.806-0.975), 0.874 (95% CI: 0.779-0.969), and 0.847 (95% CI: 0.700-0.994) (Figure 6B-6D), and the C-index was 0.828 (95% CI: 0.781-0.874). Based on the cut-off value calculated using X-Tile, the patients were divided into two groups of different prognostic risk. Patients with higher risk scores were associated with poorer prognosis (Figure 6E). Calibration curves further revealed that the predicted OS using nomogram was similar to that observed for OS (Figure 6G).

The predictive power of the nomogram was also compared against the AJCC staging system (Figure 6B-D). The AUCs of 1-year, 2-year, and 3-year OS for the AJCC staging system were 0.560 (95% CI: 0.448-0.673), 0.660 (95% CI: 0.513-0.807), and 0.616 (95% CI: 0.415-0.817), while the C-index was 0.572 (95% CI: 0.502-0.643). The nomogram incorporating the MTG-based gene signature had significantly higher AUCs in predicting 1-year and 2-year OS of PDAC patients compared to those of the AJCC staging system (*p* < 0.05).

### GSEA

**Figure 7:**
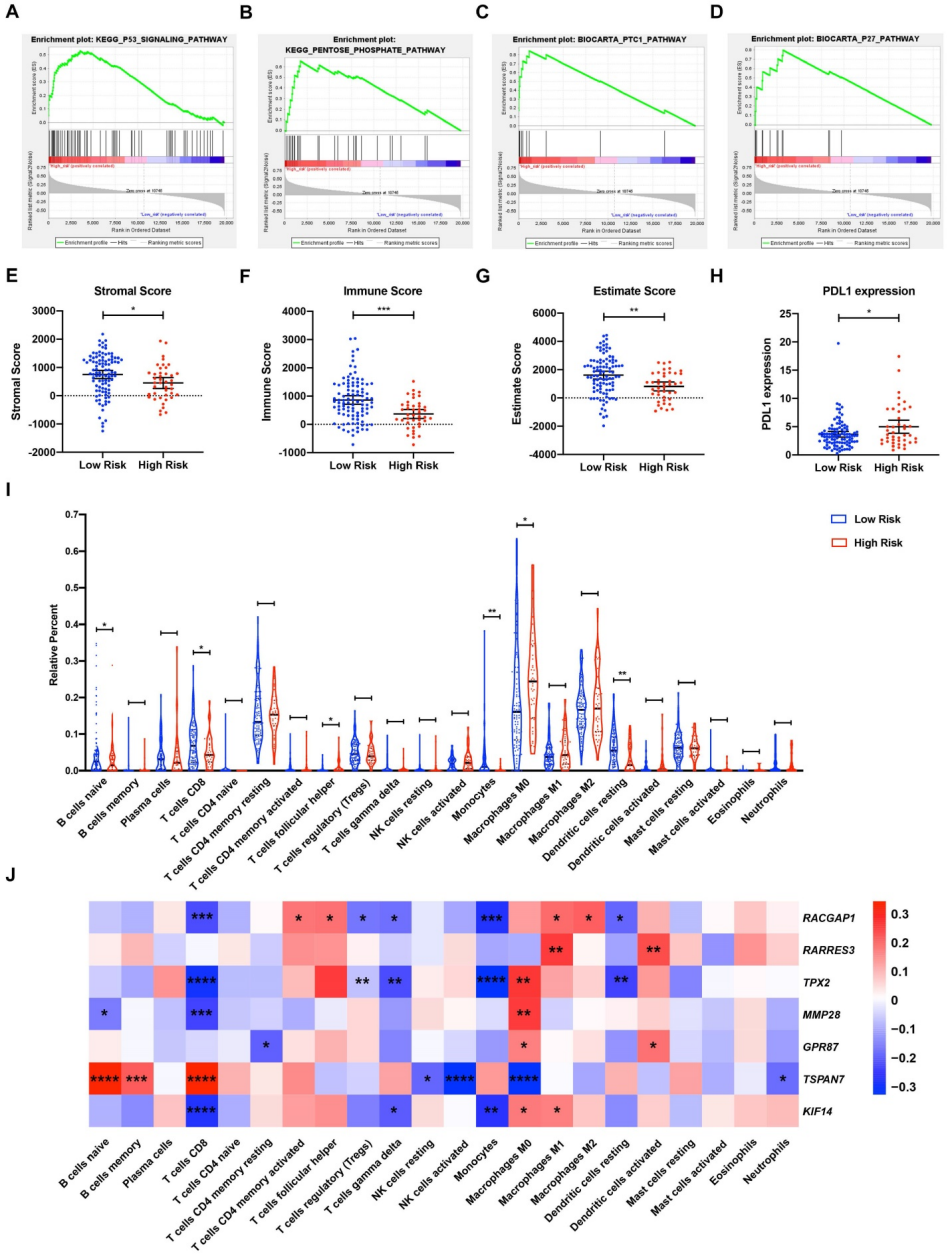
Gene Set Enrichment Analysis (GSEA) and tumor immunity analysis of the MTG-based gene signature. (**A-D**) Top signaling pathways and oncological signatures significantly enriched in the high-risk group identified by GSEA. (**E-G**) Distribution of the Stromal scores, Immune scores, and ESTIMATE scores in high-risk and low-risk groups from TCGA-PAAD dataset. The Stromal scores, Immune scores, and ESTIMATE scores were calculated using the ESTIMATE algorithm (https://bioinformatics.mdanderson.org/public-software/estimate/). (**H**) Differentially expressed PDL1 between the high-risk and low-risk groups of TCGA-PAAD dataset. (**I**) Differential immune infiltrates in high-risk and low-risk groups of TCGA-PAAD dataset. The proportion of 22 immune infiltrates for each case are estimated using the CIBERSORT algorithm (https://cibersort.stanford.edu/index.php). (**J**) Correlation matrix of the relationship between the expression of the seven prognostic DE-MTGs and the differential immune infiltration levels. Pearson correlation analysis was used to calculate the correlations. Colors from blue to red present the Pearson correlation coefficient. * *p* < 0.05, ** *p* < 0.01. *** *p* < 0.001. **** *p* < 0.0001.

To explore the underlying mechanisms of the MTG-based gene signature, patients from TCGA dataset were separated into high- and low-risk groups according to the optimal cut-off value of the MTG-based gene signature determined by X-Tile. In the high-risk group, enriched KEGG pathway analysis revealed that molecular alteration was closely related to the pentose-phosphate and P53 signaling pathways. A total of 28 oncological signatures, including the pathways PTC1 and P27 were also significantly enriched (Figure 7A-D). Full GSEA analysis results are presented in Table S6.

### Analysis of tumor immunity

The relationship between risk score and tumor immunity was further analyzed. Tumor purity and the infiltration level of immune cell were estimated. Tumors in the high-risk group had significantly lower stromal, immune, and ESTIMATE scores, indicating a lower level of stroma, immune cell infiltration, and tumor purity (Figure 7E-G). Moreover, tumors in the high-risk group had significantly higher levels of *PDL1* expression (Figure 7H). To further explore the underlying molecular mechanisms of the MTG-based gene signature and their relevance to tumor immunity, the proportion of 22 immune infiltrates (naïve B cells, memory B cells, plasma cells, CD8 T cells, CD4 naïve T cells, CD4 memory resting T cells, CD4 memory activated T cells, follicular helper T cells, regulatory T cells (Tregs), gamma delta T cells, resting NK cells, activated NK cells, monocytes, M0 macrophages, M1 macrophages, M2 macrophages, resting dendritic cells, activated dendritic cells, resting mast cells, activated mast cells, eosinophils, and neutrophils) was estimated for each case using CIBERSORT. The high-risk group was found to be associated with significantly higher levels of follicular helper T cells and M0 macrophage infiltration, and lower levels of infiltrating naïve B cells, CD8 T cells, monocytes, and resting dendritic cells (Figure 7I).

Correlations between DE-MTGs gene expression and immune infiltration levels were then evaluated to identify potential immune regulators (Figure 7J). In terms of immune infiltrates upregulated in the high-risk group, the expression of *RACGAP1* was positively correlated with infiltration of follicular helper T cells (*r* = 0.1924, *p* < 0.05). The expressions of *TPX2, MMP28, GPR87*, and *KIF14* were positively correlated with M0 macrophage infiltration, whereas *TSPAN7* expression was negatively correlated with M0 macrophage infiltration (*r* = 0.2688, 0.2615, 0.1735, 0.1794, and -0.3277, respectively; *p* < 0.05). In terms of immune infiltrates downregulated in the high-risk group, *MMP28* was negatively correlated with the infiltration of naïve B cells, whereas *TSPAN7* was positively correlated with naïve B cell infiltration (*r* = -0.1658 and 0.3352, respectively; *p* < 0.05). In addition, *RACGAP1, TPX2, MMP28*, and *KIF14* were negatively correlated, while *TSPAN7* was positively correlated with the infiltration of CD8 T cells (*r* = -0.2248, -0.2975, -0.2394, -0.2790, and 0.3450, respectively; *p* < 0.05). Lastly, *RACGAP1, TPX2,* and *KIF14* were negatively correlated with monocyte infiltration, while *RACGAP1* and *TPX2* were negatively correlated with the infiltration of resting dendritic cells (*r* = -0.2787, -0.3240, -0.2714, -0.1925, and -0.2366; *p* < 0.05).

## Discussion

Traditional clinical pathological parameters, such as AJCC staging, do not accurately or dynamically reflect PDAC progress and show poor prognosis predictive capacity. Accurately predicting PDAC prognosis will allow for more aggressive treatment, earlier intervention, and delayed tumor progression. As PDAC is highly heterogeneous, its progression involves a network of multiple complex signaling pathways. Hence, molecular prognostic markers can dynamically reflect tumor progress and are quantitatively measured. Specifically, a gene signature integrating multiple gene markers may be superior compared to a single marker, in predicting biological characteristics and prognosis. Nomograms integrate multiple molecular, biological, and clinicopathological prognostic parameters, individually calculating the numerical probability of clinical events 26 and are widely used in the evaluation of oncology and clinical evaluation of prognosis. Compared with conventional staging strategies, nomograms incorporating gene signatures may prove more accurate in predicting prognosis and in providing a simpler interface for patients to understand, which is helpful in clinical decision-making.

In the current study, 246 DE-MTGs of PDAC were identified. Analysis of OS revealed that 36 DE-MTGs were closely related to the OS of PDAC. A novel MTG-based gene signature was established to predict PDAC OS in TCGA-PAAD dataset. Among these, *RACGAP1, RARRES3, TPX2, MMP28, GPR87*, and *KIF14* were upregulated and positively associated with poor survival, whereas *TSPAN7* was downregulated and identified as a tumor suppressor. The MTG-based gene signature was an independent prognostic factor of PDAC, which could distinguish patients with differential OS risk. Patients in the low-risk group had a significantly better prognosis than the high-risk group. The prognostic performance of the MTG-based gene signature was also validated in the external datasets. In addition, a prognostic nomogram predicting 1-year, 2-year, and 3-year OS of PDAC was established based on the MTG-based gene signature and clinical pathological parameters. Analysis of tumor immunity further identified significantly higher levels of follicular helper T cell and M0 macrophage infiltration, and lower infiltration of naïve B cells, CD8 T cells, monocytes, and resting dendritic cells in tumors of the high-risk group. Finally, regulatory relationships between the seven DE-MTGs and alterations in immune cell infiltration were established to explore the underlying mechanisms.

The MTG-based gene signature contains six genes previously reported to be associated with PDAC. *TPX2* is required for normal assembly of mitotic spindles and for mediating localization and activation of AURKA by promoting autophosphorylation. The expression of *TPX2* is related to the TNM stage and pathological grade of various digestive system cancers, with increased expression having been shown to be significantly associated with poorer prognosis. In PDAC, genomic hybridization and integrated analyses of RNA and DNA identified *TPX2* as a potential target for amplification in both PDAC and non-small-cell lung cancer 27. Further, *TPX2* expression is upregulated in PDAC cell lines and tumor tissues, whereas its knockdown using siRNA suppressed the growth of PDAC cells via induction of apoptosis *in vitro*. Suppression of *TPX2* expression also inhibits the growth of PDAC *in vivo* and enhances the sensitivity of pancreatic cancer cells to paclitaxel 28. In addition, *TPX2* was reported to promote tumor angiogenesis in PDAC. *TPX2* siRNA upregulated the expression of *IGFBP-3*, resulting in significantly reduced CD34-positive micro vessels in the tumor. Hence, *TPX2* siRNA may exhibit an anti-angiogenic effect partially by upregulating the expression of *IGFBP-3* 29. A recent study revealed that upregulation of* TPX2* in PDAC is associated with* KRAS* mutation, whereas suppressing *TPX2* and its target protein *AURKA* inhibits growth and migration in *KRAS*-mutant PDAC cells 30.

*GPR87* is a receptor for lysophosphatidic acid (LPA). *p53* directly upregulates GPR87 through *p53*-responsive element, and it is critical for *p53*-dependent survival in response to DNA damage 31. *GPR87* is also upregulated in PDAC cells and tissues and is related to significantly poorer prognosis and clinicopathological parameters. Further, its upregulation promotes proliferation and angiogenesis of PDAC and increases resistance to gemcitabine through the NF-κB signaling pathway 32. Hence, *GPR87* is regarded a potential target for treatment of PDAC. Bioengineered siRNA loaded nanoparticles may effectively inhibit the expression of *GPR87*, exerting a cytotoxic effect 33. The abnormal expression of *GPR87* was also reported in a variety of malignancies. For instance, it was found to be upregulated in squamous cell carcinomas of multiple organs as well as in their lymph node metastasis. In addition, *GPR87* is highly expressed in lung adenocarcinomas and transitional cell carcinoma of the bladder 34. Alternatively, inhibition of *GPR87* suppresses the migration and proliferation of lung cancer 35 Moreover, *GPR87* is overexpressed in hepatocellular carcinoma, which upregulates CD133 expression and increases cancer stem cell migration and invasion 36.

Kinesin-like protein *KIF14* is a microtubule motor protein that binds microtubules in the form of heterodimers with high affinity. In addition, *KIF14* has ATPase activity and is critical in various biological processes including cytokinesis, cell division, cell proliferation, and apoptosis. In PDAC, *KIF14* was reported to be associated with perineural invasion 37 with *KIF14* found to be upregulated in PDAC and cancer cells invading the perineural niche. Alternatively, down-regulation of *KIF14* altered the perineural invasion pattern of PDAC cells. Moreover, overexpression of *KIF14* is associated with poorer prognosis of PDAC 38. *KIF14* was identified as a candidate oncogene in the 1q minimal region of genomic gain in breast cancer, medulloblastoma, lung cancer, retinoblastoma, and renal cell carcinomas 39 with its overexpression being associated with poorer prognosis in these malignancies. *KIF14* also participates in modulating components of adhesion on the tumor cell surface, regulating migration and invasion through Rap1a-Radil signaling, thereby promoting cell motility during metastasis 40.

*RACGAP1* is a component of the central spindle in the complex mediating microtubule-dependent Rho signaling during cytokinesis. It is also upregulated in PDAC and is related to shorter OS 38; however, the associated mechanism has not yet been elucidated. *RACGAP1* is also critical in the progression of multiple cancers, with its overexpression reported to be associated with histological grade, Ki67 protein expression, and poorer survival 41. Expression of *RACGAP1* also impacts invasiveness of cancer cells resulting in significantly poorer prognosis and lymph node and distant metastasis 42. Hence, *RACGAP1* has potential of predicting metastasis in PDAC.

*RARRES3* is important in regulating EMT and metastasis of multiple cancers. Consistent with our study, previous studies revealed that it is upregulated in PDAC and is associated with poor survival 43. The oncogenic role of *RARRES3* is associated with *EPS8*, which is the target of antitumor miR-130b-5p. *RARRES3* is also an important regulator of oncogenic *RAS* signaling by binding to the hyper-variable regions of *RAS* proteins 44. In contrast, in breast cancer, *RARRES3* is a tumor suppressor. Specifically, it modulates the acylation status of *Wnt* proteins, suppressing the EMT and cancer stem cell properties in breast cancer 45. In colorectal cancer, *RARRES3* is downregulated in tumor tissues; in contrast, its upregulation inhibits metastasis through EMT regulation 46. Hence, *RARRES3* may play a two-way regulatory role in mediating metastasis, especially in the presence of different *RAS* mutation states. The role of* RARRES3* in PDAC and metastasis needs to be further elucidated.

*MMP28* degrades casein, playing a role in tissue repair and homeostasis. It also modulates cell behavior by releasing growth factors and active peptides from the extracellular matrix. In PDAC, the expression of *MMP28* is upregulated by the oncogenic protein *PARP1* via the STAT3-MMP7 axis 47. Moreover, *MMP28* is expressed in many cancers 48 and has been shown to induce EMT in lung cancer through the TGF-β signaling pathway, thereby promoting invasion 49. *MMP28* is also overexpressed in gastric cancer and is related to lymph node metastasis, tumor invasion, and poor OS 50. The role of *MMP28* in promoting metastasis via Notch3 signaling was also presented in hepatocellular carcinoma 51.

The role of *TSPAN7* in PDAC has not yet been reported. However, *TSPAN7* has been shown to participate in regulating cell proliferation and motility. Consistent with our results, higher expression of *TSPAN7* is related to longer tumor-specific survival and disease-free survival in clear-cell renal cell carcinoma. Moreover, *TSPAN7* is downregulated in metastases, indicating its anti-metastatic role 52. *TSPAN7* is also downregulated in the metastasis of uterine leiomyosarcoma compared with in the primary tumor 53. In multiple myeloma, *TSPAN7* increases cell adhesion and is associated with improved survival 54. *TSPAN7* is also downregulated in soft tissue sarcoma and is associated with better survival 55. In contrast, *TSPAN7* is overexpressed in lung cancer and promotes migration and EMT 56.

Tumor immune evasion is critical in tumor progression 57. Tumors can manipulate immune cells in the tumor microenvironment to evade surveillance of the immune system. This can be achieved by recruiting immunosuppressive cells, reducing tumor immunogenicity, or utilizing immunosuppressive mechanisms 58. In our study, follicular helper T cells and M0 macrophage infiltration was significantly upregulated in high-risk tumor tissues, whereas naïve B cell, CD8+ T cell, monocyte, and resting dendritic cell infiltration was significantly downregulated. CD8+ cytotoxic T lymphocytes recognize antigens presented by MHC and are the primary immune cells that target tumor cells. Consistent with our results, higher levels of CD8+ infiltration is associated with improved prognosis in PDAC 59. Further spatial analysis showed that there was a large heterogeneity in the density of CD8+ cells in PDAC tumor tissues. The infiltration of CD8+ cells in the tumor center was significantly lower than in the tumor margin and was associated with poorer prognosis. Tumor CD274 expression and tertiary lymphatic structure are associated with higher CD8+ cell density at the tumor margin 60. The role of CD4+ T cells and regulatory T cells in tumor immunity is more complex. On the one hand, CD4+ T cells are required for the formation of effective antitumor immunity as they promote the function of CTLs and maintenance of memory. CD4+ helper T cells also amplify the effects of T cells and B cells and help CTLs overcome negative regulation 61. On the other hand, regulatory T cells are enriched within primary and metastatic tumors and are associated with poor prognosis 62. Follicular helper T cells were previously reported to be associated with the function of CD8+ T cells 63. In our study, with the lower infiltration of CD8+ T cells, a higher infiltration of follicular helper T cells was identified in the high-risk group, which was seemingly a compensatory effect. Similarly, M0 macrophages are upregulated in stage N1 tumors of colorectal cancer, indicating their association with metastasis and progression 64. In addition, we found that many of the identified metastasis-related DEGs are significantly associated with the infiltration level of various immune cells, suggesting that a potential regulatory relationship may exist. Specifically, genes associated with metastasis are important regulators of the extracellular matrix (ECM), which affects a myriad of aspects related to tumor biology and modulates the function and recruitment of immune cells through direct effects or increased cytokine expression in the tumor microenvironment (TME) 65. We, therefore, postulate that PDAC cells may manipulate the function and infiltration levels of immune cells in the tumor microenvironment during invasion and metastasis. The cells may then subsequently recruit immunosuppressive cells and alter the secretion profile of cytokines by immune cells and TME to allow immune evasion and invasion. This hypothesis may elucidate the underlying mechanism related to the poor prognosis of patients in the high-risk group of pancreatic cancer; therefore, it deserves further experimental validation.

To the best of our knowledge, the MTG-based gene signature and associated nomogram presented herein, have not yet been reported. Our prognostic model was based on quantitative expression of a panel of genes, which is more economically and practically feasible than genome-wide sequencing. Further, our graphic scoring system of nomograms is simple for patients to understand. Nomograms combined with gene prognosis models and clinical and pathological parameters may provide clinicians with novel methods to accurately evaluate the prognosis of post-surgical PDAC patients, thus achieving personalized treatment. However, the present study has certain limitations. First, the clinical information for our dataset was primarily obtained from TCGA database, which is comprised largely of Caucasian North American patients, and thus caution should be exercised when extending our results to patients of other ethnic groups. Protein expression levels of these DE-MTGs also require further analysis. The molecular mechanisms of their involvement in metastasis and immunomodulation in PDAC depend on further experimental studies for clarification. In addition, the levels of immune cell infiltration in our study were based on algorithmic evaluation and require further experimental validation.

## Conclusions

Herein, a prognostic predictive model based on seven DE-MTGs was established for PDAC. A prognostic nomogram was also established combining the MTG-based gene signature and prognostic-related clinical and pathological parameters to predict the OS of PDAC patients. The seven DE-MTGs are significantly related to the progression and OS of PDAC and are potential targets for treatment. Moreover, significant differences in immune cell infiltration were identified between tumors in the high- and low-risk groups with the expression of multiple DE-MTGs closely associated with immune cell infiltration. The nomograms incorporating the MTG-based gene signature were determined to be superior in predicting the OS of PDAC patients, compared to current strategies, and may be useful for designing personalized therapy and medical decisions.

## Supplementary Material

Supplementary figures and tables.

## Figures and Tables

**Table 1:** Details of the datasets included in this study

Datasets	Reference	Platform	Sample size (Tumor/Control)	Application
GSE15471	Badea L et al., 2009	[HG-U133_Plus_2] Affymetrix Human Genome U133 Plus 2.0 Array	78 (39/39)	Identification of DEGs
GSE16515	Pei H et al., 2009	[HG-U133_Plus_2] Affymetrix Human Genome U133 Plus 2.0 Array	52 (36/16)	Identification of DEGs
GSE32688	Donahue TR et al., 2011	[HG-U133_Plus_2] Affymetrix Human Genome U133 Plus 2.0 Array	32 (25/7)	Identification of DEGs
GSE22780	Killary AM et al., 2011	[HG-U133_Plus_2] Affymetrix Human Genome U133 Plus 2.0 Array	16 (8/8)	Identification of DEGs
GSE62452	Yang S et al., 2016	[HuGene-1_0-st] Affymetrix Human Gene 1.0 ST Array [transcript (gene) version]	69 (69/0)	External validation
ICGC	Christopher JS et al., 2011	Illumina HumanHT-12 V4.0 expression beadchip	269 (269/0)	External validation

**Table 2:** Clinical features of PDAC patients in TCGA-PAAD dataset

Clinical features	Mean+SD
Follow up time (day)	538.05 ± 418.09
Risk Score	1.75 ± 0.32
Age	64.70 ± 11.00
Size (cm)	3.74 ± 1.37
	**N (%)**
**Survival status**	
Alive	62 (43.97%)
Dead	79 (56.03%)
***KRAS* mutation**	
Wildtype	15 (10.64%)
Mutant	126 (89.36%)
***TP53* mutation**	
Wildtype	43 (30.50%)
Mutant	98 (69.50%)
***CDKN2A* mutation**	
Wildtype	76 (53.90%)
Mutant	65 (46.10%)
***SMAD4* mutation**	
Wildtype	107 (75.89%)
Mutant	34 (24.11%)
***BRCA1* mutation**	
Wildtype	135 (95.74%)
Mutant	6 (4.26%)
***BRCA2* mutation**	
Wildtype	139 (98.58%)
Mutant	2 (1.42%)
**Sex**	
Male	75 (53.19%)
Female	66 (46.81%)
**Subtype**	
Pancreas-Adenocarcinoma Ductal Type	128 (90.78%)
Pancreas-Adenocarcinoma-Other Subtype	13 (9.22%)
**Grade**	
G1	18 (12.77%)
G2	80 (56.74%)
G3	42 (29.79%)
G4	1 (0.71%)
**T**	
T1	5 (3.55%)
T2	14 (9.93%)
T3	118 (83.69%)
T4	3 (2.13%)
Not available	1 (0.71%)
**N**	
N0	36 (25.53%)
N1	103 (73.05%)
Not available	2 (1.42%)
**M**	
M0	67 (47.52%)
Mx	74 (52.48%)
**AJCC stage**	
IA	4 (2.84%)
IB	7 (4.96%)
IIA	24 (17.02%)
IIB	101 (71.63%)
III	2 (1.42%)
Not available	3 (2.13%)
**Residual tumor**	
R0	76 (53.90%)
R1	47 (33.33%)
R2	5 (3.55%)
Not available	13 (9.22%)
**Site**	
Head of Pancreas	113 (80.14%)
Body of Pancreas	11 (7.80%)
Tail of Pancreas	8 (5.67%)
Others	9 (6.38%)
**Initial pathologic diagnosis method**	
Tumor resection	88 (62.41%)
Tissue Biopsy	25 (17.73%)
Cytology (e.g. Peritoneal or pleural fluid)	18 (12.77%)
Fine Needle Aspiration Biopsy	5 (3.55%)
Not available	5 (3.55%)
**Surgical treatment**	
Whipple	112 (79.43%)
Distal Pancreatectomy	17 (12.06%)
Distal Pancreatectomy & laporoscopy followed by Hand-assisted and Splenectomy	1 (0.71%)
Subtotal pancreatectomy and splenectomy and cholecystectomy	1 (0.71%)
Radical pancreaticoduodenectomy	4 (2.84%)
Total Pancreatectomy	2 (1.42%)
Endoscopic Retrograde Cholangiopancreaticography	1 (0.71%)
Not available	3 (2.13%)
**History of neoadjuvant treatment**	
No	141 (100.00%)
**History of chemotherapy**	
No	43 (30.50%)
Yes	98 (69.50%)
**History of radiation therapy**	
No	81 (57.45%)
Yes	28 (19.86%)
Not available	32 (22.70%)
**History of targeted molecular therapy**	
No	35 (24.82%)
Yes	97 (68.79%)
Not available	9 (6.38%)
**Tobacco smoking history**	
Lifelong Non-smoker	50 (35.46%)
Current smoker	16 (11.35%)
Current reformed smoker for > 15 years	23 (16.31%)
Current reformed smoker for ≤ 15 years	21 (14.89%)
Current reformed smoker, duration not specified	7 (4.96%)
Not available	24 (17.02%)
**Alcohol drinking history**	
No	49 (34.75%)
Yes	81 (57.45%)
Not available	11 (7.80%)
**History of chronic pancreatitis**	
No	103 (73.05%)
Yes	13 (9.22%)
Not available	25 (17.73%)
**History of diabetes**	
No	90 (63.83%)
Yes	31 (21.99%)
Not available	20 (14.18%)
**History of prior malignancy**	
No	128 (90.78%)
Yes	13 (9.22%)

**Table 3:** Baseline characteristics of patients included for the evaluation of prognostic factors and establishment of nomogram

Clinical features	Low risk	High risk	*p*-value
	**Mean ± SD**		
Overall Survival (day)	625.75 ± 352.45	282.04 ± 177.79	< 0.001
Risk Score	1.63 ± 0.25	2.11 ± 0.15	< 0.001
Age	63.90 ± 12.09	63.73 ± 11.06	0.952
Size (cm)	3.40 ± 1.07	4.29 ± 2.00	0.013
	**N (%)**		
**Survival status**			< 0.001
Alive	27 (52.94%)	3 (11.54%)	
Dead	24 (47.06%)	23 (88.46%)	
***KRAS* mutation**			0.787
Wildtype	7 (13.73%)	3 (11.54%)	
Mutant	44 (86.27%)	23 (88.46%)	
***TP53* mutation**			0.209
Wildtype	19 (37.25%)	6 (23.08%)	
Mutant	32 (62.75%)	20 (76.92%)	
***CDKN2A* mutation**			0.164
Wildtype	32 (62.75%)	12 (46.15%)	
Mutant	19 (37.25%)	14 (53.85%)	
***SMAD4* mutation**			0.555
Wildtype	36 (70.59%)	20 (76.92%)	
Mutant	15 (29.41%)	6 (23.08%)	
***BRCA1* mutation**			0.073
Wildtype	50 (98.04%)	23 (88.46%)	
Mutant	1 (1.96%)	3 (11.54%)	
***BRCA2* mutation**			0.306
Wildtype	49 (96.08%)	26 (100.00%)	
Mutant	2 (3.92%)	0 (0.00%)	
**Sex**			0.945
Male	29 (56.86%)	15 (57.69%)	
Female	22 (43.14%)	11 (42.31%)	
**Subtype**			0.179
Pancreas-Adenocarcinoma Ductal Type	44 (86.27%)	25 (96.15%)	
Pancreas-AdenocarcinomaOther Subtype	7 (13.73%)	1 (3.85%)	
**Grade**			0.331
G1	4 (7.84%)	0 (0.00%)	
G2	31 (60.78%)	15 (57.69%)	
G3	15 (29.41%)	11 (42.31%)	
G4	1 (1.96%)	0 (0.00%)	
**T**			0.223
T1	4 (7.84%)	0 (0.00%)	
T2	6 (11.76%)	1 (3.85%)	
T3	40 (78.43%)	25 (96.15%)	
T4	1 (1.96%)	0 (0.00%)	
**N**			0.195
N0	17 (33.33%)	5 (19.23%)	
N1	34 (66.67%)	21 (80.77%)	
**M**			0.471
M0	29 (56.86%)	17 (65.38%)	
Mx	22 (43.14%)	9 (34.62%)	
**AJCC stage**			0.559
IA	3 (5.88%)	0 (0.00%)	
IB	4 (7.84%)	1 (3.85%)	
IIA	9 (17.65%)	4 (15.38%)	
IIB	34 (66.67%)	21 (80.77%)	
III	1 (1.96%)	0 (0.00%)	
**Residual tumor**			0.017
R0	35 (68.63%)	9 (34.62%)	
R1	15 (29.41%)	16 (61.54%)	
R2	1 (1.96%)	1 (3.85%)	
**Site**			0.497
Head of Pancreas	41 (80.39%)	24 (92.31%)	
Body of Pancreas	3 (5.88%)	1 (3.85%)	
Tail of Pancreas	4 (7.84%)	1 (3.85%)	
Others	3 (5.88%)	0 (0.00%)	
**Initial pathologic diagnosis method**			0.887
Tumor resection	31 (60.78%)	17 (65.38%)	
Tissue Biopsy	13 (25.49%)	5 (19.23%)	
Cytology (e.g. Peritoneal or pleural fluid)	5 (9.80%)	3 (11.54%)	
Fine Needle Aspiration Biopsy	1 (1.96%)	1 (3.85%)	
Not available	1 (1.96%)	0 (0.00%)	
**Surgical treatment**			0.761
Whipple	41 (80.39%)	24 (92.31%)	
Distal Pancreatectomy	6 (11.76%)	2 (7.69%)	
Distal Pancreatectomy & laporoscopy followed by Hand-assisted and Splenectomy	1 (1.96%)	0 (0.00%)	
Subtotal pancreatectomy and splenectomy and cholecystectomy	1 (1.96%)	0 (0.00%)	
Total Pancreatectomy	1 (1.96%)	0 (0.00%)	
Endoscopic Retrograde Cholangiopancreaticography	1 (1.96%)	0 (0.00%)	
**History of chemotherapy**			0.057
No	11 (21.57%)	11 (42.31%)	
Yes	40 (78.43%)	15 (57.69%)	
**History of radiation therapy**			0.039
No	34 (66.67%)	23 (88.46%)	
Yes	17 (33.33%)	3 (11.54%)	
**History of targeted molecular therapy**			< 0.001
No	10 (19.61%)	15 (57.69%)	
Yes	41 (80.39%)	11 (42.31%)	
**Tobacco smoking history**			0.550
Lifelong Non-smoker	18 (35.29%)	12 (46.15%)	
Current smoker	7 (13.73%)	6 (23.08%)	
Current reformed smoker for > 15 years	13 (25.49%)	4 (15.38%)	
Current reformed smoker for ≤ 15 years	9 (17.65%)	3 (11.54%)	
Current reformed smoker, duration not specified	4 (7.84%)	1 (3.85%)	
**Alcohol drinking history**			0.679
No	14 (27.45%)	6 (23.08%)	
Yes	37 (72.55%)	20 (76.92%)	
**History of chronic pancreatitis**			0.655
No	45 (88.24%)	22 (84.62%)	
Yes	6 (11.76%)	4 (15.38%)	
**History of diabetes**			0.679
No	37 (72.55%)	20 (76.92%)	
Yes	14 (27.45%)	6 (23.08%)	
**History of Prior Malignancy**			0.981
No	47 (92.16%)	24 (92.31%)	
Yes	4 (7.84%)	2 (7.69%)	

PDAC patients from the TCGA-PAAD dataset without complete clinical information were excluded.

**Table 4:** Unadjusted univariate Cox analysis

Exposure	Statistics	Overall Survival
Risk Score	1.80 ± 0.31	6.11 (2.01, 18.59) 0.0014
Age	63.84 ± 11.68	1.02 (0.99, 1.05) 0.1665
**Sex**		
Male	44 (57.14%)	1
Female	33 (42.86%)	1.26 (0.71, 2.24) 0.4278
**Size (cm)**	3.70 ± 1.50	1.22 (1.01, 1.47) 0.0422
***KRAS* mutation**		
Wildtype	10 (12.99%)	1
Mutant	67 (87.01%)	1.21 (0.51, 2.86) 0.6593
***TP53* mutation**		
Wildtype	25 (32.47%)	1
Mutant	52 (67.53%)	1.27 (0.68, 2.37) 0.4612
***CDKN2A* mutation**		
Wildtype	44 (57.14%)	1
Mutant	33 (42.86%)	1.87 (1.04, 3.36) 0.0362
***SMAD4* mutation**		
Wildtype	56 (72.73%)	1
Mutant	21 (27.27%)	0.72 (0.37, 1.39) 0.3267
***BRCA1* mutation**		
Wildtype	73 (94.81%)	1
Mutant	4 (5.19%)	4.06 (1.43, 11.53) 0.0086
***BRCA2* mutation**		
Wildtype	75 (97.40%)	1
Mutant	2 (2.60%)	1.87 (0.45, 7.80) 0.3892
**Site**		
Head of Pancreas	65 (84.42%)	1
Body and tail of Pancreas and others	12 (15.58%)	0.39 (0.14, 1.10) 0.0744
**Subtype**		
Pancreas-Adenocarcinoma Ductal Type	69 (89.61%)	1
Pancreas-Adenocarcinoma-Other Subtype	8 (10.39%)	0.60 (0.22, 1.68) 0.3336
**Grade**		
G1 and G2	50 (64.94%)	1
G3 and G4	27 (35.06%)	1.41 (0.79, 2.53) 0.2487
**T**		
T1 and T2	11 (14.29%)	1
T3 and T4	66 (85.71%)	2.45 (0.87, 6.88) 0.0887
**N**		
N0	22 (28.57%)	1
N1	55 (71.43%)	2.33 (1.12, 4.85) 0.0231
**AJCC stage**		
I	8 (10.39%)	1
II and III	69 (89.61%)	1.63 (0.58, 4.57) 0.3547
**Residual tumor**		
R0	44 (57.14%)	1
R1	31 (40.26%)	2.14 (1.17, 3.92) 0.0132
R2	2 (2.60%)	1.92 (0.25, 14.58) 0.5270
**Surgical treatment**		
Whipple	65 (84.42%)	1
Distal Pancreatectomy	9 (11.69%)	0.55 (0.20, 1.54) 0.2520
Others	3 (3.90%)	0.00 (0.00, Inf) 0.9971
**History of radiation therapy**		
No	57 (74.03%)	1
Yes	20 (25.97%)	0.28 (0.12, 0.66) 0.0035
**History of targeted molecular therapy**		
No	25 (32.47%)	1
Yes	52 (67.53%)	0.18 (0.10, 0.33) < 0.0001
**History of chemotherapy**		
No	22 (28.57%)	1
Yes	55 (71.43%)	0.36 (0.20, 0.66) 0.0008
**Tobacco smoking history**		
Lifelong Non-smoker	30 (38.96%)	1
Current or former smoker	47 (61.04%)	0.80 (0.44, 1.44) 0.4526
**Alcohol drinking history**		
No	20 (25.97%)	1
Yes	57 (74.03%)	1.45 (0.74, 2.86) 0.2782
**History of chronic pancreatitis**		
No	67 (87.01%)	1
Yes	10 (12.99%)	0.70 (0.30, 1.66) 0.4209
**History of diabetes**		
No	57 (74.03%)	1
Yes	20 (25.97%)	0.51 (0.24, 1.10) 0.0849
**History of prior malignancy**		
No	71 (92.21%)	1
Yes	6 (7.79%)	1.59 (0.56, 4.52) 0.3848

**Table 5:** Multivariate Cox regression analysis

Exposure	Non-adjusted	Adjust I	Adjust II	Adjust III
Risk Score	6.11 (2.01, 18.59) 0.0014	7.66 (2.28, 25.70) 0.0010	7.66 (2.28, 25.70) 0.0010	7.50 (1.93, 29.18) 0.0037
Age	1.02 (0.99, 1.05) 0.1665	1.02 (0.99, 1.05) 0.2208	1.03 (1.00, 1.06) 0.0806	1.02 (0.99, 1.06) 0.1834
**Sex**				
Male	1	1	1	NA
Female	1.26 (0.71, 2.24) 0.4278	1.22 (0.68, 2.17) 0.5065	1.23 (0.69, 2.19) 0.4921	NA
Size (cm)	1.22 (1.01, 1.47) 0.0422	1.26 (1.02, 1.55) 0.0318	1.24 (1.00, 1.53) 0.0450	1.23 (0.97, 1.57) 0.0828
***KRAS* mutation**				
Wildtype	1	1	1	NA
Mutant	1.21 (0.51, 2.86) 0.6593	1.19 (0.50, 2.84) 0.6990	0.95 (0.39, 2.27) 0.9016	NA
***TP53* mutation**				
Wildtype	1	1	1	NA
Mutant	1.27 (0.68, 2.37) 0.4612	1.40 (0.73, 2.66) 0.3086	1.10 (0.55, 2.20) 0.7773	NA
***CDKN2A* mutation**				
Wildtype	1	1	1	1
Mutant	1.87 (1.04, 3.36) 0.0362	2.02 (1.07, 3.80) 0.0290	1.48 (0.75, 2.93) 0.2546	0.79 (0.37, 1.66) 0.5279
***SMAD4* mutation**				
Wildtype	1	1	1	NA
Mutant	0.72 (0.37, 1.39) 0.3267	0.75 (0.39, 1.46) 0.4045	0.85 (0.43, 1.68) 0.6420	NA
***BRCA1* mutation**				
Wildtype	1	1	1	1
Mutant	4.06 (1.43, 11.53) 0.0086	3.72 (1.26, 10.97) 0.0171	2.68 (0.89, 8.07) 0.0807	2.31 (0.61, 8.72) 0.2188
***BRCA2* mutation**				
Wildtype	1	1	1	NA
Mutant	1.87 (0.45, 7.80) 0.3892	1.94 (0.44, 8.65) 0.3829	2.04 (0.46, 9.10) 0.3476	NA
**Site**				
Head of Pancreas	1	1	1	1
Body and tail of Pancreas and others	0.39 (0.14, 1.10) 0.0744	0.42 (0.15, 1.19) 0.1019	0.41 (0.14, 1.20) 0.1052	0.54 (0.15, 1.95) 0.3459
**Subtype**				
Pancreas-Adenocarcinoma Ductal Type	1	1	1	NA
Pancreas-Adenocarcinoma-Other Subtype	0.60 (0.22, 1.68) 0.3336	0.67 (0.24, 1.89) 0.4447	0.76 (0.27, 2.20) 0.6180	NA
**Grade**				
G1 and G2	1	1	1	1
G3 and G4	1.41 (0.79, 2.53) 0.2487	1.33 (0.73, 2.41) 0.3471	1.10 (0.60, 2.01) 0.7582	1.12 (0.55, 2.28) 0.7447
**T**				
T1 and T2	1	1	1	1
T3 and T4	2.45 (0.87, 6.88) 0.0887	inf. (0.00, Inf) 0.9958	inf. (0.00, Inf) 0.9960	1.75 (0.47, 6.47) 0.4049
**N**				
N0	1	1	1	1
N1	2.33 (1.12, 4.85) 0.0231	2.48 (0.96, 6.45) 0.0620	2.26 (0.87, 5.86) 0.0926	1.61 (0.63, 4.09) 0.3195
**AJCC stage**				
I	1	1	1	NA
II and III	1.63 (0.58, 4.57) 0.3547	1.53 (0.54, 4.32) 0.4194	1.43 (0.51, 4.02) 0.4991	NA
**Residual tumor**				
R0	1	1	1	1
R1	2.14 (1.17, 3.92) 0.0132	2.27 (1.21, 4.26) 0.0108	2.00 (1.06, 3.78) 0.0331	1.25 (0.55, 2.83) 0.5878
R2	1.92 (0.25, 14.58) 0.5270	1.92 (0.24, 15.22) 0.5387	2.61 (0.32, 21.37) 0.3718	4.69 (0.45, 48.71) 0.1958
**Surgical treatment**				
Whipple	1	1	1	NA
Distal Pancreatectomy	0.55 (0.20, 1.54) 0.2520	0.60 (0.21, 1.69) 0.3308	0.60 (0.21, 1.72) 0.3419	NA
Others	0.00 (0.00, Inf) 0.9971	0.00 (0.00, Inf) 0.9971	0.00 (0.00, Inf) 0.9972	NA
**History of radiation therapy**				
No	1	1	1	1
Yes	0.28 (0.12, 0.66) 0.0035	0.27 (0.11, 0.65) 0.0034	0.27 (0.11, 0.66) 0.0042	0.43 (0.16, 1.19) 0.1059
**History of targeted molecular therapy**				
No	1	1	1	1
Yes	0.18 (0.10, 0.33) < 0.0001	0.16 (0.08, 0.30) < 0.0001	0.14 (0.07, 0.28) < 0.0001	0.24 (0.07, 0.84) 0.0260
**History of chemotherapy**				
No	1	1	1	1
Yes	0.36 (0.20, 0.66) 0.0008	0.26 (0.14, 0.49) < 0.0001	0.22 (0.11, 0.43) < 0.0001	0.48 (0.13, 1.79) 0.2740
**Tobacco smoking history**				
Lifelong non-smoker	1	1	1	NA
Current or former smoker	0.80 (0.44, 1.44) 0.4526	0.81 (0.45, 1.47) 0.4970	0.93 (0.51, 1.70) 0.8087	NA
**Alcohol drinking history**				
No	1	1	1	NA
Yes	1.45 (0.74, 2.86) 0.2782	1.35 (0.66, 2.76) 0.4175	1.08 (0.51, 2.25) 0.8467	NA
**History of chronic pancreatitis**				
No	1	1	1	NA
Yes	0.70 (0.30, 1.66) 0.4209	0.77 (0.31, 1.89) 0.5656	0.49 (0.20, 1.24) 0.1319	NA
**History of diabetes**				
No	1	1	1	1
Yes	0.51 (0.24, 1.10) 0.0849	0.54 (0.24, 1.19) 0.1264	0.62 (0.28, 1.38) 0.2417	0.32 (0.13, 0.79) 0.0141
**History of prior malignancy**				
No	1	1	1	NA
Yes	1.59 (0.56, 4.52) 0.3848	1.38 (0.48, 4.02) 0.5503	1.03 (0.35, 3.02) 0.9599	NA

Adjust I model adjust for: Age, Sex, and AJCC Stage; Adjust II model adjust for: Age, Sex, AJCC Stage, and Risk Score; Adjust III model adjust for parameters with *p* < 0.25 based on univariate analysis.

## Abbreviations

AUC: area under the curve
CI: confidence interval
DEGs: differentially expressed genes
DE-MTGs: differentially expressed MTGs
ECM: extracellular matrix
EMT: epithelial-to-mesenchymal
GSEA: gene set enrichment analysis
HR: hazard ratio
MTGs: metastasis-related genes
PDAC: pancreatic ductal adenocarcinoma
ROC: receiver operating characteristic
TME: tumor microenvironment
TPM: transcripts per million
Tregs: regulatory T cells
