# Supplementary material for: Development and validation of a metastasis-related Gene Signature for predicting the Overall Survival in patients with Pancreatic Ductal Adenocarcinoma
Source: J Cancer. 2020 Aug 29;11(21):6299–318. doi: 10.7150/jca.47629 (PMC7532518; doi:10.7150/jca.47629)
Supplement: Supplementary file 1 — Supplementary figures and tables. [file jcav11p6299s1.pdf]

Table S1. DEGs identified in PDAC

Gene	logFC	P Value	FDR	regulated
LAMC2	2.650	1.51583E-33	2.28981E-29	Up-Regulated
S100P	4.297	3.48635E-33	2.63324E-29	Up-Regulated
KRT19	3.527	7.83878E-33	3.94709E-29	Up-Regulated
GPRC5A	2.936	2.42274E-32	7.59769E-29	Up-Regulated
ITGA2	2.048	2.51479E-32	7.59769E-29	Up-Regulated
SLPI	2.634	1.12073E-31	2.82161E-28	Up-Regulated
ANXA2	1.298	2.99506E-31	6.46334E-28	Up-Regulated
INHBA	3.142	2.69629E-30	5.09126E-27	Up-Regulated
GJB2	3.389	3.20932E-30	5.38667E-27	Up-Regulated
FN1	2.787	1.02154E-29	1.54314E-26	Up-Regulated
SLC6A14	3.817	1.86355E-29	2.55916E-26	Up-Regulated
KRT7	2.681	2.05178E-29	2.58285E-26	Up-Regulated
SULF1	3.126	3.60334E-29	4.18708E-26	Up-Regulated
SFN	2.944	6.54358E-29	7.06052E-26	Up-Regulated
AHNAK2	2.281	8.47941E-29	8.53934E-26	Up-Regulated
TNFRSF21	1.607	2.14461E-28	2.02478E-25	Up-Regulated
COL10A1	3.480	2.76465E-28	2.45664E-25	Up-Regulated
LAMB3	2.223	3.55549E-28	2.98384E-25	Up-Regulated
NQO1	2.573	6.26771E-28	4.98316E-25	Up-Regulated
ANO1	1.826	6.90453E-28	5.21499E-25	Up-Regulated
SERPINB5	2.598	1.64594E-27	1.18398E-24	Up-Regulated
S100A11	1.839	1.89543E-27	1.30147E-24	Up-Regulated
IGFBP3	2.459	2.09147E-27	1.37364E-24	Up-Regulated
S100A10	1.439	3.54929E-27	2.23398E-24	Up-Regulated
COL11A1	3.605	4.55117E-27	2.75E-24	Up-Regulated
SDR16C5	3.022	5.35834E-27	3.11319E-24	Up-Regulated
MBOAT2	1.350	5.73091E-27	3.20634E-24	Up-Regulated
TMPRSS4	2.301	7.61661E-27	4.10916E-24	Up-Regulated
S100A16	1.380	1.03867E-26	5.41041E-24	Up-Regulated
DCBLD1	1.237	1.46446E-26	7.37403E-24	Up-Regulated
ITGB1BP1	1.161	1.60406E-26	7.81644E-24	Up-Regulated
NHS	1.493	2.08194E-26	9.53022E-24	Up-Regulated
BIK	1.840	3.08514E-26	1.37071E-23	Up-Regulated
CTHRC1	3.221	3.35714E-26	1.44894E-23	Up-Regulated
S100A6	2.392	3.48733E-26	1.46332E-23	Up-Regulated
CAPG	1.813	7.40686E-26	3.024E-23	Up-Regulated
GJB3	1.206	7.8409E-26	3.11696E-23	Up-Regulated
SDC1	1.422	8.08522E-26	3.13167E-23	Up-Regulated
KDM5B	1.053	8.31294E-26	3.13938E-23	Up-Regulated
COL1A2	3.012	9.47339E-26	3.49037E-23	Up-Regulated
NOX4	1.997	1.15155E-25	4.14173E-23	Up-Regulated
HOXB7	1.497	2.20995E-25	7.76362E-23	Up-Regulated
TPBG	1.330	2.32188E-25	7.97144E-23	Up-Regulated
C15orf48	2.588	2.60955E-25	8.75998E-23	Up-Regulated
FHL2	1.802	3.28089E-25	1.07741E-22	Up-Regulated
ARNTL2	1.571	5.15371E-25	1.65642E-22	Up-Regulated
MXRA5	1.976	5.44454E-25	1.71344E-22	Up-Regulated
HOXB3	1.354	6.25057E-25	1.92696E-22	Up-Regulated
TSPAN1	2.076	7.43038E-25	2.24487E-22	Up-Regulated
COL5A2	2.580	8.67099E-25	2.54123E-22	Up-Regulated

MYOF	1.737	8.74778E-25	2.54123E-22	Up-Regulated
CTSE	3.136	1.22047E-24	3.47858E-22	Up-Regulated
ADAMTS12	1.508	1.38002E-24	3.86047E-22	Up-Regulated
SHISA2	2.153	1.94594E-24	5.34461E-22	Up-Regulated
THBS2	2.681	2.2053E-24	5.94881E-22	Up-Regulated
SOX4	1.139	2.48598E-24	6.5374E-22	Up-Regulated
EFNA5	1.332	2.51006E-24	6.5374E-22	Up-Regulated
RTP4	1.293	3.02265E-24	7.53528E-22	Up-Regulated
POSTN	3.309	3.04284E-24	7.53528E-22	Up-Regulated
IFI27	2.176	3.84702E-24	9.37307E-22	Up-Regulated
NET1	1.170	4.25854E-24	1.0211E-21	Up-Regulated
MSLN	2.889	4.34982E-24	1.02669E-21	Up-Regulated
AMIGO2	1.687	4.55706E-24	1.05906E-21	Up-Regulated
FXYD3	1.948	4.89832E-24	1.12112E-21	Up-Regulated
DKK1	2.605	6.63757E-24	1.49652E-21	Up-Regulated
LY6E	1.441	6.99354E-24	1.55359E-21	Up-Regulated
OSBPL3	1.600	7.66898E-24	1.67895E-21	Up-Regulated
KLK10	2.285	8.89584E-24	1.91972E-21	Up-Regulated
CEACAM6	3.786	9.25046E-24	1.96813E-21	Up-Regulated
CD9	1.272	1.16825E-23	2.44678E-21	Up-Regulated
STEAP1	1.770	1.18241E-23	2.44678E-21	Up-Regulated
LGALS3	1.412	1.35063E-23	2.75712E-21	Up-Regulated
ITGA3	1.502	1.63418E-23	3.29145E-21	Up-Regulated
NFE2L3	1.417	1.83919E-23	3.65563E-21	Up-Regulated
SMAGP	1.293	2.08687E-23	4.09407E-21	Up-Regulated
SESTD1	1.113	2.63383E-23	5.10084E-21	Up-Regulated
HN1	1.578	4.4819E-23	8.57008E-21	Up-Regulated
CKLF	1.335	6.04684E-23	1.1277E-20	Up-Regulated
PKM	1.358	7.1455E-23	1.31634E-20	Up-Regulated
FERMT1	1.786	7.94031E-23	1.44514E-20	Up-Regulated
TIMP1	2.013	1.02281E-22	1.83936E-20	Up-Regulated
CEACAM5	4.088	1.13206E-22	2.01187E-20	Up-Regulated
FGD6	1.354	1.37605E-22	2.41705E-20	Up-Regulated
MLPH	1.898	1.4598E-22	2.53468E-20	Up-Regulated
CST1	2.898	1.50379E-22	2.54506E-20	Up-Regulated
ACVR1	1.185	1.52125E-22	2.54506E-20	Up-Regulated
CSTB	1.250	1.52762E-22	2.54506E-20	Up-Regulated
NMU	2.293	1.53317E-22	2.54506E-20	Up-Regulated
CDH3	1.672	1.782E-22	2.89738E-20	Up-Regulated
KRT17	2.403	1.78377E-22	2.89738E-20	Up-Regulated
CD55	1.902	1.90417E-22	3.06005E-20	Up-Regulated
HK2	1.817	2.12681E-22	3.38186E-20	Up-Regulated
VCAN	2.583	2.62213E-22	4.12603E-20	Up-Regulated
PFKP	1.561	3.01228E-22	4.69108E-20	Up-Regulated
PPAPDC1A	1.987	3.18243E-22	4.90549E-20	Up-Regulated
COL5A1	2.057	3.74844E-22	5.71959E-20	Up-Regulated
CAPN8	2.587	4.6872E-22	7.01038E-20	Up-Regulated
ISG15	1.789	4.9303E-22	7.30167E-20	Up-Regulated
FAM19A5	1.674	5.60615E-22	8.22199E-20	Up-Regulated
LCN2	3.038	5.72284E-22	8.31242E-20	Up-Regulated
ANLN	1.890	6.21111E-22	8.93572E-20	Up-Regulated
FAT1	1.356	6.38669E-22	9.10163E-20	Up-Regulated

SDC4	1.179	6.95445E-22	9.81813E-20	Up-Regulated
LGALS3BP	1.471	9.72522E-22	1.33554E-19	Up-Regulated
SAMD9	1.562	9.8573E-22	1.34148E-19	Up-Regulated
KCNN4	1.514	1.09322E-21	1.47448E-19	Up-Regulated
MGLL	1.511	1.19484E-21	1.5695E-19	Up-Regulated
MARCKSL1	1.422	1.45721E-21	1.86548E-19	Up-Regulated
OSBPL10	1.299	1.50942E-21	1.91608E-19	Up-Regulated
SLC2A1	1.558	1.6045E-21	2.0198E-19	Up-Regulated
C19orf33	2.277	1.65881E-21	2.05393E-19	Up-Regulated
EFNB2	1.717	2.37555E-21	2.91748E-19	Up-Regulated
SMYD3	1.028	2.67288E-21	3.20448E-19	Up-Regulated
TMC5	2.298	3.15814E-21	3.7271E-19	Up-Regulated
KCNK1	1.745	4.20417E-21	4.92312E-19	Up-Regulated
ECT2	1.224	4.30037E-21	4.99703E-19	Up-Regulated
MAP4K4	1.029	5.36191E-21	6.13614E-19	Up-Regulated
AHR	1.388	5.47428E-21	6.18614E-19	Up-Regulated
TSPO	1.081	5.48751E-21	6.18614E-19	Up-Regulated
PMEPA1	1.536	5.77557E-21	6.45603E-19	Up-Regulated
TM4SF1	1.513	5.81239E-21	6.45603E-19	Up-Regulated
SYTL2	1.578	5.98494E-21	6.59917E-19	Up-Regulated
COL3A1	2.181	6.32843E-21	6.92733E-19	Up-Regulated
C1orf106	1.908	6.64896E-21	7.22584E-19	Up-Regulated
NBL1	1.346	8.69175E-21	9.34735E-19	Up-Regulated
MTMR11	1.381	8.72486E-21	9.34735E-19	Up-Regulated
FZD2	1.138	9.1288E-21	9.71124E-19	Up-Regulated
BPGM	1.144	1.14898E-20	1.197E-18	Up-Regulated
ABHD17C	1.880	1.40076E-20	1.43944E-18	Up-Regulated
HOXC6	2.015	1.69996E-20	1.73511E-18	Up-Regulated
NTM	1.360	2.01553E-20	2.01235E-18	Up-Regulated
SFTA2	2.327	2.40885E-20	2.3783E-18	Up-Regulated
SULF2	1.863	2.44863E-20	2.40188E-18	Up-Regulated
MPZL2	1.184	2.48877E-20	2.42551E-18	Up-Regulated
MST1R	1.477	2.56504E-20	2.48382E-18	Up-Regulated
SPON2	1.328	2.7176E-20	2.61478E-18	Up-Regulated
LEMD1	1.882	3.5026E-20	3.34875E-18	Up-Regulated
JUP	1.270	3.7489E-20	3.56169E-18	Up-Regulated
TMPRSS3	1.573	3.77929E-20	3.56812E-18	Up-Regulated
AGR2	2.754	3.91238E-20	3.67083E-18	Up-Regulated
INPP4B	1.572	4.11524E-20	3.81379E-18	Up-Regulated
APOL1	1.015	4.44019E-20	4.08985E-18	Up-Regulated
LTBP1	1.244	4.74966E-20	4.34839E-18	Up-Regulated
PCDH7	1.370	5.14774E-20	4.68444E-18	Up-Regulated
XAF1	1.794	6.10238E-20	5.39079E-18	Up-Regulated
PLAUR	1.502	6.62283E-20	5.81654E-18	Up-Regulated
KRT23	2.223	7.04892E-20	6.15497E-18	Up-Regulated
KLF5	2.085	7.29281E-20	6.33133E-18	Up-Regulated
CAP1	1.128	7.81989E-20	6.75013E-18	Up-Regulated
RAI14	1.204	8.56561E-20	7.35182E-18	Up-Regulated
SRPX2	1.435	9.36647E-20	7.94887E-18	Up-Regulated
PLAT	2.093	9.4624E-20	7.98542E-18	Up-Regulated
NPC1	1.184	9.79753E-20	8.2223E-18	Up-Regulated
EPHX4	1.131	1.02922E-19	8.54249E-18	Up-Regulated

TRIM59	1.357	1.03671E-19	8.55767E-18	Up-Regulated
RRAS	1.120	1.06038E-19	8.70553E-18	Up-Regulated
CST4	1.359	1.53495E-19	1.22682E-17	Up-Regulated
ANKRD22	1.468	1.70526E-19	1.35577E-17	Up-Regulated
ITPR3	1.044	1.74898E-19	1.38325E-17	Up-Regulated
GABRP	3.114	1.91682E-19	1.50809E-17	Up-Regulated
DKK3	1.346	1.94999E-19	1.52625E-17	Up-Regulated
SPARC	1.835	2.12078E-19	1.62573E-17	Up-Regulated
B4GALT5	1.059	2.21415E-19	1.68075E-17	Up-Regulated
ANXA3	1.651	2.34191E-19	1.76884E-17	Up-Regulated
FXYD5	1.535	2.47063E-19	1.84759E-17	Up-Regulated
RAB31	1.866	2.73711E-19	2.03678E-17	Up-Regulated
PLAU	1.695	2.75283E-19	2.03844E-17	Up-Regulated
BHLHE40	1.302	2.80005E-19	2.0633E-17	Up-Regulated
COL6A3	1.795	2.8611E-19	2.08791E-17	Up-Regulated
FOXQ1	2.130	3.01042E-19	2.18632E-17	Up-Regulated
MCU	1.158	3.13963E-19	2.26924E-17	Up-Regulated
KYNU	1.413	3.85769E-19	2.76181E-17	Up-Regulated
TNFSF4	1.336	4.01772E-19	2.86281E-17	Up-Regulated
OLFML2B	1.926	4.0393E-19	2.86468E-17	Up-Regulated
PRKCI	1.072	4.09814E-19	2.87937E-17	Up-Regulated
SPOCK1	1.585	4.15053E-19	2.90268E-17	Up-Regulated
RACGAP1	1.419	4.56045E-19	3.1601E-17	Up-Regulated
TMSB10	1.205	5.59327E-19	3.84054E-17	Up-Regulated
ENO2	1.475	6.4911E-19	4.43686E-17	Up-Regulated
NMI	1.158	7.3301E-19	4.98777E-17	Up-Regulated
GALNT5	1.265	7.44558E-19	5.04363E-17	Up-Regulated
FAP	2.128	7.56228E-19	5.09981E-17	Up-Regulated
GPX8	1.353	8.32964E-19	5.52453E-17	Up-Regulated
SGPP2	1.936	9.02938E-19	5.95624E-17	Up-Regulated
STYK1	1.455	1.03591E-18	6.80369E-17	Up-Regulated
DDX60	1.454	1.24758E-18	8.11378E-17	Up-Regulated
SGIP1	1.100	1.32494E-18	8.51685E-17	Up-Regulated
SLC24A3	1.224	1.33326E-18	8.53401E-17	Up-Regulated
EFNA1	1.186	1.56813E-18	9.78849E-17	Up-Regulated
BACE2	1.036	1.74271E-18	1.08335E-16	Up-Regulated
COL4A1	1.488	1.77377E-18	1.09814E-16	Up-Regulated
PALLD	1.644	1.92738E-18	1.18354E-16	Up-Regulated
VCL	1.026	1.94015E-18	1.18656E-16	Up-Regulated
MMP28	1.264	1.97516E-18	1.20262E-16	Up-Regulated
IFI44	1.484	1.98233E-18	1.20262E-16	Up-Regulated
PNMA1	1.363	2.0156E-18	1.21791E-16	Up-Regulated
SEMA3C	1.930	2.06285E-18	1.23656E-16	Up-Regulated
S100A4	1.891	2.18637E-18	1.30029E-16	Up-Regulated
STIL	1.006	2.22737E-18	1.31948E-16	Up-Regulated
PRC1	1.267	2.37343E-18	1.39506E-16	Up-Regulated
ACTN1	1.056	2.64075E-18	1.54617E-16	Up-Regulated
TPM4	1.102	2.73351E-18	1.58707E-16	Up-Regulated
ZNF532	1.159	2.74213E-18	1.58707E-16	Up-Regulated
AEBP1	1.840	3.13313E-18	1.80645E-16	Up-Regulated
OAS3	1.022	3.29969E-18	1.89525E-16	Up-Regulated
PLEK2	1.196	3.32845E-18	1.90261E-16	Up-Regulated

C1GALT1	1.198	3.55139E-18	2.00926E-16	Up-Regulated
KDELC1	1.285	4.06469E-18	2.27412E-16	Up-Regulated
ECM1	1.293	4.17247E-18	2.30877E-16	Up-Regulated
LIPH	1.393	4.28271E-18	2.36112E-16	Up-Regulated
NUAK1	1.308	4.40085E-18	2.39997E-16	Up-Regulated
LIMS1	1.056	4.94723E-18	2.6501E-16	Up-Regulated
EPS8	1.214	5.0295E-18	2.67995E-16	Up-Regulated
STEAP2	1.081	5.99628E-18	3.10845E-16	Up-Regulated
CEP55	1.535	6.00866E-18	3.10845E-16	Up-Regulated
COL12A1	1.991	6.12271E-18	3.14778E-16	Up-Regulated
TRIB2	1.280	6.12635E-18	3.14778E-16	Up-Regulated
FNDC1	2.137	6.18868E-18	3.16902E-16	Up-Regulated
PPL	1.259	6.2734E-18	3.20155E-16	Up-Regulated
WISP1	1.376	6.3148E-18	3.21183E-16	Up-Regulated
SLC16A4	1.625	7.102E-18	3.58806E-16	Up-Regulated
PI3	1.712	7.21087E-18	3.63091E-16	Up-Regulated
GPX2	2.203	7.74425E-18	3.87366E-16	Up-Regulated
SPAG1	1.290	8.67938E-18	4.29871E-16	Up-Regulated
COL4A2	1.442	8.86198E-18	4.37481E-16	Up-Regulated
NCEH1	1.420	9.50648E-18	4.64741E-16	Up-Regulated
CRIP1	1.967	9.68521E-18	4.71951E-16	Up-Regulated
SMS	1.119	1.02033E-17	4.95599E-16	Up-Regulated
LOXL1	1.559	1.03237E-17	4.96659E-16	Up-Regulated
AK4	1.202	1.20991E-17	5.75807E-16	Up-Regulated
OAS1	1.373	1.2353E-17	5.83603E-16	Up-Regulated
WNT5A	1.291	1.23628E-17	5.83603E-16	Up-Regulated
TGFBI	1.478	1.26796E-17	5.94838E-16	Up-Regulated
COMP	2.410	1.34936E-17	6.31068E-16	Up-Regulated
PON2	1.153	1.3991E-17	6.47403E-16	Up-Regulated
MELK	1.344	1.55736E-17	7.10741E-16	Up-Regulated
BGN	1.586	1.59439E-17	7.25449E-16	Up-Regulated
PRRG1	1.113	1.62923E-17	7.39075E-16	Up-Regulated
TMEM158	1.604	2.11202E-17	9.35939E-16	Up-Regulated
MMP7	2.582	2.11277E-17	9.35939E-16	Up-Regulated
MFAP2	1.436	2.25372E-17	9.9546E-16	Up-Regulated
MX1	1.383	2.36066E-17	1.03362E-15	Up-Regulated
LDHA	1.038	2.41231E-17	1.05015E-15	Up-Regulated
RSAD2	1.336	2.68468E-17	1.16203E-15	Up-Regulated
TCN1	2.807	2.69243E-17	1.16205E-15	Up-Regulated
EPPK1	1.470	2.78442E-17	1.19833E-15	Up-Regulated
TMEM200A	1.866	3.09337E-17	1.32375E-15	Up-Regulated
MICALL2	1.026	3.11866E-17	1.3308E-15	Up-Regulated
GCNT3	2.276	3.24691E-17	1.37775E-15	Up-Regulated
MAL2	1.980	3.71409E-17	1.56718E-15	Up-Regulated
SQLE	1.101	3.96162E-17	1.65316E-15	Up-Regulated
TIGD1	1.022	4.25751E-17	1.76202E-15	Up-Regulated
TOP2A	2.074	5.65143E-17	2.29491E-15	Up-Regulated
CORO1C	1.165	5.99609E-17	2.41539E-15	Up-Regulated
MXRA8	1.046	6.28608E-17	2.52E-15	Up-Regulated
THY1	1.339	6.28914E-17	2.52E-15	Up-Regulated
MMP11	1.411	6.40908E-17	2.5545E-15	Up-Regulated
CLIC1	1.023	6.58877E-17	2.61233E-15	Up-Regulated

CTSB	1.060	6.75911E-17	2.65894E-15	Up-Regulated
ZG16B	1.795	7.33611E-17	2.84152E-15	Up-Regulated
OLR1	2.105	8.03876E-17	3.07427E-15	Up-Regulated
SLC9B2	1.031	8.22732E-17	3.13843E-15	Up-Regulated
TFF1	3.213	8.83286E-17	3.32741E-15	Up-Regulated
MMP1	3.116	8.92279E-17	3.35293E-15	Up-Regulated
CDH11	1.338	1.03343E-16	3.81742E-15	Up-Regulated
CEP170	1.217	1.1912E-16	4.35695E-15	Up-Regulated
TGFB1I1	1.373	1.32404E-16	4.7849E-15	Up-Regulated
LY75	1.845	1.35219E-16	4.85183E-15	Up-Regulated
KIF14	1.181	1.48625E-16	5.30763E-15	Up-Regulated
GPR137B	1.372	1.5115E-16	5.3724E-15	Up-Regulated
KPNA2	1.169	1.53669E-16	5.44913E-15	Up-Regulated
ARL4C	1.320	1.60701E-16	5.67183E-15	Up-Regulated
OPN3	1.080	1.89396E-16	6.60744E-15	Up-Regulated
YWHAZ	1.015	1.9324E-16	6.72598E-15	Up-Regulated
SLC39A10	1.140	1.9613E-16	6.81089E-15	Up-Regulated
CMTM3	1.005	2.07359E-16	7.15152E-15	Up-Regulated
ATP2B4	1.122	2.09161E-16	7.18186E-15	Up-Regulated
CXCL5	2.714	2.22186E-16	7.57095E-15	Up-Regulated
SRGAP2B	1.127	2.25556E-16	7.65673E-15	Up-Regulated
MSMO1	1.215	2.28505E-16	7.72215E-15	Up-Regulated
HOXB2	1.124	2.31411E-16	7.80288E-15	Up-Regulated
NT5DC2	1.180	2.40667E-16	8.0216E-15	Up-Regulated
FKBP7	1.174	3.0456E-16	9.97977E-15	Up-Regulated
PMAIP1	1.513	3.05557E-16	9.99078E-15	Up-Regulated
CCL20	2.283	3.07064E-16	1.00184E-14	Up-Regulated
CKS2	1.816	3.2139E-16	1.04371E-14	Up-Regulated
CENPU	1.441	3.24358E-16	1.04618E-14	Up-Regulated
MALL	1.746	3.24811E-16	1.04618E-14	Up-Regulated
YWHAB	1.005	3.31212E-16	1.06227E-14	Up-Regulated
EDNRA	1.782	3.34831E-16	1.0716E-14	Up-Regulated
COL16A1	1.193	3.3594E-16	1.07288E-14	Up-Regulated
BEAN1	1.118	3.38959E-16	1.08024E-14	Up-Regulated
BAIAP2L1	1.167	3.46907E-16	1.09861E-14	Up-Regulated
PLXDC2	1.344	3.87247E-16	1.21364E-14	Up-Regulated
NDC80	1.431	4.33743E-16	1.34817E-14	Up-Regulated
LRRN1	1.442	4.37089E-16	1.35473E-14	Up-Regulated
CTSK	1.657	4.52339E-16	1.39166E-14	Up-Regulated
FAM83H	1.013	5.10153E-16	1.55593E-14	Up-Regulated
MX2	1.116	5.47241E-16	1.65664E-14	Up-Regulated
LGALS1	1.589	5.93138E-16	1.77777E-14	Up-Regulated
TRIM29	1.230	6.16585E-16	1.83727E-14	Up-Regulated
TXN	1.012	6.17749E-16	1.83727E-14	Up-Regulated
TNFAIP6	1.771	6.17858E-16	1.83727E-14	Up-Regulated
ANXA1	1.519	6.74158E-16	1.98129E-14	Up-Regulated
DACT1	1.484	6.99529E-16	2.04788E-14	Up-Regulated
CST2	1.255	7.16182E-16	2.08854E-14	Up-Regulated
HIST2H2BE	1.308	7.55608E-16	2.18663E-14	Up-Regulated
HK1	1.091	7.64906E-16	2.20509E-14	Up-Regulated
SLC44A4	1.777	9.6845E-16	2.72937E-14	Up-Regulated
F5	1.562	9.9096E-16	2.78242E-14	Up-Regulated

CD58	1.223	1.00986E-15	2.83022E-14	Up-Regulated
ADAM9	1.407	1.02266E-15	2.86081E-14	Up-Regulated
ERO1L	1.161	1.14599E-15	3.15325E-14	Up-Regulated
SH3KBP1	1.431	1.15764E-15	3.17951E-14	Up-Regulated
PANX1	1.020	1.18528E-15	3.24951E-14	Up-Regulated
GBP2	1.186	1.26479E-15	3.4425E-14	Up-Regulated
IFI6	1.684	1.34166E-15	3.6321E-14	Up-Regulated
PPFIBP1	1.043	1.38446E-15	3.72129E-14	Up-Regulated
VASP	1.011	1.48913E-15	3.95339E-14	Up-Regulated
FRMD6	1.312	1.65809E-15	4.37121E-14	Up-Regulated
SLC12A2	1.255	1.82547E-15	4.79574E-14	Up-Regulated
WNT2	1.150	1.86012E-15	4.84466E-14	Up-Regulated
SHROOM3	1.322	1.87381E-15	4.8719E-14	Up-Regulated
CXCL8	2.250	2.06172E-15	5.29666E-14	Up-Regulated
PCDHB10	1.085	2.13187E-15	5.4583E-14	Up-Regulated
BCL10	1.016	2.36054E-15	5.98897E-14	Up-Regulated
TNFAIP2	1.051	2.36292E-15	5.98897E-14	Up-Regulated
DPYSL3	1.416	2.3915E-15	6.04113E-14	Up-Regulated
GRHL1	1.044	2.77137E-15	6.89691E-14	Up-Regulated
DUOX2	2.509	2.8058E-15	6.94826E-14	Up-Regulated
KIF20A	1.135	2.87583E-15	7.11003E-14	Up-Regulated
IGF2BP2	1.138	3.10675E-15	7.59393E-14	Up-Regulated
SGK223	1.055	3.12857E-15	7.63493E-14	Up-Regulated
MIA	1.458	3.27425E-15	7.92641E-14	Up-Regulated
CORO2A	1.494	3.43756E-15	8.25561E-14	Up-Regulated
PXDN	1.352	3.93118E-15	9.39626E-14	Up-Regulated
HEPH	1.703	4.27913E-15	1.01001E-13	Up-Regulated
MDK	1.407	4.37578E-15	1.03121E-13	Up-Regulated
CAMK2N1	1.069	4.42386E-15	1.03447E-13	Up-Regulated
NUSAP1	1.264	4.86734E-15	1.13014E-13	Up-Regulated
TWIST1	1.532	4.87368E-15	1.13014E-13	Up-Regulated
ITGB6	1.716	5.35752E-15	1.23558E-13	Up-Regulated
MMP2	1.571	5.74944E-15	1.31393E-13	Up-Regulated
EPS8L1	1.096	5.78189E-15	1.31736E-13	Up-Regulated
SIPA1L2	1.028	6.21682E-15	1.4122E-13	Up-Regulated
EPHA2	1.110	7.12237E-15	1.60344E-13	Up-Regulated
EPSTI1	1.169	7.36535E-15	1.64344E-13	Up-Regulated
GOLM1	1.259	7.88583E-15	1.73903E-13	Up-Regulated
ZWINT	1.281	9.18079E-15	2.00412E-13	Up-Regulated
PCDHB14	1.128	9.9268E-15	2.14527E-13	Up-Regulated
PERP	1.298	9.99365E-15	2.15355E-13	Up-Regulated
ISLR	1.504	1.04452E-14	2.24126E-13	Up-Regulated
NPR3	1.491	1.05178E-14	2.25365E-13	Up-Regulated
PLOD2	1.068	1.07931E-14	2.30609E-13	Up-Regulated
DPCR1	2.320	1.13321E-14	2.41442E-13	Up-Regulated
SERPINH1	1.264	1.19385E-14	2.52228E-13	Up-Regulated
RARRES3	1.444	1.19768E-14	2.52453E-13	Up-Regulated
CENPW	1.121	1.21959E-14	2.5659E-13	Up-Regulated
DFNA5	1.012	1.31129E-14	2.73554E-13	Up-Regulated
HIST1H2BD	1.231	1.72907E-14	3.52964E-13	Up-Regulated
CENPK	1.466	1.8014E-14	3.67233E-13	Up-Regulated
S100A2	1.904	1.83378E-14	3.73331E-13	Up-Regulated

LAMB1	1.167	2.09315E-14	4.19908E-13	Up-Regulated
CCNB1	1.303	2.32886E-14	4.59657E-13	Up-Regulated
MN1	1.311	3.07782E-14	5.95407E-13	Up-Regulated
GREM1	2.467	3.09335E-14	5.96784E-13	Up-Regulated
RHPN2	1.428	3.12839E-14	6.02557E-13	Up-Regulated
ANXA10	2.381	3.2268E-14	6.1858E-13	Up-Regulated
CDCA7	1.553	3.34621E-14	6.3823E-13	Up-Regulated
F2R	1.090	3.49847E-14	6.64753E-13	Up-Regulated
MACC1	1.086	4.00144E-14	7.50878E-13	Up-Regulated
ITGB1	1.152	4.48617E-14	8.32531E-13	Up-Regulated
RARRES1	1.974	4.7124E-14	8.70238E-13	Up-Regulated
F3	1.054	4.80561E-14	8.84209E-13	Up-Regulated
EGLN3	1.353	4.87081E-14	8.91008E-13	Up-Regulated
TUFT1	1.089	5.38506E-14	9.67261E-13	Up-Regulated
PRR15	1.380	5.79512E-14	1.03232E-12	Up-Regulated
ARHGAP42	1.096	5.87083E-14	1.04458E-12	Up-Regulated
MATN3	1.764	5.94879E-14	1.05721E-12	Up-Regulated
VILL	1.380	6.14047E-14	1.08491E-12	Up-Regulated
PDGFRB	1.062	6.3265E-14	1.11255E-12	Up-Regulated
KIAA0101	1.130	6.56639E-14	1.15072E-12	Up-Regulated
BUB1B	1.243	6.57914E-14	1.15162E-12	Up-Regulated
FAM213B	1.012	7.32318E-14	1.26862E-12	Up-Regulated
SERPINA1	1.716	7.33583E-14	1.26936E-12	Up-Regulated
ZNF365	1.079	7.55467E-14	1.30424E-12	Up-Regulated
ASPN	1.422	8.02843E-14	1.37347E-12	Up-Regulated
GALNT4	1.152	8.29722E-14	1.41146E-12	Up-Regulated
S100A14	1.276	8.35303E-14	1.41936E-12	Up-Regulated
ASPHD2	1.013	8.36892E-14	1.42046E-12	Up-Regulated
IFI44L	1.567	8.58892E-14	1.45453E-12	Up-Regulated
LUM	1.234	8.7128E-14	1.47291E-12	Up-Regulated
IL18	1.261	8.87455E-14	1.49619E-12	Up-Regulated
C1orf116	1.277	8.89306E-14	1.49764E-12	Up-Regulated
DLGAP5	1.323	9.52373E-14	1.58792E-12	Up-Regulated
HIST1H2AC	1.115	9.75386E-14	1.62092E-12	Up-Regulated
NDNF	1.081	1.01828E-13	1.68664E-12	Up-Regulated
CLDN18	2.542	1.06104E-13	1.75554E-12	Up-Regulated
SUGCT	1.128	1.06298E-13	1.75683E-12	Up-Regulated
RAB34	1.109	1.09476E-13	1.80146E-12	Up-Regulated
LBH	1.101	1.1385E-13	1.86532E-12	Up-Regulated
COL1A1	1.345	1.15758E-13	1.89452E-12	Up-Regulated
KRT8	1.419	1.16049E-13	1.89723E-12	Up-Regulated
LIF	1.126	1.23973E-13	2.01585E-12	Up-Regulated
ARPC1B	1.233	1.60263E-13	2.535E-12	Up-Regulated
ST6GALNAC1	1.959	1.63561E-13	2.57638E-12	Up-Regulated
CLDN1	1.419	1.68431E-13	2.64207E-12	Up-Regulated
LRRC15	1.085	1.89821E-13	2.92305E-12	Up-Regulated
BCAS1	1.197	2.04639E-13	3.12249E-12	Up-Regulated
TIMP2	1.062	2.05992E-13	3.13998E-12	Up-Regulated
NEK2	1.128	2.21945E-13	3.36617E-12	Up-Regulated
GINS1	1.102	2.22607E-13	3.37281E-12	Up-Regulated
DTL	1.103	2.3368E-13	3.52644E-12	Up-Regulated
FBN1	1.455	2.46313E-13	3.70968E-12	Up-Regulated

VSIG2	1.163	2.47333E-13	3.72133E-12	Up-Regulated
MXD1	1.308	2.61033E-13	3.90026E-12	Up-Regulated
IGFL2	1.096	2.72275E-13	4.04821E-12	Up-Regulated
CSGALNACT2	1.054	2.74257E-13	4.07368E-12	Up-Regulated
LIMA1	1.291	2.92145E-13	4.29712E-12	Up-Regulated
CXCL3	1.599	3.02208E-13	4.4279E-12	Up-Regulated
FA2H	1.100	3.08748E-13	4.51059E-12	Up-Regulated
PTPRR	1.226	3.68252E-13	5.27281E-12	Up-Regulated
TRNP1	1.066	3.68829E-13	5.27607E-12	Up-Regulated
LAMP5	1.428	4.08386E-13	5.74938E-12	Up-Regulated
AOC1	2.178	4.14177E-13	5.82547E-12	Up-Regulated
ANXA8L1	1.445	4.81922E-13	6.70959E-12	Up-Regulated
CDC20	1.255	4.87416E-13	6.77983E-12	Up-Regulated
FAM83D	1.515	5.12608E-13	7.0846E-12	Up-Regulated
FSCN1	1.005	5.74227E-13	7.84292E-12	Up-Regulated
GSDMB	1.077	6.62235E-13	8.90803E-12	Up-Regulated
RNF183	1.186	6.81454E-13	9.10171E-12	Up-Regulated
PTTG1	1.089	7.25558E-13	9.63118E-12	Up-Regulated
SERPINB2	1.831	7.51731E-13	9.93495E-12	Up-Regulated
C1QTNF3	1.038	8.28342E-13	1.08431E-11	Up-Regulated
CDC42EP5	1.369	8.56337E-13	1.1142E-11	Up-Regulated
NNMT	1.499	8.61793E-13	1.11937E-11	Up-Regulated
IL1R2	1.360	9.42321E-13	1.21138E-11	Up-Regulated
AURKA	1.088	9.48464E-13	1.21729E-11	Up-Regulated
FZD7	1.105	9.92186E-13	1.26695E-11	Up-Regulated
IFIT3	1.061	1.21111E-12	1.50452E-11	Up-Regulated
RAB25	1.549	1.22381E-12	1.51905E-11	Up-Regulated
CMPK2	1.011	1.22575E-12	1.52021E-11	Up-Regulated
ROBO1	1.270	1.30972E-12	1.60982E-11	Up-Regulated
FGF14	1.546	1.33946E-12	1.64236E-11	Up-Regulated
PELI1	1.022	1.39069E-12	1.69417E-11	Up-Regulated
CLEC5A	1.196	1.4365E-12	1.7392E-11	Up-Regulated
ABRACL	1.193	1.45235E-12	1.75514E-11	Up-Regulated
TNFRSF11B	1.205	1.73798E-12	2.06561E-11	Up-Regulated
PLAC8	2.011	1.85054E-12	2.19077E-11	Up-Regulated
TMEM154	1.049	1.88921E-12	2.22783E-11	Up-Regulated
CSTA	1.561	1.89723E-12	2.23554E-11	Up-Regulated
ACTA2	1.317	1.90984E-12	2.24864E-11	Up-Regulated
LIPG	1.119	2.08247E-12	2.43105E-11	Up-Regulated
CP	1.717	2.14414E-12	2.49032E-11	Up-Regulated
KIF11	1.211	2.16689E-12	2.51021E-11	Up-Regulated
TPX2	1.051	2.8211E-12	3.19452E-11	Up-Regulated
HILPDA	1.031	3.23456E-12	3.60068E-11	Up-Regulated
COTL1	1.188	3.28959E-12	3.65656E-11	Up-Regulated
PSCA	1.891	3.70686E-12	4.06061E-11	Up-Regulated
ZNF521	1.330	3.84208E-12	4.1905E-11	Up-Regulated
LXN	1.223	3.91008E-12	4.25852E-11	Up-Regulated
ADAM12	1.365	4.24067E-12	4.57242E-11	Up-Regulated
C5orf46	1.143	4.46416E-12	4.77928E-11	Up-Regulated
DHRS9	1.606	4.50451E-12	4.81906E-11	Up-Regulated
GBP1	1.458	4.50928E-12	4.82075E-11	Up-Regulated
IFIT1	1.170	4.55423E-12	4.86192E-11	Up-Regulated

DUSP6	1.077	4.93668E-12	5.22956E-11	Up-Regulated
ID1	1.385	5.06156E-12	5.34311E-11	Up-Regulated
SLC16A1	1.041	5.52143E-12	5.76014E-11	Up-Regulated
RAD51AP1	1.068	5.81156E-12	6.02122E-11	Up-Regulated
CCDC109B	1.240	6.18085E-12	6.33433E-11	Up-Regulated
EPS8L3	1.592	6.29704E-12	6.44465E-11	Up-Regulated
CEMIP	1.332	6.33802E-12	6.47782E-11	Up-Regulated
MUC20	1.023	6.76482E-12	6.86757E-11	Up-Regulated
RRM2	1.293	6.98133E-12	7.06836E-11	Up-Regulated
GBP3	1.490	7.04097E-12	7.10875E-11	Up-Regulated
NUF2	1.256	7.78147E-12	7.77426E-11	Up-Regulated
SLC16A3	1.163	9.07766E-12	8.93919E-11	Up-Regulated
ADAM28	1.061	9.40456E-12	9.21305E-11	Up-Regulated
PLS1	1.662	1.00685E-11	9.7622E-11	Up-Regulated
EZR	1.032	1.01101E-11	9.78996E-11	Up-Regulated
LGALS4	2.041	1.05443E-11	1.01196E-10	Up-Regulated
TTK	1.126	1.22197E-11	1.15514E-10	Up-Regulated
IFITM1	1.031	1.29914E-11	1.21893E-10	Up-Regulated
PDZK1IP1	1.466	1.42537E-11	1.31934E-10	Up-Regulated
MSN	1.045	1.44262E-11	1.33123E-10	Up-Regulated
CLIC3	1.289	1.48269E-11	1.35955E-10	Up-Regulated
HMCN1	1.263	1.62071E-11	1.46777E-10	Up-Regulated
AREG	1.699	1.63141E-11	1.47658E-10	Up-Regulated
IRF7	1.004	1.70301E-11	1.53038E-10	Up-Regulated
IFI16	1.176	1.74167E-11	1.5614E-10	Up-Regulated
GLT8D2	1.108	1.80352E-11	1.61111E-10	Up-Regulated
PPARG	1.023	1.81673E-11	1.62196E-10	Up-Regulated
NREP	1.109	1.99065E-11	1.7575E-10	Up-Regulated
VSIG1	1.814	2.01317E-11	1.77634E-10	Up-Regulated
COL8A1	1.204	2.05518E-11	1.80497E-10	Up-Regulated
KRT6B	1.279	2.38659E-11	2.06601E-10	Up-Regulated
SCEL	1.232	2.44237E-11	2.10946E-10	Up-Regulated
DSG2	1.184	2.7692E-11	2.36387E-10	Up-Regulated
PSMB8	1.040	3.25116E-11	2.7239E-10	Up-Regulated
FLRT3	1.135	3.29429E-11	2.75851E-10	Up-Regulated
SFRP2	1.815	3.43849E-11	2.86338E-10	Up-Regulated
PHACTR3	1.220	3.58874E-11	2.97539E-10	Up-Regulated
MMP12	2.147	4.71547E-11	3.81327E-10	Up-Regulated
GPR87	1.353	5.02891E-11	4.04078E-10	Up-Regulated
TOX3	1.534	5.56197E-11	4.43139E-10	Up-Regulated
LSR	1.191	6.25717E-11	4.89745E-10	Up-Regulated
SFRP4	1.914	6.42451E-11	5.02322E-10	Up-Regulated
RNF145	1.042	6.76187E-11	5.25436E-10	Up-Regulated
CDKN2B	1.077	6.79886E-11	5.27791E-10	Up-Regulated
PRRX1	1.203	6.87445E-11	5.32608E-10	Up-Regulated
ALOX5AP	1.383	7.00964E-11	5.41902E-10	Up-Regulated
ACSL5	1.352	7.19655E-11	5.55095E-10	Up-Regulated
KLK7	1.313	7.95897E-11	6.06907E-10	Up-Regulated
SYT13	1.205	8.08083E-11	6.15267E-10	Up-Regulated
MOXD1	1.287	8.32639E-11	6.3237E-10	Up-Regulated
C9orf152	1.432	9.24155E-11	6.92474E-10	Up-Regulated
TMEM45B	1.321	9.30149E-11	6.9643E-10	Up-Regulated

C1R	1.098	9.92438E-11	7.3489E-10	Up-Regulated
PCSK5	1.049	1.07449E-10	7.87543E-10	Up-Regulated
IRF6	1.078	1.2177E-10	8.81809E-10	Up-Regulated
SPP1	1.573	1.29766E-10	9.35231E-10	Up-Regulated
NCF2	1.244	1.44798E-10	1.0337E-09	Up-Regulated
GPNMB	1.310	1.45843E-10	1.03969E-09	Up-Regulated
MYL9	1.245	1.59559E-10	1.12525E-09	Up-Regulated
KRT18	1.122	1.69825E-10	1.19098E-09	Up-Regulated
TSPAN8	1.851	1.70566E-10	1.19563E-09	Up-Regulated
PLCB4	1.121	1.87122E-10	1.30081E-09	Up-Regulated
CLDN2	1.346	1.93792E-10	1.34409E-09	Up-Regulated
LOX	1.406	1.94504E-10	1.34655E-09	Up-Regulated
CTSC	1.133	2.22578E-10	1.53039E-09	Up-Regulated
TREM1	1.070	2.44615E-10	1.66449E-09	Up-Regulated
ARL14	1.887	2.46616E-10	1.67358E-09	Up-Regulated
COL6A2	1.104	2.69673E-10	1.81133E-09	Up-Regulated
TAGLN	1.202	2.75256E-10	1.84473E-09	Up-Regulated
COL17A1	1.003	3.72999E-10	2.42554E-09	Up-Regulated
PSMB9	1.156	4.07741E-10	2.62211E-09	Up-Regulated
PCOLCE	1.006	4.18709E-10	2.68123E-09	Up-Regulated
CRISPLD1	1.301	4.35228E-10	2.77313E-09	Up-Regulated
MUC16	1.409	4.47633E-10	2.84474E-09	Up-Regulated
GJA1	1.110	4.49878E-10	2.8566E-09	Up-Regulated
MUC5B	1.402	5.43885E-10	3.38104E-09	Up-Regulated
SERPINB3	1.566	6.17616E-10	3.7941E-09	Up-Regulated
CFH	1.021	7.14439E-10	4.33949E-09	Up-Regulated
PLEKHG1	1.031	7.15791E-10	4.34596E-09	Up-Regulated
THBS1	1.270	7.48846E-10	4.53026E-09	Up-Regulated
BIRC3	1.037	7.49465E-10	4.53219E-09	Up-Regulated
GEM	1.159	1.02838E-09	6.05405E-09	Up-Regulated
CHSY1	1.010	1.0327E-09	6.07713E-09	Up-Regulated
PLA2G10	1.041	1.05918E-09	6.22326E-09	Up-Regulated
GALNT3	1.049	1.07759E-09	6.32161E-09	Up-Regulated
HIST1H2BC	1.024	1.16447E-09	6.77337E-09	Up-Regulated
AGR3	1.959	1.16719E-09	6.78397E-09	Up-Regulated
PROM1	1.636	1.22819E-09	7.10029E-09	Up-Regulated
SOX9	1.133	1.36763E-09	7.82556E-09	Up-Regulated
SPON1	1.158	1.53745E-09	8.70166E-09	Up-Regulated
CYP3A5	1.519	1.69432E-09	9.51112E-09	Up-Regulated
CXCL14	1.494	1.73761E-09	9.72161E-09	Up-Regulated
SNAI2	1.096	1.88E-09	1.04179E-08	Up-Regulated
ELF3	1.138	2.12018E-09	1.16084E-08	Up-Regulated
RASEF	1.015	2.31281E-09	1.25764E-08	Up-Regulated
PBK	1.143	2.62141E-09	1.40821E-08	Up-Regulated
CGN	1.055	3.05972E-09	1.62062E-08	Up-Regulated
TRIM31	1.006	3.3439E-09	1.75331E-08	Up-Regulated
HLA-B	1.026	3.66143E-09	1.90591E-08	Up-Regulated
KLK6	1.078	4.11824E-09	2.11887E-08	Up-Regulated
SULT1C2	1.442	4.46631E-09	2.27548E-08	Up-Regulated
BASP1	1.004	4.70389E-09	2.38366E-08	Up-Regulated
HSD17B6	1.103	4.75381E-09	2.40654E-08	Up-Regulated
MUC1	1.410	5.01873E-09	2.53385E-08	Up-Regulated

PMP22	1.033	5.20828E-09	2.62429E-08	Up-Regulated
ODAM	1.256	5.26415E-09	2.65042E-08	Up-Regulated
MFAP5	1.455	6.06717E-09	3.01482E-08	Up-Regulated
APOC1	1.076	7.67199E-09	3.72048E-08	Up-Regulated
XDH	1.088	8.06859E-09	3.89406E-08	Up-Regulated
SLC26A9	1.361	8.7117E-09	4.1698E-08	Up-Regulated
EGR2	1.063	1.20023E-08	5.61368E-08	Up-Regulated
CYP4F3	1.191	1.26475E-08	5.89124E-08	Up-Regulated
AKR1C3	1.209	1.44312E-08	6.65218E-08	Up-Regulated
PLEKHA2	1.043	1.5081E-08	6.92441E-08	Up-Regulated
BCL2A1	1.427	1.69932E-08	7.73191E-08	Up-Regulated
PTPN12	1.076	1.85753E-08	8.36856E-08	Up-Regulated
CST6	1.077	2.12425E-08	9.43236E-08	Up-Regulated
OLFM4	2.251	2.14327E-08	9.51124E-08	Up-Regulated
IL7	1.036	2.25141E-08	9.93857E-08	Up-Regulated
DUSP5	1.005	2.32028E-08	1.02038E-07	Up-Regulated
CYP1B1	1.448	2.32558E-08	1.02212E-07	Up-Regulated
MUC13	1.198	3.38845E-08	1.44024E-07	Up-Regulated
HAS3	1.032	3.49404E-08	1.4797E-07	Up-Regulated
SPRR3	1.380	4.57081E-08	1.89117E-07	Up-Regulated
TFF2	1.942	4.61486E-08	1.90835E-07	Up-Regulated
PLA2G7	1.084	4.95178E-08	2.03045E-07	Up-Regulated
GIMAP2	1.173	5.75441E-08	2.33296E-07	Up-Regulated
EPYC	1.617	6.60456E-08	2.64849E-07	Up-Regulated
SLCO1B3	1.216	8.08402E-08	3.1926E-07	Up-Regulated
LY96	1.261	9.14146E-08	3.56548E-07	Up-Regulated
CCL18	1.263	9.68537E-08	3.75435E-07	Up-Regulated
RGS1	1.106	9.72708E-08	3.76859E-07	Up-Regulated
F2RL2	1.129	1.19647E-07	4.55033E-07	Up-Regulated
C3	1.154	1.62999E-07	6.08116E-07	Up-Regulated
CLEC2B	1.012	2.06775E-07	7.56854E-07	Up-Regulated
GDA	1.098	2.25459E-07	8.18752E-07	Up-Regulated
TYROBP	1.075	2.85602E-07	1.01462E-06	Up-Regulated
PTGS2	1.334	2.87499E-07	1.02043E-06	Up-Regulated
STMN2	1.103	3.30991E-07	1.16089E-06	Up-Regulated
LYZ	1.804	4.12868E-07	1.4236E-06	Up-Regulated
C5AR1	1.046	4.53852E-07	1.55357E-06	Up-Regulated
SUCNR1	1.012	4.61086E-07	1.57654E-06	Up-Regulated
REG4	2.122	5.30651E-07	1.79691E-06	Up-Regulated
CXCR4	1.169	5.3842E-07	1.8224E-06	Up-Regulated
MMP10	1.113	5.96684E-07	2.00078E-06	Up-Regulated
TFF3	1.248	7.11225E-07	2.3549E-06	Up-Regulated
LY6D	1.252	7.91683E-07	2.59812E-06	Up-Regulated
CHI3L1	1.135	8.01269E-07	2.62616E-06	Up-Regulated
AKR1B10	1.549	1.40075E-06	4.40461E-06	Up-Regulated
VNN1	1.201	1.41073E-06	4.43045E-06	Up-Regulated
CLRN3	1.370	1.63449E-06	5.07097E-06	Up-Regulated
RNF128	1.217	1.73336E-06	5.35135E-06	Up-Regulated
IGFBP1	1.032	2.41993E-06	7.2936E-06	Up-Regulated
FCER1G	1.014	3.6384E-06	1.06453E-05	Up-Regulated
DMBT1	1.282	5.95487E-06	1.68233E-05	Up-Regulated
HSD17B2	1.153	7.64269E-06	2.11254E-05	Up-Regulated

HPGD	1.069	9.79595E-06	2.64956E-05	Up-Regulated
EPCAM	1.121	1.09428E-05	2.93302E-05	Up-Regulated
CYP2C18	1.003	2.34638E-05	5.91529E-05	Up-Regulated
ACTG2	1.070	0.000247756	0.000515652	Up-Regulated
CDH17	1.044	0.0002552	0.000529546	Up-Regulated
FCGR3A	1.110	0.000476806	0.00094212	Up-Regulated
CRISP3	1.191	0.002721655	0.004742021	Up-Regulated
MYRIP	-1.482	8.40241E-22	1.17525E-19	Down-Regulated
CBFA2T3	-1.169	8.62691E-22	1.19558E-19	Down-Regulated
PDCD4	-1.060	2.46429E-19	1.84759E-17	Down-Regulated
BBIP1	-1.116	1.17597E-18	7.69011E-17	Down-Regulated
BTG2	-1.234	1.53958E-18	9.65015E-17	Down-Regulated
FAM46C	-1.363	2.14506E-18	1.28076E-16	Down-Regulated
ACAT1	-1.063	8.59424E-18	4.27055E-16	Down-Regulated
P2RX1	-1.575	2.33424E-17	1.02802E-15	Down-Regulated
PAIP2B	-1.700	3.15264E-17	1.34152E-15	Down-Regulated
FKBP11	-1.162	5.88817E-17	2.37825E-15	Down-Regulated
ECHDC3	-1.105	6.35463E-17	2.5395E-15	Down-Regulated
PTGER4	-1.088	2.19625E-16	7.50602E-15	Down-Regulated
KLB	-1.293	3.43929E-16	1.09377E-14	Down-Regulated
CA4	-1.128	3.88208E-16	1.21414E-14	Down-Regulated
BRSK2	-1.016	4.96551E-16	1.52148E-14	Down-Regulated
TPST2	-1.987	5.39493E-16	1.63646E-14	Down-Regulated
CCDC110	-1.689	9.47242E-16	2.67459E-14	Down-Regulated
GPHA2	-1.654	1.96692E-15	5.08771E-14	Down-Regulated
FAM129A	-1.181	2.00717E-15	5.1653E-14	Down-Regulated
MT1G	-1.387	2.55929E-15	6.43272E-14	Down-Regulated
SLC39A5	-1.462	3.3546E-15	8.08206E-14	Down-Regulated
GUCA1C	-1.778	3.71712E-15	8.89871E-14	Down-Regulated
AKAP7	-1.249	5.4876E-15	1.25981E-13	Down-Regulated
CTNND2	-1.372	7.14833E-15	1.6045E-13	Down-Regulated
MAT1A	-1.205	7.29331E-15	1.62977E-13	Down-Regulated
TRHDE	-1.871	7.37905E-15	1.64407E-13	Down-Regulated
SPX	-1.919	9.0344E-15	1.97788E-13	Down-Regulated
PSAT1	-1.776	9.54439E-15	2.0745E-13	Down-Regulated
LIFR	-1.129	1.14751E-14	2.44146E-13	Down-Regulated
LGALS2	-1.847	1.55359E-14	3.18866E-13	Down-Regulated
PM20D1	-1.984	1.58022E-14	3.23892E-13	Down-Regulated
CCKBR	-1.211	2.0991E-14	4.20545E-13	Down-Regulated
MT1F	-1.171	2.29597E-14	4.5456E-13	Down-Regulated
ECI2	-1.042	2.94533E-14	5.74093E-13	Down-Regulated
SLC16A10	-1.698	3.23866E-14	6.20066E-13	Down-Regulated
SEL1L	-1.494	3.59901E-14	6.81287E-13	Down-Regulated
MT1H	-1.128	3.65473E-14	6.90967E-13	Down-Regulated
C5	-1.451	4.49308E-14	8.3279E-13	Down-Regulated
SLC1A2	-1.176	5.12602E-14	9.30693E-13	Down-Regulated
ALB	-2.309	5.18738E-14	9.37512E-13	Down-Regulated
ERO1LB	-1.780	5.50567E-14	9.8775E-13	Down-Regulated
GNMT	-2.376	7.85779E-14	1.34886E-12	Down-Regulated
EPHX2	-1.174	1.06802E-13	1.76322E-12	Down-Regulated
SPACA3	-1.199	1.17106E-13	1.91243E-12	Down-Regulated
AQP12B	-1.489	1.71105E-13	2.67846E-12	Down-Regulated

SERPINI1	-1.302	3.62392E-13	5.19876E-12	Down-Regulated
PDK4	-1.768	5.31427E-13	7.30626E-12	Down-Regulated
NUCB2	-1.276	5.52708E-13	7.57642E-12	Down-Regulated
GLS2	-1.115	5.6076E-13	7.67981E-12	Down-Regulated
BACE1	-1.465	5.91272E-13	8.03939E-12	Down-Regulated
AOX1	-1.697	9.37851E-13	1.20778E-11	Down-Regulated
NRG4	-1.215	9.97368E-13	1.27249E-11	Down-Regulated
KIAA1324	-1.452	1.00066E-12	1.2755E-11	Down-Regulated
F8	-1.178	1.00765E-12	1.28127E-11	Down-Regulated
FAM150B	-1.863	1.0731E-12	1.35199E-11	Down-Regulated
TMEM52	-1.770	1.40472E-12	1.70989E-11	Down-Regulated
SLC30A2	-1.110	1.99525E-12	2.34372E-11	Down-Regulated
CTH	-1.137	2.07194E-12	2.4225E-11	Down-Regulated
BNIP3	-1.512	2.18662E-12	2.52918E-11	Down-Regulated
KCNK3	-1.176	4.35528E-12	4.68262E-11	Down-Regulated
TEX11	-2.158	6.38059E-12	6.5133E-11	Down-Regulated
RBPJL	-1.938	6.99278E-12	7.07522E-11	Down-Regulated
RGN	-1.284	7.45329E-12	7.47606E-11	Down-Regulated
SLC43A1	-1.398	9.76318E-12	9.52115E-11	Down-Regulated
MT1M	-1.488	9.81885E-12	9.56309E-11	Down-Regulated
EPHX1	-1.428	1.03011E-11	9.94046E-11	Down-Regulated
PRDX4	-1.117	1.13658E-11	1.07847E-10	Down-Regulated
AQP8	-2.859	1.3214E-11	1.2363E-10	Down-Regulated
EGF	-2.062	1.46148E-11	1.34371E-10	Down-Regulated
ZG16	-1.340	1.47795E-11	1.35637E-10	Down-Regulated
KIRREL2	-1.070	1.59822E-11	1.45002E-10	Down-Regulated
TMED6	-2.716	1.67971E-11	1.51214E-10	Down-Regulated
ACADL	-1.485	2.39616E-11	2.0731E-10	Down-Regulated
PLCE1	-1.272	2.8532E-11	2.42819E-10	Down-Regulated
GCAT	-1.021	2.99221E-11	2.52939E-10	Down-Regulated
RNF186	-1.488	3.21021E-11	2.69109E-10	Down-Regulated
PDIA2	-2.318	3.58459E-11	2.97358E-10	Down-Regulated
LMO3	-1.314	4.8459E-11	3.91037E-10	Down-Regulated
GAS2	-1.259	4.89485E-11	3.93956E-10	Down-Regulated
DPEP1	-1.261	9.81142E-11	7.28311E-10	Down-Regulated
ANPEP	-2.043	1.60954E-10	1.13351E-09	Down-Regulated
CXCL12	-1.111	2.186E-10	1.50509E-09	Down-Regulated
NR5A2	-1.683	3.26824E-10	2.15307E-09	Down-Regulated
SLC39A14	-1.132	3.79902E-10	2.46406E-09	Down-Regulated
TSPAN7	-1.059	5.02373E-10	3.15477E-09	Down-Regulated
ABAT	-1.126	7.637E-10	4.60537E-09	Down-Regulated
ERP27	-2.723	1.21515E-09	7.04108E-09	Down-Regulated
REEP1	-1.065	1.42719E-09	8.13549E-09	Down-Regulated
KLK1	-2.217	1.43191E-09	8.15322E-09	Down-Regulated
RNASE1	-1.188	1.47475E-09	8.38136E-09	Down-Regulated
HOMER2	-1.319	2.964E-09	1.57379E-08	Down-Regulated
SERPINI2	-3.170	3.17071E-09	1.67471E-08	Down-Regulated
SPAG4	-1.014	3.92013E-09	2.02522E-08	Down-Regulated
PAK3	-1.011	3.97762E-09	2.05211E-08	Down-Regulated
CTRL	-3.077	5.40512E-09	2.71488E-08	Down-Regulated
FGL1	-1.844	6.59196E-09	3.24465E-08	Down-Regulated
NRCAM	-1.102	6.90993E-09	3.38571E-08	Down-Regulated

BEX5	-1.033	7.49611E-09	3.64455E-08	Down-Regulated
PDZK1	-1.118	1.59658E-08	7.28861E-08	Down-Regulated
GSTA1	-1.704	3.50039E-08	1.48198E-07	Down-Regulated
NPY1R	-1.280	3.85082E-08	1.61316E-07	Down-Regulated
EPB41L4B	-1.091	3.95106E-08	1.65149E-07	Down-Regulated
DPT	-1.140	4.88095E-08	2.0063E-07	Down-Regulated
CELA2B	-2.910	7.96277E-08	3.15049E-07	Down-Regulated
IMPA2	-1.271	2.57283E-07	9.23381E-07	Down-Regulated
SYCN	-2.962	3.50146E-07	1.22324E-06	Down-Regulated
TMEM97	-1.307	3.63873E-07	1.26689E-06	Down-Regulated
PNLIPRP1	-2.740	4.21575E-07	1.45097E-06	Down-Regulated
SLC38A5	-1.031	4.52858E-07	1.55051E-06	Down-Regulated
SYBU	-1.169	4.89612E-07	1.66691E-06	Down-Regulated
KCNJ16	-1.465	6.22018E-07	2.07973E-06	Down-Regulated
IGFBP2	-1.565	1.31278E-06	4.14785E-06	Down-Regulated
SLC4A4	-1.317	1.59884E-06	4.96649E-06	Down-Regulated
CUZD1	-2.539	1.83578E-06	5.64345E-06	Down-Regulated
CPA2	-2.602	2.01216E-06	6.14675E-06	Down-Regulated
IL22RA1	-1.023	2.49217E-06	7.49188E-06	Down-Regulated
GP2	-2.474	2.93914E-06	8.72788E-06	Down-Regulated
ADH1B	-1.178	3.72565E-06	1.089E-05	Down-Regulated
HBB	-1.189	4.08678E-06	1.18607E-05	Down-Regulated
BEX1	-1.253	4.10013E-06	1.18949E-05	Down-Regulated
IAPP	-1.846	4.65086E-06	1.33516E-05	Down-Regulated
HBA2	-1.142	7.00797E-06	1.95246E-05	Down-Regulated
HBA1	-1.130	7.81029E-06	2.1555E-05	Down-Regulated
CLPS	-2.545	1.06328E-05	2.85799E-05	Down-Regulated
CTRC	-2.378	1.46022E-05	3.81496E-05	Down-Regulated
LYVE1	-1.014	1.83383E-05	4.70719E-05	Down-Regulated
PRSS3	-1.765	1.86821E-05	4.78974E-05	Down-Regulated
CELA2A	-2.554	3.81136E-05	9.19572E-05	Down-Regulated
AMY1B	-1.891	3.8715E-05	9.32592E-05	Down-Regulated
PLA2G1B	-2.451	7.56929E-05	0.000173219	Down-Regulated
CELA3B	-2.147	0.000105385	0.000234975	Down-Regulated
AZGP1	-1.267	0.000116793	0.00025854	Down-Regulated
CEL	-2.232	0.000126807	0.000278625	Down-Regulated
FAM3B	-1.154	0.00016175	0.000348669	Down-Regulated
CPA1	-2.353	0.00017442	0.000373955	Down-Regulated
CELA3A	-2.034	0.000322585	0.000657621	Down-Regulated
C6	-1.022	0.000326766	0.000665335	Down-Regulated
CTRB2	-1.893	0.000660523	0.001273336	Down-Regulated
PRSS1	-1.802	0.000969536	0.001814172	Down-Regulated
PNLIP	-1.942	0.001089736	0.002023298	Down-Regulated
REG1B	-1.863	0.00111134	0.002060371	Down-Regulated
REG1A	-1.512	0.001363792	0.002495027	Down-Regulated
CPB1	-1.805	0.001696906	0.003048698	Down-Regulated
REG3G	-1.111	0.007999744	0.012858495	Down-Regulated
REG3A	-1.015	0.031836087	0.045884547	Down-Regulated

Table S2. A list of 1791 MTGs with expression level >=0.5 TPM in PDAC derived from HCMDB

**Genes**
AAMP, ABCA2, ABCB1, ABCC5, ABCG2, ABI1, ABI2, ABL1, ABL2, ACE, ACE2, ACKR1, ACKR2, ACKR3, ACOT8, ACP5, ACTA2, ACTB, ACTL6A, ACTN4, ACTR2, ACTR3, ACVR1C, ADAM10, ADAM12, ADAM15, ADAM17, ADAM23, ADAM28, ADAM8, ADAM9, ADAMTS1, ADAMTS13, ADAMTS18, ADIPOQ, ADM, ADORA2B, ADRB2, AFAP1L1, AFAP1L2, AGER, AGO1, AGO2, AGO4, AGR2, AGR3, AGT, AGTR1, AHR, AIFM1, AJUBA, AKAP12, AKR1C2, AKR1C3, AKT1, AKT2, AKT3, ALCAM, ALDH1A1, ALDH1A3, ALDH3A1, ALDH7A1, ALDOA, ALK, ALOX5, ALPL, ALPP, ALYREF, AMACR, AMBP, AMFR, AMOT, ANGPT1, ANGPT2, ANGPTL2, ANGPTL4, ANGPTL6, ANKRD12, ANO1, ANO9, ANTXR1, ANXA1, ANXA2, ANXA2R, ANXA3, ANXA4, ANXA7, APAF1, APBA2, APC, APEX1, API5, APMAP, APOBEC3G, APOM, AQP1, AQP3, AQP5, AQP8, AR, ARF6, ARG1, ARHGAP35, ARHGAP5, ARHGDIA, ARHGDIB, ARHGEF1, ARHGEF2, ARHGEF39, ARID1A, ARID4A, ARID4B, ARL2BP, ARL6IP5, ARMC8, ARNT, ARNTL, ARRB1, ARTN, ASAP1, ASCL2, ASPN, ASS1, ATAD2, ATF3, ATF4, ATG10, ATM, ATP11A, ATP2A3, ATP5B, ATP6V1C1, ATPIF1, AURKA, AURKB, AUTS2, AXIN2, AXL, B2M, B3GAT1, B3GNT3, B3GNT7, B3GNT8, B4GALT1, B4GALT3, BAD, BAG1, BAG2, BAG3, BAIAP2, BAK1, BAP1, BAX, BAZ2A, BBC3, BCAM, BCAR1, BCAT1, BCL2, BCL2L1, BCL2L11, BCL2L12, BCL2L2, BCL9, BDKRB2, BDNF, BECN1, BGN, BIN1, BIRC2, BIRC3, BIRC5, BIRC7, BMI1, BMP1, BMP2, BMP4, BMP5, BMP6, BMP7, BNIP3, BRAF, BRCA1, BRCA2, BRD7, BRMS1, BRS3, BSG, BST2, BTG1, BVES, C14orf166, C1QA, C1QBP, C6orf106, CA2, CA9, CACUL1, CACYBP, CADM1, CALCA, CALR, CALU, CAMP, CANX, CAP1, CAPG, CAPNS1, CARD10, CARD9, CASP10, CASP3, CASP8, CASP9, CASS4, CAT, CAV1, CBL, CBLB, CBR1, CBR3, CBX4, CBX5, CBX7, CBX8, CCAR2, CCDC88A, CCDC88C, CCL14, CCL18, CCL19, CCL2, CCL21, CCL22, CCL3, CCL5, CCL7, CCL8, CCNA2, CCNB1, CCNB2, CCND1, CCND2, CCNG1, CCNG2, CCR1, CCR4, CCR5, CCR7, CCR9, CD109, CD14, CD151, CD164, CD200, CD22, CD24, CD274, CD276, CD34, CD4, CD40, CD44, CD47, CD55, CD63, CD74, CD80, CD81, CD82, CD9, CDC42, CDC73, CDCA8, CDCP1, CDH1, CDH11, CDH12, CDH13, CDH17, CDH2, CDH3, CDK1, CDK4, CDK5RAP3, CDK8, CDKN1A, CDKN1B, CDKN2A, CDX2, CEACAM1, CEACAM3, CEACAM5, CEACAM6, CEBPA, CEBPB, CEMIP, CENPW, CERS2, CERS6, CFL1, CFTR, CHAF1B, CHD1L, CHEK1, CHEK2, CHFR, CHGA, CHI3L1, CHRM3, CHRNA7, CHUK, CITED1, CITED2, CKS1B, CLCA1, CLCA2, CLCN3, CLDN1, CLDN11, CLDN16, CLDN18, CLDN3, CLDN4, CLDN5, CLDN7, CLEC4G, CLIC1, CLIC3, CLIC4, CLIP1, CLOCK, CLU, CMTM3, CNBP, CNR1, CNTN1, COL18A1, COL1A1, COL3A1, COL4A6, COPS3, CORO1C, CPE, CPEB2, CPEB4, CPNE3, CR1, CRABP2, CREB1, CREB3, CREB5, CREM, CRIM1, CRK, CRKL, CRMP1, CRP, CRTC2, CRYAB, CSE1L, CSF1, CSNK1A1, CSNK2A1, CSNK2B, CSPG4, CST3, CST6, CSTA, CTGF, CTHRC1, CTLA4, CTNNA1, CTNNAL1, CTNNB1, CTNND1, CTNND2, CTSB, CTSD, CTSH, CTSK, CTSL, CTSS, CTSZ, CTTN, CUL1, CUX1, CX3CR1, CXADR, CXCL1, CXCL10, CXCL12, CXCL14, CXCL2, CXCL3, CXCL6, CXCL8, CXCR1, CXCR2, CXCR3, CXCR4, CXCR6, CXXC4, CYB5R3, CYCS, CYLD, CYP1B1, CYP2J2, CYP3A4, CYP3A5, CYR61, DAB2, DAB2IP, DACH1, DACT2, DAG1, DAPK1, DDA1, DDB2, DDIT3, DDR1, DDR2, DEK, DEPDC1B, DEPTOR, DHFR, DIABLO, DIAPH1, DICER1, DIRAS1, DIRAS3, DIXDC1, DKK1, DKK3, DKK4, DLC1, DLEC1, DLL1, DLL4, DNAJA3, DNAJB4, DNAJB6, DNM2, DNMT3A, DNMT3B, DOCK1, DOCK4, DPEP1, DPP4, DPYD, DPYSL3, DRG1, DROSHA, DSC3, DSG3, DUSP1, DUSP6, DVL2, E2F1, E2F3, EBAG9, ECE1, ECE2, ECM1, ECT2, EDN1, EDNRA, EDNRB, EFEMP1, EFNB1, EFNB2, EGF, EGFL7, EGFR, EGLN3, EGR1, EGR3, EHD2, EHMT2, EIF1AX, EIF3I, EIF4B, EIF4E, EIF4EBP1, EIF5A2, EIF6, ELAVL1, ELAVL4, ELF1, ELF5, ELK3, ELMO1, EMP1, EMP3, ENAH, ENG, ENO1, ENO2, ENPP1, ENPP2, ENTPD1, EP300, EPAS1, EPB41L3, EPC1, EPCAM,

EPHA1, EPHA10, EPHA2, EPHA4, EPHA7, EPHB1, EPHB2, EPHB3, EPHB4, EPOR, EPS8, EPSTI1, ERAP1, ERBB2, ERBB3, ERCC1, EREG, ERG, ERP29, ESAM, ESM1, ESR1, ESRP1, ETS1, ETS2, ETV1, ETV4, ETV7, EVI5, EVL, EYA2, EZH2, EZR, F11R, F2R, F2RL1, F3, F7, FABP1, FABP3, FABP5, FADD, FAIM2, FAM120A, FAM134A, FAM3B, FANCD2, FAP, FAS, FASLG, FASN, FBLN5, FBN1, FBN2, FBXO11, FBXW7, FCGRT, FDFT1, FER, FERMT1, FERMT2, FERMT3, FGF1, FGF19, FGF2, FGFBP1, FGFR1, FGFR2, FGFR3, FGFR4, FGL2, FHIT, FHL2, FILIP1, FKBP5, FLI1, FLNA, FLOT1, FLOT2, FLT1, FLT4, FMNL2, FMNL3, FN1, FNDC3B, FOLH1, FOS, FOSB, FOSL1, FOXA2, FOXC1, FOXC2, FOXD3, FOXF2, FOXJ1, FOXJ2, FOXK1, FOXL1, FOXM1, FOXN3, FOXO1, FOXO3, FOXO4, FOXP3, FOXQ1, FPR2, FRAT1, FSCN1, FSCN2, FST, FSTL1, FUBP1, FURIN, FUS, FXR1, FXYD3, FXYD5, FYN, FZD2, FZD7, G3BP1, G3BP2, G6PD, GAB1, GAB2, GADD45A, GAL3ST2, GALNT3, GAS1, GAS6, GATA3, GATA4, GATA6, GBP2, GCNT1, GCNT2, GDF15, GDNF, GEMIN2, GFRA1, GGCT, GHITM, GHR, GHRL, GIT1, GJA1, GJB1, GJB2, GKN1, GKN2, GLG1, GLI1, GLI2, GLI3, GLRX3, GMNN, GNA12, GNA13, GNAI1, GNB3, GNPTG, GOLPH3, GPC3, GPC5, GPER1, GPI, GPNMB, GPR4, GPR68, GPR87, GPX1, GPX3, GRB2, GRB7, GRINA, GRM4, GRN, GSC, GSDMB, GSK3B, GSN, GSTM1, GSTM3, GSTP1, GUSB, GZMB, H2AFX, HAMP, HAS1, HAX1, HBEGF, HDAC1, HDAC10, HDAC2, HDAC4, HDGF, HES1, HEY1, HGF, HIC1, HIF1A, HIP1, HK1, HK2, HMGA1, HMGA2, HMGB1, HMMR, HMOX1, HNF1A, HNF4A, HNRNPA1, HNRNPAB, HNRNPD, HNRNPK, HNRNPM, HOMER1, HOPX, HOXA1, HOXA5, HOXA9, HOXB13, HOXB5, HOXB7, HOXB9, HOXC8, HOXD9, HP, HPN, HPSE, HRAS, HRC, HSF1, HSP90AA1, HSP90AB1, HSP90B1, HSPA4, HSPA5, HSPA9, HSPB1, HSPB2, HSPD1, HTATIP2, HTRA1, HTRA2, HTRA3, HTT, HVCN1, HYAL1, HYAL3, IBSP, ICAM1, ID1, ID2, ID3, IDH1, IDO1, IFIT2, IFITM1, IFNAR2, IGF1, IGF1R, IGF2BP1, IGF2BP3, IGFBP1, IGFBP2, IGFBP3, IGFBP4, IGFBP5, IGFBP7, IGSF8, IHH, IKBKB, IKBKE, IL10, IL11, IL12A, IL13RA1, IL13RA2, IL17RA, IL17RB, IL18, IL1A, IL1B, IL1RL1, IL23A, IL24, IL2RG, IL32, IL34, IL4R, IL6, IL7, IL7R, ILK, IMP3, ING1, ING4, ING5, INHBA, INPP4B, INPPL1, INS, IQGAP1, IQSEC1, IRAK1, IRF7, IRS1, IRS2, IRX1, IRX2, ISG15, ITGA2, ITGA3, ITGA4, ITGA5, ITGA6, ITGAM, ITGAV, ITGB1, ITGB2, ITGB3, ITGB3BP, ITGB4, ITGB5, ITGB6, ITGB8, ITIH1, ITIH3, IVNS1ABP, JAG1, JAG2, JAK1, JAK2, JAM3, JDP2, JUN, JUP, KARS, KAT5, KATNA1, KCNMA1, KCNN4, KDM1A, KDM3A, KDM4B, KDM4C, KDM5A, KDM6B, KDR, KEAP1, KHDRBS1, KIAA0100, KIAA1524, KIF14, KIF2C, KIF3C, KISS1, KISS1R, KIT, KITLG, KL, KLF2, KLF4, KLF5, KLF6, KLF8, KLK13, KLK3, KLK4, KLK6, KLK7, KLK8, KRAS, KRT14, KRT17, KRT18, KRT19, KRT20, KRT5, KRT6A, KRT7, KRT8, L1CAM, LAMA5, LAMB3, LAMC2, LAMP3, LAMTOR1, LAP3, LAPTM4B, LARP7, LASP1, LAT, LATS2, LCN2, LCP1, LDHA, LECT1, LEF1, LEP, LEPR, LGALS1, LGALS2, LGALS3, LGALS3BP, LGALS8, LGALS9, LGI4, LGR4, LGR5, LIF, LIFR, LIMA1, LIMK1, LIMK2, LIMS1, LIPH, LLGL1, LLGL2, LMCD1, LMNA, LOX, LOXL2, LOXL4, LPAR1, LPAR2, LRG1, LRP5, LRP6, LRRC26, LRRFIP1, LTB4R2, LTBP1, LYN, LYNX1, LYPD3, LYVE1, LZTS1, MACC1, MACROD1, MAEL, MAF, MAGEA3, MAGED1, MAL, MAML1, MAOA, MAP1LC3A, MAP1LC3B, MAP2, MAP2K4, MAP2K7, MAP3K1, MAP3K11, MAP3K7, MAP7D3, MAPK1, MAPK12, MAPK13, MAPK14, MAPK3, MAPK7, MAPK8, MAPK9, MAPKAP1, MARCKS, MARK2, MASP2, MBD1, MBD2, MBNL1, MCAM, MCL1, MCTS1, MDC1, MDK, MDM2, MECP2, MED19, MED23, MET, METAP2, MFAP3L, MGAT3, MGAT5, MGEA5, MGMT, MIA, MIA3, MICA, MIEN1, MIF, MITF, MKI67, MKL1, MLF2, MLH1, MME, MMP1, MMP10, MMP11, MMP12, MMP13, MMP14, MMP15, MMP16, MMP17, MMP2, MMP28, MMP3, MMP7, MMP9, MMS19, MOB1A, MPP3, MPZL1, MRC2, MRPL44, MSH2, MSI1, MSLN, MSN, MSR1, MSRA, MST1, MST1R, MTA1, MTA2, MTA3, MTAP, MTBP, MTDH, MTHFR, MTNR1A, MTOR, MTSS1, MUC1, MUC13, MUC16, MUC20, MUC4, MUC5AC, MUC6, MUCL1, MX1, MXD1, MYB, MYC, MYCN, MYD88, MYH9, MYO10, MYO1B, MYO5A, MYO9B, MZF1, NAA10, NAB2, NAMPT, NAT1, NBN, NCL, NCOA2, NCOA3, NCOA4,

NCOR2, NCR3LG1, NDC80, NDRG1, NDRG2, NDUFA13, NDUFB9, NEDD4, NEDD4L, NEDD9, NES, NET1, NEU1, NF2, NFATC1, NFATC2, NFE2L2, NFIB, NFKB1, NFKB2, NGF, NGFR, NISCH, NLRP1, NLRP3, NME1, NME2, NME3, NME9, NMU, NOB1, NOD2, NOG, NOL3, NOS2, NOS3, NOTCH1, NOTCH3, NOX1, NPEPPS, NPPA, NR1H4, NR2C2, NR2F2, NR4A1, NR4A2, NRAS, NRF1, NRG1, NRP1, NRP2, NT5E, NTN1, NTRK1, NTRK2, NUAK1, NUCB2, NUCKS1, NUDCD1, NUPR1, OCIAD1, OCLN, ODAM, ODC1, OGG1, OGT, OLR1, ONECUT1, OPCML, ORAI1, ORM2, OSBPL5, OSM, OTUB1, P2RY2, P4HA2, PAG1, PAK1, PAK4, PALLD, PAQR3, PARD3, PARD6A, PARK7, PARP1, PARVA, PARVB, PAX5, PAX8, PBX3, PBXIP1, PCBP1, PCDH10, PCDH9, PCMT1, PCNA, PCSK9, PDCD1, PDCD4, PDCD6IP, PDGFB, PDGFRA, PDGFRB, PDIA3, PDLIM1, PDLIM7, PDPN, PEAK1, PEBP1, PEBP4, PECAM1, PELP1, PER1, PFN1, PGF, PGK1, PGR, PHB, PIAS1, PIEZO1, PIGR, PIK3CA, PIK3CG, PIK3R1, PIM1, PIN1, PIP, PIP4K2A, PITPNM1, PITPNM3, PITX1, PIWIL1, PIWIL2, PIWIL4, PKD1, PKM, PKP3, PLA1A, PLA2G10, PLA2G16, PLA2G2A, PLA2G3, PLAGL1, PLAU, PLAUR, PLCE1, PLD1, PLD2, PLEC, PLK1, PLK4, PLOD2, PLS3, PLXDC1, PLXNB1, PLXND1, PMEL, PML, PMP22, PODXL, POMC, POSTN, POU5F1, PPA1, PPARD, PPARG, PPARGC1A, PPIA, PPM1D, PPP1R13L, PPP1R9B, PPP2CA, PPP3CA, PPT1, PRAF2, PRAME, PRDM2, PRDX1, PRDX2, PRDX4, PRDX6, PREP, PREX1, PREX2, PRKAA1, PRKCA, PRKCE, PRKCG, PRKCI, PRKCZ, PRKD1, PRKD2, PRKD3, PRKDC, PRLR, PRMT1, PRMT7, PRNP, PROC, PROM1, PROX1, PRRX1, PRSS3, PRSS8, PSAP, PSCA, PSMA7, PSMD10, PSME2, PSME3, PTAFR, PTCH1, PTEN, PTGER1, PTGER3, PTGES, PTGIS, PTGS1, PTGS2, PTH1R, PTHLH, PTK2, PTK2B, PTK6, PTK7, PTP4A1, PTP4A2, PTP4A3, PTPN1, PTPN11, PTPN13, PTPN2, PTPN3, PTPN6, PTPRD, PTPRR, PTTG1, PTX3, PXN, RAB11FIP4, RAB1A, RAB1B, RAB22A, RAB23, RAB25, RAB27B, RAB37, RAB3D, RAB40B, RAB5A, RABEP1, RABGEF1, RAC1, RAC2, RAC3, RACGAP1, RAD21, RAD51, RADIL, RAF1, RALA, RALB, RALBP1, RALGAPA2, RALGDS, RAN, RAP1A, RAP1B, RAPGEF3, RARB, RARG, RARRES3, RASA1, RASAL2, RASSF1, RASSF10, RASSF2, RASSF5, RB1, RBBP8, RBM5, RCN1, RDX, RECK, RECQL, REG4, RELA, RELB, RERG, RET, RETN, RETNLB, RFWD2, RGS16, RGS2, RGS5, RHOA, RHOB, RHOC, RICTOR, RIOK3, RLN2, RNASE1, RNF111, RNF180, RNF41, RNH1, ROBO1, ROCK1, ROCK2, ROR1, RPL39, RPS27, RPS6KA3, RPS6KB1, RPSA, RRAS2, RRM1, RRP1B, RSF1, RTKN, RUNX2, RUNX3, RXRA, S100A10, S100A11, S100A14, S100A2, S100A4, S100A6, S100A7, S100A8, S100A9, S100B, S100P, S1PR1, SAA1, SAE1, SALL4, SAMD9, SATB1, SATB2, SCARA3, SCARB2, SCGB2A1, SCGB2A2, SCGB3A1, SCGN, SCN1B, SCRN1, SCUBE2, SDC1, SDC2, SDC4, SDCBP, SDHB, SDPR, SELE, SELP, SELPLG, SEMA3E, SEMA3F, SEMA4B, SEMA4C, SEMA4D, SEMA5A, SEMA6B, SENP1, SENP3, SERBP1, SERPINA5, SERPINB3, SERPINB4, SERPINB5, SERPIND1, SERPINE1, SERPINE2, SERPINF1, SESN2, SET, SETD1A, SETD2, SETDB1, SFN, SFRP1, SFRP2, SFTPA1, SGK1, SH2B1, SHC1, SHH, SIAH1, SIK1, SIRT1, SIRT6, SIRT7, SIX1, SKIL, SKP2, SLC16A4, SLC1A5, SLC2A1, SLC2A3, SLC39A4, SLC39A6, SLC3A2, SLC9A1, SLIT2, SLITRK3, SLPI, SMAD1, SMAD2, SMAD3, SMAD4, SMAD6, SMAD7, SMARCA4, SMARCB1, SMARCC1, SMARCE1, SMO, SMPD3, SMURF1, SMURF2, SMYD3, SNAI1, SNAI2, SNCG, SND1, SOCS1, SOCS3, SOD2, SOD3, SOX12, SOX17, SOX2, SOX4, SOX8, SOX9, SP1, SP3, SPAG9, SPARC, SPDEF, SPHK1, SPINK1, SPINT1, SPINT2, SPN, SPOCK1, SPON2, SPP1, SQLE, SRA1, SRC, SRF, SRGN, SRPK1, SRR, SRXN1, SS18, SSR4, SSTR2, SSX2IP, ST14, ST3GAL1, ST6GAL1, ST6GALNAC2, ST8SIA1, STARD13, STAT1, STAT3, STAT5A, STAT5B, STAT6, STC1, STC2, STIM1, STK11, STMN1, STOML2, STUB1, STX2, STX6, STYK1, SULF2, SUV39H1, SUZ12, SYK, SYNJ2, SYNPO2, SYTL1, SYTL2, TAC1, TACC3, TACR1, TACSTD2, TAGLN, TAP1, TAZ, TBK1, TBL1XR1, TBX2, TBX21, TBX3, TBXAS1, TCF12, TCF3, TCF7L2, TEK, TES, TET1, TET2, TFAP4, TFCP2, TFF1, TFF2, TFF3, TFPI2, TG, TGFA, TGFB1, TGFB2, TGFBI, TGFBR1, TGFBR2,

TGFBR3, TGIF2, TGM2, THBS1, THBS2, THPO, THRB, THY1, TIAM1, TIMP1, TIMP2, TIMP3, TJP1, TLR2, TLR3, TLR4, TM4SF1, TM4SF5, TMBIM1, TMBIM4, TMBIM6, TMEM100, TMEM88, TMEM8B, TMEM97, TMPRSS2, TMPRSS4, TMSB15A, TMSB4X, TNC, TNF, TNFAIP1, TNFAIP2, TNFAIP3, TNFAIP8, TNFAIP8L2, TNFRSF10A, TNFRSF10B, TNFRSF11A, TNFRSF11B, TNFRSF12A, TNFRSF1A, TNFRSF1B, TNFRSF25, TNFRSF4, TNFSF11, TNFSF12, TNFSF13, TNK2, TNN, TNS1, TNS2, TNS3, TNS4, TOP2A, TP53, TP63, TP73, TPBG, TPD52, TPM1, TPM2, TPM3, TPP1, TPST1, TPX2, TRAF4, TRAF6, TRAP1, TRIM16, TRIM25, TRIM28, TRIM29, TRIM32, TRIM33, TRIP10, TRIT1, TRPC1, TRPM7, TRPS1, TRPV6, TSC2, TSG101, TSLP, TSPAN12, TSPAN2, TSPAN7, TSPAN8, TTC36, TTC9, TTF2, TUBB4B, TUSC3, TWIST1, TWIST2, TXN, TXNDC12, TXNIP, TYK2, TYMP, TYMS, TYRP1, UBA2, UBD, UBE2C, UBE2I, UBE2N, UBE2V1, UCHL1, UCP2, UGCG, UGT2B15, UGT2B17, UHRF1, UIMC1, ULK1, UMPS, UNC45A, UNC5B, UPF1, UPP1, USP14, USP22, USP28, UTRN, VANGL1, VASH1, VASP, VAV2, VAV3, VCAM1, VCAN, VCP, VDR, VEGFA, VEGFB, VEGFC, VEZT, VIM, VLDLR, VMP1, VSNL1, VTCN1, VWCE, WASF1, WASF2, WASF3, WASL, WFDC2, WIF1, WISP1, WISP2, WISP3, WLS, WNT11, WNT2B, WNT5A, WNT7B, WRAP53, WSB1, WT1, WWOX, WWP1, WWTR1, XCR1, XIAP, XRCC1, XRCC2, XRCC5, YAP1, YBX1, YES1, YWHAE, YWHAG, YWHAZ, YY1, ZBTB16, ZBTB20, ZBTB33, ZBTB7A, ZDHHC2, ZEB1, ZEB2, ZFHX3, ZFP36, ZFX, ZHX2, ZNF148, ZNF217, ZNF350, ZNF382, ZNF592, ZNF703.

Table S3. 246 DE-MTGs identified in PDAC

Gene	logFC	P Value	FDR	regulated
LAMC2	2.650	1.51583E-33	2.28981E-29	Up-Regulated
S100P	4.297	3.48635E-33	2.63324E-29	Up-Regulated
KRT19	3.527	7.83878E-33	3.94709E-29	Up-Regulated
ITGA2	2.048	2.51479E-32	7.59769E-29	Up-Regulated
SLPI	2.634	1.12073E-31	2.82161E-28	Up-Regulated
ANXA2	1.298	2.99506E-31	6.46334E-28	Up-Regulated
INHBA	3.142	2.69629E-30	5.09126E-27	Up-Regulated
GJB2	3.389	3.20932E-30	5.38667E-27	Up-Regulated
FN1	2.787	1.02154E-29	1.54314E-26	Up-Regulated
KRT7	2.681	2.05178E-29	2.58285E-26	Up-Regulated
SFN	2.944	6.54358E-29	7.06052E-26	Up-Regulated
LAMB3	2.223	3.55549E-28	2.98384E-25	Up-Regulated
ANO1	1.826	6.90453E-28	5.21499E-25	Up-Regulated
SERPINB5	2.598	1.64594E-27	1.18398E-24	Up-Regulated
S100A11	1.839	1.89543E-27	1.30147E-24	Up-Regulated
IGFBP3	2.459	2.09147E-27	1.37364E-24	Up-Regulated
S100A10	1.439	3.54929E-27	2.23398E-24	Up-Regulated
TMPRSS4	2.301	7.61661E-27	4.10916E-24	Up-Regulated
CTHRC1	3.221	3.35714E-26	1.44894E-23	Up-Regulated
S100A6	2.392	3.48733E-26	1.46332E-23	Up-Regulated
CAPG	1.813	7.40686E-26	3.024E-23	Up-Regulated
SDC1	1.422	8.08522E-26	3.13167E-23	Up-Regulated
HOXB7	1.497	2.20995E-25	7.76362E-23	Up-Regulated
TPBG	1.330	2.32188E-25	7.97144E-23	Up-Regulated
FHL2	1.802	3.28089E-25	1.07741E-22	Up-Regulated
THBS2	2.681	2.2053E-24	5.94881E-22	Up-Regulated
SOX4	1.139	2.48598E-24	6.5374E-22	Up-Regulated
POSTN	3.309	3.04284E-24	7.53528E-22	Up-Regulated
NET1	1.170	4.25854E-24	1.0211E-21	Up-Regulated
MSLN	2.889	4.34982E-24	1.02669E-21	Up-Regulated
FXYD3	1.948	4.89832E-24	1.12112E-21	Up-Regulated
DKK1	2.605	6.63757E-24	1.49652E-21	Up-Regulated
CEACAM6	3.786	9.25046E-24	1.96813E-21	Up-Regulated
CD9	1.272	1.16825E-23	2.44678E-21	Up-Regulated
LGALS3	1.412	1.35063E-23	2.75712E-21	Up-Regulated
ITGA3	1.502	1.63418E-23	3.29145E-21	Up-Regulated
PKM	1.358	7.1455E-23	1.31634E-20	Up-Regulated
FERMT1	1.786	7.94031E-23	1.44514E-20	Up-Regulated
TIMP1	2.013	1.02281E-22	1.83936E-20	Up-Regulated
CEACAM5	4.088	1.13206E-22	2.01187E-20	Up-Regulated
NMU	2.293	1.53317E-22	2.54506E-20	Up-Regulated
CDH3	1.672	1.782E-22	2.89738E-20	Up-Regulated
KRT17	2.403	1.78377E-22	2.89738E-20	Up-Regulated
CD55	1.902	1.90417E-22	3.06005E-20	Up-Regulated
HK2	1.817	2.12681E-22	3.38186E-20	Up-Regulated
VCAN	2.583	2.62213E-22	4.12603E-20	Up-Regulated
ISG15	1.789	4.9303E-22	7.30167E-20	Up-Regulated
LCN2	3.038	5.72284E-22	8.31242E-20	Up-Regulated
SDC4	1.179	6.95445E-22	9.81813E-20	Up-Regulated
LGALS3BP	1.471	9.72522E-22	1.33554E-19	Up-Regulated

SAMD9	1.562	9.8573E-22	1.34148E-19	Up-Regulated
KCNN4	1.514	1.09322E-21	1.47448E-19	Up-Regulated
SLC2A1	1.558	1.6045E-21	2.0198E-19	Up-Regulated
EFNB2	1.717	2.37555E-21	2.91748E-19	Up-Regulated
SMYD3	1.028	2.67288E-21	3.20448E-19	Up-Regulated
ECT2	1.224	4.30037E-21	4.99703E-19	Up-Regulated
AHR	1.388	5.47428E-21	6.18614E-19	Up-Regulated
TM4SF1	1.513	5.81239E-21	6.45603E-19	Up-Regulated
SYTL2	1.578	5.98494E-21	6.59917E-19	Up-Regulated
COL3A1	2.181	6.32843E-21	6.92733E-19	Up-Regulated
FZD2	1.138	9.1288E-21	9.71124E-19	Up-Regulated
SULF2	1.863	2.44863E-20	2.40188E-18	Up-Regulated
MST1R	1.477	2.56504E-20	2.48382E-18	Up-Regulated
SPON2	1.328	2.7176E-20	2.61478E-18	Up-Regulated
JUP	1.270	3.7489E-20	3.56169E-18	Up-Regulated
AGR2	2.754	3.91238E-20	3.67083E-18	Up-Regulated
INPP4B	1.572	4.11524E-20	3.81379E-18	Up-Regulated
LTBP1	1.244	4.74966E-20	4.34839E-18	Up-Regulated
PLAUR	1.502	6.62283E-20	5.81654E-18	Up-Regulated
KLF5	2.085	7.29281E-20	6.33133E-18	Up-Regulated
CAP1	1.128	7.81989E-20	6.75013E-18	Up-Regulated
DKK3	1.346	1.94999E-19	1.52625E-17	Up-Regulated
SPARC	1.835	2.12078E-19	1.62573E-17	Up-Regulated
ANXA3	1.651	2.34191E-19	1.76884E-17	Up-Regulated
FXYD5	1.535	2.47063E-19	1.84759E-17	Up-Regulated
PLAU	1.695	2.75283E-19	2.03844E-17	Up-Regulated
FOXQ1	2.130	3.01042E-19	2.18632E-17	Up-Regulated
PRKCI	1.072	4.09814E-19	2.87937E-17	Up-Regulated
SPOCK1	1.585	4.15053E-19	2.90268E-17	Up-Regulated
RACGAP1	1.419	4.56045E-19	3.1601E-17	Up-Regulated
ENO2	1.475	6.4911E-19	4.43686E-17	Up-Regulated
FAP	2.128	7.56228E-19	5.09981E-17	Up-Regulated
STYK1	1.455	1.03591E-18	6.80369E-17	Up-Regulated
PALLD	1.644	1.92738E-18	1.18354E-16	Up-Regulated
MMP28	1.264	1.97516E-18	1.20262E-16	Up-Regulated
S100A4	1.891	2.18637E-18	1.30029E-16	Up-Regulated
ECM1	1.293	4.17247E-18	2.30877E-16	Up-Regulated
LIPH	1.393	4.28271E-18	2.36112E-16	Up-Regulated
NUAK1	1.308	4.40085E-18	2.39997E-16	Up-Regulated
LIMS1	1.056	4.94723E-18	2.6501E-16	Up-Regulated
EPS8	1.214	5.0295E-18	2.67995E-16	Up-Regulated
WISP1	1.376	6.3148E-18	3.21183E-16	Up-Regulated
SLC16A4	1.625	7.102E-18	3.58806E-16	Up-Regulated
WNT5A	1.291	1.23628E-17	5.83603E-16	Up-Regulated
TGFBI	1.478	1.26796E-17	5.94838E-16	Up-Regulated
BGN	1.586	1.59439E-17	7.25449E-16	Up-Regulated
MMP7	2.582	2.11277E-17	9.35939E-16	Up-Regulated
MX1	1.383	2.36066E-17	1.03362E-15	Up-Regulated
LDHA	1.038	2.41231E-17	1.05015E-15	Up-Regulated
SQLE	1.101	3.96162E-17	1.65316E-15	Up-Regulated
TOP2A	2.074	5.65143E-17	2.29491E-15	Up-Regulated
CORO1C	1.165	5.99609E-17	2.41539E-15	Up-Regulated

THY1	1.339	6.28914E-17	2.52E-15	Up-Regulated
MMP11	1.411	6.40908E-17	2.5545E-15	Up-Regulated
CLIC1	1.023	6.58877E-17	2.61233E-15	Up-Regulated
CTSB	1.060	6.75911E-17	2.65894E-15	Up-Regulated
OLR1	2.105	8.03876E-17	3.07427E-15	Up-Regulated
TFF1	3.213	8.83286E-17	3.32741E-15	Up-Regulated
MMP1	3.116	8.92279E-17	3.35293E-15	Up-Regulated
CDH11	1.338	1.03343E-16	3.81742E-15	Up-Regulated
KIF14	1.181	1.48625E-16	5.30763E-15	Up-Regulated
YWHAZ	1.015	1.9324E-16	6.72598E-15	Up-Regulated
CMTM3	1.005	2.07359E-16	7.15152E-15	Up-Regulated
EDNRA	1.782	3.34831E-16	1.0716E-14	Up-Regulated
NDC80	1.431	4.33743E-16	1.34817E-14	Up-Regulated
CTSK	1.657	4.52339E-16	1.39166E-14	Up-Regulated
LGALS1	1.589	5.93138E-16	1.77777E-14	Up-Regulated
TRIM29	1.230	6.16585E-16	1.83727E-14	Up-Regulated
TXN	1.012	6.17749E-16	1.83727E-14	Up-Regulated
ANXA1	1.519	6.74158E-16	1.98129E-14	Up-Regulated
HK1	1.091	7.64906E-16	2.20509E-14	Up-Regulated
ADAM9	1.407	1.02266E-15	2.86081E-14	Up-Regulated
GBP2	1.186	1.26479E-15	3.4425E-14	Up-Regulated
VASP	1.011	1.48913E-15	3.95339E-14	Up-Regulated
CXCL8	2.250	2.06172E-15	5.29666E-14	Up-Regulated
TNFAIP2	1.051	2.36292E-15	5.98897E-14	Up-Regulated
DPYSL3	1.416	2.3915E-15	6.04113E-14	Up-Regulated
MIA	1.458	3.27425E-15	7.92641E-14	Up-Regulated
MDK	1.407	4.37578E-15	1.03121E-13	Up-Regulated
TWIST1	1.532	4.87368E-15	1.13014E-13	Up-Regulated
ITGB6	1.716	5.35752E-15	1.23558E-13	Up-Regulated
MMP2	1.571	5.74944E-15	1.31393E-13	Up-Regulated
EPHA2	1.110	7.12237E-15	1.60344E-13	Up-Regulated
EPSTI1	1.169	7.36535E-15	1.64344E-13	Up-Regulated
PLOD2	1.068	1.07931E-14	2.30609E-13	Up-Regulated
RARRES3	1.444	1.19768E-14	2.52453E-13	Up-Regulated
CENPW	1.121	1.21959E-14	2.5659E-13	Up-Regulated
S100A2	1.904	1.83378E-14	3.73331E-13	Up-Regulated
CCNB1	1.303	2.32886E-14	4.59657E-13	Up-Regulated
F2R	1.090	3.49847E-14	6.64753E-13	Up-Regulated
MACC1	1.086	4.00144E-14	7.50878E-13	Up-Regulated
ITGB1	1.152	4.48617E-14	8.32531E-13	Up-Regulated
F3	1.054	4.80561E-14	8.84209E-13	Up-Regulated
EGLN3	1.353	4.87081E-14	8.91008E-13	Up-Regulated
PDGFRB	1.062	6.3265E-14	1.11255E-12	Up-Regulated
ASPN	1.422	8.02843E-14	1.37347E-12	Up-Regulated
S100A14	1.276	8.35303E-14	1.41936E-12	Up-Regulated
IL18	1.261	8.87455E-14	1.49619E-12	Up-Regulated
CLDN18	2.542	1.06104E-13	1.75554E-12	Up-Regulated
COL1A1	1.345	1.15758E-13	1.89452E-12	Up-Regulated
KRT8	1.419	1.16049E-13	1.89723E-12	Up-Regulated
LIF	1.126	1.23973E-13	2.01585E-12	Up-Regulated
CLDN1	1.419	1.68431E-13	2.64207E-12	Up-Regulated
TIMP2	1.062	2.05992E-13	3.13998E-12	Up-Regulated

FBN1	1.455	2.46313E-13	3.70968E-12	Up-Regulated
MXD1	1.308	2.61033E-13	3.90026E-12	Up-Regulated
LIMA1	1.291	2.92145E-13	4.29712E-12	Up-Regulated
CXCL3	1.599	3.02208E-13	4.4279E-12	Up-Regulated
PTPRR	1.226	3.68252E-13	5.27281E-12	Up-Regulated
FSCN1	1.005	5.74227E-13	7.84292E-12	Up-Regulated
GSDMB	1.077	6.62235E-13	8.90803E-12	Up-Regulated
PTTG1	1.089	7.25558E-13	9.63118E-12	Up-Regulated
AURKA	1.088	9.48464E-13	1.21729E-11	Up-Regulated
FZD7	1.105	9.92186E-13	1.26695E-11	Up-Regulated
RAB25	1.549	1.22381E-12	1.51905E-11	Up-Regulated
ROBO1	1.270	1.30972E-12	1.60982E-11	Up-Regulated
TNFRSF11B	1.205	1.73798E-12	2.06561E-11	Up-Regulated
CSTA	1.561	1.89723E-12	2.23554E-11	Up-Regulated
ACTA2	1.317	1.90984E-12	2.24864E-11	Up-Regulated
TPX2	1.051	2.8211E-12	3.19452E-11	Up-Regulated
PSCA	1.891	3.70686E-12	4.06061E-11	Up-Regulated
ADAM12	1.365	4.24067E-12	4.57242E-11	Up-Regulated
DUSP6	1.077	4.93668E-12	5.22956E-11	Up-Regulated
ID1	1.385	5.06156E-12	5.34311E-11	Up-Regulated
CEMIP	1.332	6.33802E-12	6.47782E-11	Up-Regulated
MUC20	1.023	6.76482E-12	6.86757E-11	Up-Regulated
ADAM28	1.061	9.40456E-12	9.21305E-11	Up-Regulated
EZR	1.032	1.01101E-11	9.78996E-11	Up-Regulated
IFITM1	1.031	1.29914E-11	1.21893E-10	Up-Regulated
MSN	1.045	1.44262E-11	1.33123E-10	Up-Regulated
CLIC3	1.289	1.48269E-11	1.35955E-10	Up-Regulated
IRF7	1.004	1.70301E-11	1.53038E-10	Up-Regulated
PPARG	1.023	1.81673E-11	1.62196E-10	Up-Regulated
SFRP2	1.815	3.43849E-11	2.86338E-10	Up-Regulated
MMP12	2.147	4.71547E-11	3.81327E-10	Up-Regulated
GPR87	1.353	5.02891E-11	4.04078E-10	Up-Regulated
PRRX1	1.203	6.87445E-11	5.32608E-10	Up-Regulated
KLK7	1.313	7.95897E-11	6.06907E-10	Up-Regulated
SPP1	1.573	1.29766E-10	9.35231E-10	Up-Regulated
GPNMB	1.310	1.45843E-10	1.03969E-09	Up-Regulated
KRT18	1.122	1.69825E-10	1.19098E-09	Up-Regulated
TSPAN8	1.851	1.70566E-10	1.19563E-09	Up-Regulated
LOX	1.406	1.94504E-10	1.34655E-09	Up-Regulated
TAGLN	1.202	2.75256E-10	1.84473E-09	Up-Regulated
MUC16	1.409	4.47633E-10	2.84474E-09	Up-Regulated
GJA1	1.110	4.49878E-10	2.8566E-09	Up-Regulated
SERPINB3	1.566	6.17616E-10	3.7941E-09	Up-Regulated
THBS1	1.270	7.48846E-10	4.53026E-09	Up-Regulated
BIRC3	1.037	7.49465E-10	4.53219E-09	Up-Regulated
PLA2G10	1.041	1.05918E-09	6.22326E-09	Up-Regulated
GALNT3	1.049	1.07759E-09	6.32161E-09	Up-Regulated
AGR3	1.959	1.16719E-09	6.78397E-09	Up-Regulated
PROM1	1.636	1.22819E-09	7.10029E-09	Up-Regulated
SOX9	1.133	1.36763E-09	7.82556E-09	Up-Regulated
CYP3A5	1.519	1.69432E-09	9.51112E-09	Up-Regulated
CXCL14	1.494	1.73761E-09	9.72161E-09	Up-Regulated

SNAI2	1.096	1.88E-09	1.04179E-08	Up-Regulated
KLK6	1.078	4.11824E-09	2.11887E-08	Up-Regulated
MUC1	1.410	5.01873E-09	2.53385E-08	Up-Regulated
PMP22	1.033	5.20828E-09	2.62429E-08	Up-Regulated
ODAM	1.256	5.26415E-09	2.65042E-08	Up-Regulated
AKR1C3	1.209	1.44312E-08	6.65218E-08	Up-Regulated
CST6	1.077	2.12425E-08	9.43236E-08	Up-Regulated
IL7	1.036	2.25141E-08	9.93857E-08	Up-Regulated
CYP1B1	1.448	2.32558E-08	1.02212E-07	Up-Regulated
MUC13	1.198	3.38845E-08	1.44024E-07	Up-Regulated
TFF2	1.942	4.61486E-08	1.90835E-07	Up-Regulated
CCL18	1.263	9.68537E-08	3.75435E-07	Up-Regulated
PTGS2	1.334	2.87499E-07	1.02043E-06	Up-Regulated
REG4	2.122	5.30651E-07	1.79691E-06	Up-Regulated
CXCR4	1.169	5.3842E-07	1.8224E-06	Up-Regulated
MMP10	1.113	5.96684E-07	2.00078E-06	Up-Regulated
TFF3	1.248	7.11225E-07	2.3549E-06	Up-Regulated
CHI3L1	1.135	8.01269E-07	2.62616E-06	Up-Regulated
IGFBP1	1.032	2.41993E-06	7.2936E-06	Up-Regulated
EPCAM	1.121	1.09428E-05	2.93302E-05	Up-Regulated
CDH17	1.044	0.0002552	0.000529546	Up-Regulated
PDCD4	-1.060	2.46429E-19	1.84759E-17	Down-Regulated
CTNND2	-1.372	7.14833E-15	1.6045E-13	Down-Regulated
LIFR	-1.129	1.14751E-14	2.44146E-13	Down-Regulated
LGALS2	-1.847	1.55359E-14	3.18866E-13	Down-Regulated
NUCB2	-1.276	5.52708E-13	7.57642E-12	Down-Regulated
BNIP3	-1.512	2.18662E-12	2.52918E-11	Down-Regulated
PRDX4	-1.117	1.13658E-11	1.07847E-10	Down-Regulated
AQP8	-2.859	1.3214E-11	1.2363E-10	Down-Regulated
EGF	-2.062	1.46148E-11	1.34371E-10	Down-Regulated
PLCE1	-1.272	2.8532E-11	2.42819E-10	Down-Regulated
DPEP1	-1.261	9.81142E-11	7.28311E-10	Down-Regulated
CXCL12	-1.111	2.186E-10	1.50509E-09	Down-Regulated
TSPAN7	-1.059	5.02373E-10	3.15477E-09	Down-Regulated
RNASE1	-1.188	1.47475E-09	8.38136E-09	Down-Regulated
TMEM97	-1.307	3.63873E-07	1.26689E-06	Down-Regulated
IGFBP2	-1.565	1.31278E-06	4.14785E-06	Down-Regulated
LYVE1	-1.014	1.83383E-05	4.70719E-05	Down-Regulated
PRSS3	-1.765	1.86821E-05	4.78974E-05	Down-Regulated
FAM3B	-1.154	0.00016175	0.000348669	Down-Regulated

Table S4. Enrichment analysis

Category	Term	Count	%	PValue
GOTERM_BP_DIRECT	GO:0007155~cell adhesion	39	15.85365854	8.72103E-19
GOTERM_BP_DIRECT	GO:0022617~extracellular matrix disassembly	16	6.504065041	1.58595E-13
GOTERM_BP_DIRECT	GO:0030198~extracellular matrix organization	22	8.943089431	6.15769E-13
GOTERM_BP_DIRECT	GO:0042060~wound healing	13	5.284552846	1.28156E-09
GOTERM_BP_DIRECT	GO:0007165~signal transduction	45	18.29268293	1.68451E-09
GOTERM_BP_DIRECT	GO:0030574~collagen catabolic process	11	4.471544715	2.09882E-08
GOTERM_BP_DIRECT	GO:0042493~response to drug	20	8.130081301	7.09106E-08
GOTERM_BP_DIRECT	GO:0008284~positive regulation of cell proliferation	23	9.349593496	8.58517E-07
GOTERM_BP_DIRECT	GO:0001666~response to hypoxia	14	5.691056911	1.06001E-06
GOTERM_BP_DIRECT	GO:0016477~cell migration	14	5.691056911	1.06001E-06
GOTERM_BP_DIRECT	GO:0043066~negative regulation of apoptotic process	22	8.943089431	2.1985E-06
GOTERM_BP_DIRECT	GO:0043434~response to peptide hormone	8	3.25203252	2.57906E-06
GOTERM_BP_DIRECT	GO:0001525~angiogenesis	15	6.097560976	3.6781E-06
GOTERM_BP_DIRECT	GO:0006954~inflammatory response	19	7.723577236	8.06111E-06
GOTERM_BP_DIRECT	GO:0030199~collagen fibril organization	7	2.845528455	1.67874E-05
GOTERM_BP_DIRECT	GO:0001649~osteoblast differentiation	10	4.06504065	1.69349E-05
GOTERM_BP_DIRECT	GO:0001558~regulation of cell growth	9	3.658536585	1.74324E-05
GOTERM_BP_DIRECT	GO:0009612~response to mechanical stimulus	8	3.25203252	1.91681E-05
GOTERM_BP_DIRECT	GO:0006928~movement of cell or subcellular component	9	3.658536585	2.96656E-05
GOTERM_BP_DIRECT	GO:0098609~cell-cell adhesion	15	6.097560976	3.37603E-05
GOTERM_BP_DIRECT	GO:0045766~positive regulation of angiogenesis	10	4.06504065	3.78804E-05
GOTERM_BP_DIRECT	GO:0033627~cell adhesion mediated by integrin	5	2.032520325	4.75038E-05
GOTERM_BP_DIRECT	GO:0090004~positive regulation of establishment of protein localization to plasma membrane	6	2.43902439	5.85099E-05
GOTERM_BP_DIRECT	GO:0007507~heart development	12	4.87804878	6.11959E-05
GOTERM_BP_DIRECT	GO:0030335~positive regulation of cell migration	12	4.87804878	6.42846E-05
GOTERM_BP_DIRECT	GO:0010628~positive regulation of gene expression	14	5.691056911	9.70207E-05
GOTERM_BP_DIRECT	GO:0046718~viral entry into host cell	8	3.25203252	0.000138736
GOTERM_BP_DIRECT	GO:0001501~skeletal system development	10	4.06504065	0.000147454
GOTERM_BP_DIRECT	GO:0046697~decidualization	5	2.032520325	0.000159469
GOTERM_BP_DIRECT	GO:0008285~negative regulation of cell proliferation	17	6.910569106	0.000174741
GOTERM_BP_DIRECT	GO:0060070~canonical Wnt signaling pathway	8	3.25203252	0.000174929
GOTERM_BP_DIRECT	GO:0009611~response to wounding	7	2.845528455	0.000263354
GOTERM_BP_DIRECT	GO:0007160~cell-matrix adhesion	8	3.25203252	0.000289501
GOTERM_BP_DIRECT	GO:0051384~response to glucocorticoid	7	2.845528455	0.000312703
GOTERM_BP_DIRECT	GO:0048333~mesodermal cell differentiation	4	1.62601626	0.000426492
GOTERM_BP_DIRECT	GO:0071260~cellular response to mechanical stimulus	7	2.845528455	0.000505268
GOTERM_BP_DIRECT	GO:0007229~integrin-mediated signaling pathway	8	3.25203252	0.000518052
GOTERM_BP_DIRECT	GO:0071560~cellular response to transforming growth factor beta stimulus	6	2.43902439	0.000629074
GOTERM_BP_DIRECT	GO:0007584~response to nutrient	7	2.845528455	0.000630928
GOTERM_BP_DIRECT	GO:0002576~platelet degranulation	8	3.25203252	0.00065736
GOTERM_BP_DIRECT	GO:0043542~endothelial cell migration	5	2.032520325	0.00070732

GOTERM_BP_DIRECT	GO:0006508~proteolysis	18	7.317073171	0.000806691
GOTERM_BP_DIRECT	GO:0051017~actin filament bundle assembly	5	2.032520325	0.000807137
GOTERM_BP_DIRECT	GO:0010812~negative regulation of cell-substrate adhesion	4	1.62601626	0.00091172
GOTERM_BP_DIRECT	GO:0001503~ossification	7	2.845528455	0.000953886
GOTERM_BP_DIRECT	GO:0009615~response to virus	8	3.25203252	0.000971271
GOTERM_BP_DIRECT	GO:0048146~positive regulation of fibroblast proliferation	6	2.43902439	0.000985148
GOTERM_BP_DIRECT	GO:0007267~cell-cell signaling	12	4.87804878	0.001026738
GOTERM_BP_DIRECT	GO:0043065~positive regulation of apoptotic process	13	5.284552846	0.001225944
GOTERM_BP_DIRECT	GO:0051897~positive regulation of protein kinase B signaling	7	2.845528455	0.00123123
GOTERM_BP_DIRECT	GO:0070301~cellular response to hydrogen peroxide	6	2.43902439	0.001260256
GOTERM_BP_DIRECT	GO:0042127~regulation of cell proliferation	10	4.06504065	0.001299008
GOTERM_BP_DIRECT	GO:0001837~epithelial to mesenchymal transition	5	2.032520325	0.001306671
GOTERM_BP_DIRECT	GO:0051216~cartilage development	6	2.43902439	0.001472745
GOTERM_BP_DIRECT	GO:0010951~negative regulation of endopeptidase activity	8	3.25203252	0.001690816
GOTERM_BP_DIRECT	GO:0006935~chemotaxis	8	3.25203252	0.001772551
GOTERM_BP_DIRECT	GO:0050900~leukocyte migration	8	3.25203252	0.001772551
GOTERM_BP_DIRECT	GO:0032355~response to estradiol	7	2.845528455	0.001860252
GOTERM_BP_DIRECT	GO:0009653~anatomical structure morphogenesis	7	2.845528455	0.001966914
GOTERM_BP_DIRECT	GO:0043627~response to estrogen	6	2.43902439	0.002268786
GOTERM_BP_DIRECT	GO:0090090~negative regulation of canonical Wnt signaling pathway	9	3.658536585	0.002270892
GOTERM_BP_DIRECT	GO:0030336~negative regulation of cell migration	7	2.845528455	0.002314772
GOTERM_BP_DIRECT	GO:0045184~establishment of protein localization	5	2.032520325	0.002409752
GOTERM_BP_DIRECT	GO:0006952~defense response	6	2.43902439	0.002592844
GOTERM_BP_DIRECT	GO:0001957~intramembranous ossification	3	1.219512195	0.002890084
GOTERM_BP_DIRECT	GO:0045987~positive regulation of smooth muscle contraction	4	1.62601626	0.00309622
GOTERM_BP_DIRECT	GO:0030855~epithelial cell differentiation	6	2.43902439	0.003140075
GOTERM_BP_DIRECT	GO:0071300~cellular response to retinoic acid	6	2.43902439	0.003140075
GOTERM_BP_DIRECT	GO:0030155~regulation of cell adhesion	5	2.032520325	0.003148148
GOTERM_BP_DIRECT	GO:0070098~chemokine-mediated signaling pathway	6	2.43902439	0.003339681
GOTERM_BP_DIRECT	GO:0090103~cochlea morphogenesis	4	1.62601626	0.003547918
GOTERM_BP_DIRECT	GO:0043567~regulation of insulin-like growth factor receptor signaling pathway	3	1.219512195	0.004008375
GOTERM_BP_DIRECT	GO:0000187~activation of MAPK activity	7	2.845528455	0.004184996
GOTERM_BP_DIRECT	GO:0030216~keratinocyte differentiation	6	2.43902439	0.004476692
GOTERM_BP_DIRECT	GO:0071356~cellular response to tumor necrosis factor	7	2.845528455	0.004789709
GOTERM_BP_DIRECT	GO:0033629~negative regulation of cell adhesion mediated by integrin	3	1.219512195	0.005294705
GOTERM_BP_DIRECT	GO:0060054~positive regulation of epithelial cell proliferation involved in wound healing	3	1.219512195	0.005294705
GOTERM_BP_DIRECT	GO:0018149~peptide cross-linking	5	2.032520325	0.005439413
GOTERM_BP_DIRECT	GO:0061621~canonical glycolysis	4	1.62601626	0.005745781
GOTERM_BP_DIRECT	GO:0035025~positive regulation of Rho protein signal transduction	4	1.62601626	0.005745781
GOTERM_BP_DIRECT	GO:0042542~response to hydrogen peroxide	5	2.032520325	0.005837885
GOTERM_BP_DIRECT	GO:0034097~response to cytokine	5	2.032520325	0.006255344
GOTERM_BP_DIRECT	GO:0010800~positive regulation of peptidyl-threonine phosphorylation	4	1.62601626	0.006397206
GOTERM_BP_DIRECT	GO:0035987~endodermal cell differentiation	4	1.62601626	0.006397206
GOTERM_BP_DIRECT	GO:0044267~cellular protein metabolic process	7	2.845528455	0.006714821

GOTERM_BP_DIRECT	GO:0045198~establishment of epithelial cell apical/basal polarity	3	1.219512195	0.006744126
GOTERM_BP_DIRECT	GO:0006915~apoptotic process	17	6.910569106	0.007069602
GOTERM_BP_DIRECT	GO:0043388~positive regulation of DNA binding	4	1.62601626	0.007090884
GOTERM_BP_DIRECT	GO:0007411~axon guidance	8	3.25203252	0.007542748
GOTERM_BP_DIRECT	GO:0060548~negative regulation of cell death	5	2.032520325	0.007625391
GOTERM_BP_DIRECT	GO:0030203~glycosaminoglycan metabolic process	4	1.62601626	0.007827449
GOTERM_BP_DIRECT	GO:0040037~negative regulation of fibroblast growth factor receptor signaling pathway	3	1.219512195	0.008351794
GOTERM_BP_DIRECT	GO:0071803~positive regulation of podosome assembly	3	1.219512195	0.008351794
GOTERM_BP_DIRECT	GO:0044344~cellular response to fibroblast growth factor stimulus	4	1.62601626	0.008607483
GOTERM_BP_DIRECT	GO:0071407~cellular response to organic cyclic compound	5	2.032520325	0.009739928
GOTERM_BP_DIRECT	GO:0016266~O-glycan processing	5	2.032520325	0.010322184
GOTERM_BP_DIRECT	GO:0048661~positive regulation of smooth muscle cell proliferation	5	2.032520325	0.010322184
GOTERM_BP_DIRECT	GO:0045669~positive regulation of osteoblast differentiation	5	2.032520325	0.010322184
GOTERM_BP_DIRECT	GO:0008584~male gonad development	6	2.43902439	0.010845629
GOTERM_BP_DIRECT	GO:0015758~glucose transport	4	1.62601626	0.011213457
GOTERM_BP_DIRECT	GO:0010634~positive regulation of epithelial cell migration	4	1.62601626	0.011213457
GOTERM_BP_DIRECT	GO:0071364~cellular response to epidermal growth factor stimulus	4	1.62601626	0.011213457
GOTERM_BP_DIRECT	GO:0006977~DNA damage response, signal transduction by p53 class mediator resulting in cell cycle arrest	5	2.032520325	0.01155321
GOTERM_BP_DIRECT	GO:0090179~planar cell polarity pathway involved in neural tube closure	3	1.219512195	0.012023024
GOTERM_BP_DIRECT	GO:0035413~positive regulation of catenin import into nucleus	3	1.219512195	0.012023024
GOTERM_BP_DIRECT	GO:0006096~glycolytic process	4	1.62601626	0.012172178
GOTERM_BP_DIRECT	GO:0007586~digestion	5	2.032520325	0.012202562
GOTERM_BP_DIRECT	GO:0060337~type I interferon signaling pathway	5	2.032520325	0.012874845
GOTERM_BP_DIRECT	GO:0014032~neural crest cell development	3	1.219512195	0.01407741
GOTERM_BP_DIRECT	GO:0016337~single organismal cell-cell adhesion	6	2.43902439	0.014461836
GOTERM_BP_DIRECT	GO:0034332~adherens junction organization	4	1.62601626	0.015323296
GOTERM_BP_DIRECT	GO:0045893~positive regulation of transcription, DNA-templated	15	6.097560976	0.015324923
GOTERM_BP_DIRECT	GO:1902230~negative regulation of intrinsic apoptotic signaling pathway in response to DNA damage	3	1.219512195	0.016271691
GOTERM_BP_DIRECT	GO:0043508~negative regulation of JUN kinase activity	3	1.219512195	0.016271691
GOTERM_BP_DIRECT	GO:2000353~positive regulation of endothelial cell apoptotic process	3	1.219512195	0.016271691
GOTERM_BP_DIRECT	GO:0010839~negative regulation of keratinocyte proliferation	3	1.219512195	0.016271691
GOTERM_BP_DIRECT	GO:0033138~positive regulation of peptidyl-serine phosphorylation	5	2.032520325	0.017404267
GOTERM_BP_DIRECT	GO:0071347~cellular response to interleukin-1	5	2.032520325	0.018243944
GOTERM_BP_DIRECT	GO:0002042~cell migration involved in sprouting angiogenesis	3	1.219512195	0.018601525
GOTERM_BP_DIRECT	GO:0006821~chloride transport	4	1.62601626	0.018891761
GOTERM_BP_DIRECT	GO:0045071~negative regulation of viral genome replication	4	1.62601626	0.018891761
GOTERM_BP_DIRECT	GO:0032526~response to retinoic acid	4	1.62601626	0.020174719
GOTERM_BP_DIRECT	GO:0030334~regulation of cell migration	5	2.032520325	0.020912357
GOTERM_BP_DIRECT	GO:0090280~positive regulation of calcium ion import	3	1.219512195	0.021062661
GOTERM_BP_DIRECT	GO:0070848~response to growth factor	3	1.219512195	0.021062661
GOTERM_BP_DIRECT	GO:0007566~embryo implantation	4	1.62601626	0.021504548
GOTERM_BP_DIRECT	GO:0043086~negative regulation of catalytic activity	5	2.032520325	0.021852207
GOTERM_BP_DIRECT	GO:0060021~palate development	5	2.032520325	0.022817514
GOTERM_BP_DIRECT	GO:0030324~lung development	5	2.032520325	0.022817514

GOTERM_BP_DIRECT	GO:0031623~receptor internalization	4	1.62601626	0.022881278
GOTERM_BP_DIRECT	GO:2000811~negative regulation of anoikis	3	1.219512195	0.023650941
GOTERM_BP_DIRECT	GO:0055093~response to hyperoxia	3	1.219512195	0.023650941
GOTERM_BP_DIRECT	GO:0030033~microvillus assembly	3	1.219512195	0.023650941
GOTERM_BP_DIRECT	GO:0050860~negative regulation of T cell receptor signaling pathway	3	1.219512195	0.023650941
GOTERM_BP_DIRECT	GO:0048514~blood vessel morphogenesis	3	1.219512195	0.023650941
GOTERM_BP_DIRECT	GO:0035567~non-canonical Wnt signaling pathway	3	1.219512195	0.023650941
GOTERM_BP_DIRECT	GO:0010466~negative regulation of peptidase activity	3	1.219512195	0.023650941
GOTERM_BP_DIRECT	GO:0010977~negative regulation of neuron projection development	4	1.62601626	0.024304906
GOTERM_BP_DIRECT	GO:0036342~post-anal tail morphogenesis	3	1.219512195	0.0263623
GOTERM_BP_DIRECT	GO:0043123~positive regulation of I-kappaB kinase/NF-kappaB signaling	7	2.845528455	0.027022226
GOTERM_BP_DIRECT	GO:0051591~response to cAMP	4	1.62601626	0.027292695
GOTERM_BP_DIRECT	GO:0010595~positive regulation of endothelial cell migration	4	1.62601626	0.027292695
GOTERM_BP_DIRECT	GO:0043410~positive regulation of MAPK cascade	5	2.032520325	0.028030639
GOTERM_BP_DIRECT	GO:1905049~negative regulation of metallopeptidase activity	2	0.81300813	0.028146778
GOTERM_BP_DIRECT	GO:0097325~melanocyte proliferation	2	0.81300813	0.028146778
GOTERM_BP_DIRECT	GO:0071306~cellular response to vitamin E	2	0.81300813	0.028146778
GOTERM_BP_DIRECT	GO:0030825~positive regulation of cGMP metabolic process	2	0.81300813	0.028146778
GOTERM_BP_DIRECT	GO:0010710~regulation of collagen catabolic process	2	0.81300813	0.028146778
GOTERM_BP_DIRECT	GO:1900020~positive regulation of protein kinase C activity	2	0.81300813	0.028146778
GOTERM_BP_DIRECT	GO:0045600~positive regulation of fat cell differentiation	4	1.62601626	0.028856697
GOTERM_BP_DIRECT	GO:0032956~regulation of actin cytoskeleton organization	4	1.62601626	0.028856697
GOTERM_BP_DIRECT	GO:0031100~organ regeneration	4	1.62601626	0.028856697
GOTERM_BP_DIRECT	GO:0010975~regulation of neuron projection development	3	1.219512195	0.02919276
GOTERM_BP_DIRECT	GO:0042246~tissue regeneration	3	1.219512195	0.02919276
GOTERM_BP_DIRECT	GO:0032496~response to lipopolysaccharide	7	2.845528455	0.029213116
GOTERM_BP_DIRECT	GO:0006919~activation of cysteine-type endopeptidase activity involved in apoptotic process	5	2.032520325	0.030298421
GOTERM_BP_DIRECT	GO:0014911~positive regulation of smooth muscle cell migration	3	1.219512195	0.03213843
GOTERM_BP_DIRECT	GO:0008544~epidermis development	5	2.032520325	0.032671585
GOTERM_BP_DIRECT	GO:0051894~positive regulation of focal adhesion assembly	3	1.219512195	0.035195504
GOTERM_BP_DIRECT	GO:0042730~fibrinolysis	3	1.219512195	0.035195504
GOTERM_BP_DIRECT	GO:0071277~cellular response to calcium ion	4	1.62601626	0.035576961
GOTERM_BP_DIRECT	GO:0006955~immune response	12	4.87804878	0.037670245
GOTERM_BP_DIRECT	GO:0045727~positive regulation of translation	4	1.62601626	0.039212962
GOTERM_BP_DIRECT	GO:0045662~negative regulation of myoblast differentiation	3	1.219512195	0.041629067
GOTERM_BP_DIRECT	GO:0032967~positive regulation of collagen biosynthetic process	3	1.219512195	0.041629067
GOTERM_BP_DIRECT	GO:0042663~regulation of endodermal cell fate specification	2	0.81300813	0.041922899
GOTERM_BP_DIRECT	GO:0006725~cellular aromatic compound metabolic process	2	0.81300813	0.041922899
GOTERM_BP_DIRECT	GO:0035583~sequestering of TGFbeta in extracellular matrix	2	0.81300813	0.041922899
GOTERM_BP_DIRECT	GO:0032487~regulation of Rap protein signal transduction	2	0.81300813	0.041922899
GOTERM_BP_DIRECT	GO:0007204~positive regulation of cytosolic calcium ion concentration	6	2.43902439	0.042129182
GOTERM_BP_DIRECT	GO:0007173~epidermal growth factor receptor signaling pathway	4	1.62601626	0.045006179
GOTERM_BP_DIRECT	GO:0042733~embryonic digit morphogenesis	4	1.62601626	0.045006179
GOTERM_BP_DIRECT	GO:0030182~neuron differentiation	5	2.032520325	0.046130997

GOTERM_BP_DIRECT	GO:0042327~positive regulation of phosphorylation	3	1.219512195	0.048464657
GOTERM_BP_DIRECT	GO:0034612~response to tumor necrosis factor	3	1.219512195	0.048464657
GOTERM_BP_DIRECT	GO:0007159~leukocyte cell-cell adhesion	3	1.219512195	0.048464657
GOTERM_BP_DIRECT	GO:0031214~biomineral tissue development	3	1.219512195	0.048464657
GOTERM_BP_DIRECT	GO:0070373~negative regulation of ERK1 and ERK2 cascade	4	1.62601626	0.049090885
GOTERM_BP_DIRECT	GO:0051592~response to calcium ion	4	1.62601626	0.049090885
GOTERM_BP_DIRECT	GO:0001701~in utero embryonic development	7	2.845528455	0.049890899
GOTERM_CC_DIRECT	GO:0005615~extracellular space	92	37.39837398	2.4068E-41
GOTERM_CC_DIRECT	GO:0070062~extracellular exosome	103	41.8699187	7.81364E-24
GOTERM_CC_DIRECT	GO:0005578~proteinaceous extracellular matrix	30	12.19512195	2.39364E-18
GOTERM_CC_DIRECT	GO:0031012~extracellular matrix	29	11.78861789	3.19423E-16
GOTERM_CC_DIRECT	GO:0005576~extracellular region	65	26.42276423	5.24147E-16
GOTERM_CC_DIRECT	GO:0005925~focal adhesion	30	12.19512195	6.08752E-14
GOTERM_CC_DIRECT	GO:0009986~cell surface	31	12.60162602	3.83878E-11
GOTERM_CC_DIRECT	GO:0016324~apical plasma membrane	20	8.130081301	1.2905E-08
GOTERM_CC_DIRECT	GO:0005913~cell-cell adherens junction	20	8.130081301	6.83331E-08
GOTERM_CC_DIRECT	GO:0031982~vesicle	13	5.284552846	1.395E-07
GOTERM_CC_DIRECT	GO:0005886~plasma membrane	87	35.36585366	2.95405E-06
GOTERM_CC_DIRECT	GO:0031528~microvillus membrane	6	2.43902439	3.95721E-06
GOTERM_CC_DIRECT	GO:0005796~Golgi lumen	10	4.06504065	5.25678E-06
GOTERM_CC_DIRECT	GO:0071944~cell periphery	7	2.845528455	1.16493E-05
GOTERM_CC_DIRECT	GO:0048471~perinuclear region of cytoplasm	23	9.349593496	2.9934E-05
GOTERM_CC_DIRECT	GO:0071438~invadopodium membrane	4	1.62601626	4.49098E-05
GOTERM_CC_DIRECT	GO:0016328~lateral plasma membrane	7	2.845528455	7.01936E-05
GOTERM_CC_DIRECT	GO:0042470~melanosome	8	3.25203252	0.000398036
GOTERM_CC_DIRECT	GO:0016323~basolateral plasma membrane	10	4.06504065	0.000690812
GOTERM_CC_DIRECT	GO:0005737~cytoplasm	93	37.80487805	0.000979857
GOTERM_CC_DIRECT	GO:0005887~integral component of plasma membrane	34	13.82113821	0.001096818
GOTERM_CC_DIRECT	GO:0001726~ruffle	7	2.845528455	0.001266403
GOTERM_CC_DIRECT	GO:0030027~lamellipodium	9	3.658536585	0.00136351
GOTERM_CC_DIRECT	GO:0045121~membrane raft	10	4.06504065	0.001775488
GOTERM_CC_DIRECT	GO:0030175~filopodium	6	2.43902439	0.002529975
GOTERM_CC_DIRECT	GO:0005923~bicellular tight junction	7	2.845528455	0.003995982
GOTERM_CC_DIRECT	GO:0005882~intermediate filament	7	2.845528455	0.003995982
GOTERM_CC_DIRECT	GO:0031225~anchored component of membrane	7	2.845528455	0.003995982
GOTERM_CC_DIRECT	GO:0005604~basement membrane	6	2.43902439	0.004020885
GOTERM_CC_DIRECT	GO:0030426~growth cone	7	2.845528455	0.004541893
GOTERM_CC_DIRECT	GO:0032587~ruffle membrane	6	2.43902439	0.004714772
GOTERM_CC_DIRECT	GO:0008305~integrin complex	4	1.62601626	0.005345833
GOTERM_CC_DIRECT	GO:0042383~sarcolemma	6	2.43902439	0.005489782
GOTERM_CC_DIRECT	GO:0031093~platelet alpha granule lumen	5	2.032520325	0.006089339
GOTERM_CC_DIRECT	GO:0005581~collagen trimer	6	2.43902439	0.007640394
GOTERM_CC_DIRECT	GO:0009897~external side of plasma membrane	9	3.658536585	0.007728172
GOTERM_CC_DIRECT	GO:0015629~actin cytoskeleton	9	3.658536585	0.008834129

GOTERM_CC_DIRECT	GO:0031091~platelet alpha granule	3	1.219512195	0.014385346
GOTERM_CC_DIRECT	GO:0072686~mitotic spindle	4	1.62601626	0.017010652
GOTERM_CC_DIRECT	GO:0045177~apical part of cell	5	2.032520325	0.017685321
GOTERM_CC_DIRECT	GO:0019898~extrinsic component of membrane	5	2.032520325	0.023701701
GOTERM_CC_DIRECT	GO:0032154~cleavage furrow	4	1.62601626	0.024423122
GOTERM_CC_DIRECT	GO:0031258~lamellipodium membrane	3	1.219512195	0.025883019
GOTERM_CC_DIRECT	GO:0051233~spindle midzone	3	1.219512195	0.025883019
GOTERM_CC_DIRECT	GO:0043034~costamere	3	1.219512195	0.025883019
GOTERM_CC_DIRECT	GO:0034667~integrin alpha3-beta1 complex	2	0.81300813	0.026382767
GOTERM_CC_DIRECT	GO:0043202~lysosomal lumen	5	2.032520325	0.026616046
GOTERM_CC_DIRECT	GO:0034707~chloride channel complex	4	1.62601626	0.027222748
GOTERM_CC_DIRECT	GO:0043235~receptor complex	6	2.43902439	0.027305311
GOTERM_CC_DIRECT	GO:0005911~cell-cell junction	7	2.845528455	0.02748025
GOTERM_CC_DIRECT	GO:0030496~midbody	6	2.43902439	0.028948171
GOTERM_CC_DIRECT	GO:0015630~microtubule cytoskeleton	6	2.43902439	0.03613784
GOTERM_CC_DIRECT	GO:0031594~neuromuscular junction	4	1.62601626	0.036600944
GOTERM_CC_DIRECT	GO:0034666~integrin alpha2-beta1 complex	2	0.81300813	0.039313036
GOTERM_CC_DIRECT	GO:0097149~centralspindlin complex	2	0.81300813	0.039313036
GOTERM_CC_DIRECT	GO:0044393~microspike	2	0.81300813	0.039313036
GOTERM_CC_DIRECT	GO:0019897~extrinsic component of plasma membrane	3	1.219512195	0.043116912
GOTERM_CC_DIRECT	GO:0005623~cell	5	2.032520325	0.044303661
GOTERM_CC_DIRECT	GO:0005903~brush border	4	1.62601626	0.045519089
GOTERM_MF_DIRECT	GO:0005178~integrin binding	19	7.723577236	5.89619E-15
GOTERM_MF_DIRECT	GO:0005515~protein binding	175	71.13821138	5.26675E-12
GOTERM_MF_DIRECT	GO:0005509~calcium ion binding	34	13.82113821	1.6431E-09
GOTERM_MF_DIRECT	GO:0002020~protease binding	13	5.284552846	1.67672E-08
GOTERM_MF_DIRECT	GO:0098641~cadherin binding involved in cell-cell adhesion	20	8.130081301	2.70349E-08
GOTERM_MF_DIRECT	GO:0043236~laminin binding	8	3.25203252	3.70454E-08
GOTERM_MF_DIRECT	GO:0004222~metalloendopeptidase activity	12	4.87804878	5.24682E-07
GOTERM_MF_DIRECT	GO:0001968~fibronectin binding	7	2.845528455	1.27624E-06
GOTERM_MF_DIRECT	GO:0005518~collagen binding	9	3.658536585	1.77251E-06
GOTERM_MF_DIRECT	GO:0004252~serine-type endopeptidase activity	15	6.097560976	1.48661E-05
GOTERM_MF_DIRECT	GO:0008201~heparin binding	12	4.87804878	1.57453E-05
GOTERM_MF_DIRECT	GO:0001618~virus receptor activity	8	3.25203252	5.41413E-05
GOTERM_MF_DIRECT	GO:0008083~growth factor activity	11	4.471544715	9.53695E-05
GOTERM_MF_DIRECT	GO:0048306~calcium-dependent protein binding	7	2.845528455	0.000154472
GOTERM_MF_DIRECT	GO:0050840~extracellular matrix binding	5	2.032520325	0.00043709
GOTERM_MF_DIRECT	GO:0003779~actin binding	13	5.284552846	0.000562345
GOTERM_MF_DIRECT	GO:0044548~S100 protein binding	4	1.62601626	0.00069563
GOTERM_MF_DIRECT	GO:0005125~cytokine activity	10	4.06504065	0.000842758
GOTERM_MF_DIRECT	GO:0017147~Wnt-protein binding	5	2.032520325	0.00087095
GOTERM_MF_DIRECT	GO:0019834~phospholipase A2 inhibitor activity	3	1.219512195	0.001146305
GOTERM_MF_DIRECT	GO:0004869~cysteine-type endopeptidase inhibitor activity	5	2.032520325	0.001242298
GOTERM_MF_DIRECT	GO:0005201~extracellular matrix structural constituent	6	2.43902439	0.002443879

GOTERM_MF_DIRECT	GO:0019901~protein kinase binding	14	5.691056911	0.002525723
GOTERM_MF_DIRECT	GO:0005198~structural molecule activity	11	4.471544715	0.002558838
GOTERM_MF_DIRECT	GO:0042813~Wnt-activated receptor activity	4	1.62601626	0.003413753
GOTERM_MF_DIRECT	GO:0034235~GPI anchor binding	3	1.219512195	0.003902218
GOTERM_MF_DIRECT	GO:0008009~chemokine activity	5	2.032520325	0.004821112
GOTERM_MF_DIRECT	GO:0031995~insulin-like growth factor II binding	3	1.219512195	0.005155141
GOTERM_MF_DIRECT	GO:0019904~protein domain specific binding	9	3.658536585	0.009032837
GOTERM_MF_DIRECT	GO:0048407~platelet-derived growth factor binding	3	1.219512195	0.009850168
GOTERM_MF_DIRECT	GO:0004859~phospholipase inhibitor activity	3	1.219512195	0.009850168
GOTERM_MF_DIRECT	GO:0043394~proteoglycan binding	3	1.219512195	0.009850168
GOTERM_MF_DIRECT	GO:0050839~cell adhesion molecule binding	5	2.032520325	0.011029924
GOTERM_MF_DIRECT	GO:0005102~receptor binding	12	4.87804878	0.011106598
GOTERM_MF_DIRECT	GO:0031994~insulin-like growth factor I binding	3	1.219512195	0.011712069
GOTERM_MF_DIRECT	GO:0008191~metalloendopeptidase inhibitor activity	3	1.219512195	0.020528265
GOTERM_MF_DIRECT	GO:0005520~insulin-like growth factor binding	3	1.219512195	0.025699877
GOTERM_MF_DIRECT	GO:0017124~SH3 domain binding	6	2.43902439	0.02588126
GOTERM_MF_DIRECT	GO:0032403~protein complex binding	8	3.25203252	0.026009562
GOTERM_MF_DIRECT	GO:0008237~metallopeptidase activity	5	2.032520325	0.026834811
GOTERM_MF_DIRECT	GO:0042803~protein homodimerization activity	18	7.317073171	0.027876714
GOTERM_MF_DIRECT	GO:0042802~identical protein binding	18	7.317073171	0.034191453
GOTERM_MF_DIRECT	GO:0005254~chloride channel activity	4	1.62601626	0.039720521
GOTERM_MF_DIRECT	GO:0045296~cadherin binding	3	1.219512195	0.040608211
GOTERM_MF_DIRECT	GO:0005540~hyaluronic acid binding	3	1.219512195	0.040608211
GOTERM_MF_DIRECT	GO:0005544~calcium-dependent phospholipid binding	4	1.62601626	0.047469748
KEGG_PATHWAY	hsa04512:ECM-receptor interaction	14	5.691056911	5.85E-10
KEGG_PATHWAY	hsa05200:Pathways in cancer	24	9.756097561	2.26E-08
KEGG_PATHWAY	hsa05205:Proteoglycans in cancer	17	6.910569106	6.04E-08
KEGG_PATHWAY	hsa04510:Focal adhesion	16	6.504065041	0.000000556
KEGG_PATHWAY	hsa04151:PI3K-Akt signaling pathway	18	7.317073171	0.0000201
KEGG_PATHWAY	hsa04670:Leukocyte transendothelial migration	10	4.06504065	0.0000678
KEGG_PATHWAY	hsa05222:Small cell lung cancer	8	3.25203252	0.00033
KEGG_PATHWAY	hsa04060:Cytokine-cytokine receptor interaction	12	4.87804878	0.001319575
KEGG_PATHWAY	hsa05219:Bladder cancer	5	2.032520325	0.003600229
KEGG_PATHWAY	hsa04066:HIF-1 signaling pathway	7	2.845528455	0.003676269
KEGG_PATHWAY	hsa05412:Arrhythmogenic right ventricular cardiomyopathy (ARVC)	6	2.43902439	0.003725881
KEGG_PATHWAY	hsa04810:Regulation of actin cytoskeleton	10	4.06504065	0.005227546
KEGG_PATHWAY	hsa04015:Rap1 signaling pathway	10	4.06504065	0.005227546
KEGG_PATHWAY	hsa05146:Amoebiasis	7	2.845528455	0.00597232
KEGG_PATHWAY	hsa05144:Malaria	5	2.032520325	0.006850932
KEGG_PATHWAY	hsa04611:Platelet activation	7	2.845528455	0.015499847
KEGG_PATHWAY	hsa05230:Central carbon metabolism in cancer	5	2.032520325	0.017214857
KEGG_PATHWAY	hsa04115:p53 signaling pathway	5	2.032520325	0.020048517
KEGG_PATHWAY	hsa00010:Glycolysis / Gluconeogenesis	5	2.032520325	0.020048517
KEGG_PATHWAY	hsa04550:Signaling pathways regulating pluripotency of stem cells	7	2.845528455	0.021550131

KEGG_PATHWAY	hsa04610:Complement and coagulation cascades	5	2.032520325	0.022088473
KEGG_PATHWAY	hsa04514:Cell adhesion molecules (CAMs)	7	2.845528455	0.022928144
KEGG_PATHWAY	hsa04390:Hippo signaling pathway	7	2.845528455	0.029864674
KEGG_PATHWAY	hsa05202:Transcriptional misregulation in cancer	7	2.845528455	0.045345963
KEGG_PATHWAY	hsa04640:Hematopoietic cell lineage	5	2.032520325	0.046105277
KEGG_PATHWAY	hsa04064:NF-kappa B signaling pathway	5	2.032520325	0.046105277
KEGG_PATHWAY	hsa04360:Axon guidance	6	2.43902439	0.04742403
KEGG_PATHWAY	hsa05323:Rheumatoid arthritis	5	2.032520325	0.047743658

Table S5. Reasons for exclusion from the evaluation of prognostic factors and establishment of nomogram

Case ID	Size	T	N	AJCC stage	Residual tumor	Surgical treatment	History of radiation therapy	History of targeted molecular therapy	Tobacco smoking history	Alocohol drinking history	History of chronic pancreatitis	History of diabetes
TCGA-IB-A7LX			NA									
TCGA-HZ-8637							NA		NA		NA	NA
TCGA-HZ-A77P							NA					
TCGA-XD-AAUI											NA	
TCGA-FB-A4P5							NA					
TCGA-IB-AAUT	NA											
TCGA-IB-A5SS						NA						
TCGA-PZ-A5RE					NA					NA	NA	NA
TCGA-HZ-A4BK							NA		NA		NA	NA
TCGA-2J-AAB6									NA			
TCGA-OE-A75W							NA					
TCGA-HZ-A49I							NA		NA		NA	NA
TCGA-HZ-8519							NA		NA		NA	NA
TCGA-FB-A78T							NA				NA	
TCGA-HZ-8002							NA		NA	NA	NA	NA
TCGA-3E-AAAZ					NA		NA			NA	NA	NA
TCGA-HV-A5A4	NA				NA							
TCGA-FB-A4P6					NA		NA					
TCGA-IB-A5ST							NA					
TCGA-HZ-7919							NA	NA	NA		NA	NA
TCGA-HZ-8003							NA		NA	NA	NA	NA
TCGA-HV-AA8V	NA											
TCGA-HZ-8001				NA			NA		NA	NA	NA	NA
TCGA-HZ-8317							NA		NA		NA	NA
TCGA-2L-AAQL					NA							
TCGA-Q3-A5QY								NA				
TCGA-HZ-A49G							NA		NA		NA	NA
TCGA-HV-A5A5	NA				NA							
TCGA-IB-A7M4	NA											
TCGA-2J-AABT							NA					
TCGA-HZ-7926					NA		NA	NA	NA		NA	NA
TCGA-2L-AAQI												NA
TCGA-FB-A545	NA				NA		NA				NA	
TCGA-2J-AABK									NA			
TCGA-2J-AAB8							NA		NA			
TCGA-HZ-A49H							NA		NA		NA	NA
TCGA-HZ-8315							NA		NA	NA	NA	NA
TCGA-HV-AA8X	NA											
TCGA-HZ-A77O							NA					
TCGA-HV-A7OL	NA											
TCGA-IB-7888									NA			
TCGA-YH-A8SY				NA	NA							
TCGA-IB-7652								NA				
TCGA-HZ-7925							NA		NA		NA	NA
TCGA-HV-A5A6	NA					NA				NA		
TCGA-Z5-AAPL	NA					NA				NA	NA	NA
TCGA-US-A779							NA	NA				
TCGA-HZ-A4BH							NA	NA	NA		NA	NA
TCGA-FB-AAPS								NA				
TCGA-3A-A9I7	NA							NA	NA			
TCGA-IB-8126							NA					
TCGA-3A-A9I5		NA		NA					NA			
TCGA-3E-AAAY					NA							
TCGA-FB-AAPY											NA	
TCGA-IB-8127										NA		
TCGA-IB-7645										NA		
TCGA-FB-A5VM							NA					
TCGA-LB-A8F3							NA				NA	
TCGA-HV-A5A3	NA		NA		NA				NA			
TCGA-Q3-AA2A	NA				NA						NA	
TCGA-H6-A45N					NA		NA					
TCGA-HZ-A77Q							NA	NA				
TCGA-HZ-8005									NA	NA	NA	NA
TCGA-2J-AABR									NA			

Table S6. Gene set enrichment analyses between high and low risk group in TCGA PAAD dataset

Terms enriched in high risk group

NAME	SIZE	ES	NES	NOM p-val	FDR q-val
**C2 KEGG (19 terms)**					
KEGG_NUCLEOTIDE_EXCISION_REPAIR	44	0.7201397	2.0976436	0	0.014243265
KEGG_DNA_REPLICATION	36	0.76967376	1.9800676	0.001984127	0.032056652
KEGG_CELL_CYCLE	124	0.6190967	1.9787879	0.002061856	0.02192012
KEGG_PENTOSE_PHOSPHATE_PATHWAY	26	0.6525248	1.9349254	0.004081633	0.031387735
KEGG_HOMOLOGOUS_RECOMBINATION	28	0.6866101	1.8651135	0	0.05900724
KEGG_P53_SIGNALING_PATHWAY	67	0.53078645	1.8600821	0	0.051003285
KEGG_MISMATCH_REPAIR	23	0.71814543	1.8482338	0.005988024	0.04848411
KEGG_PROTEASOME	44	0.72454447	1.8142364	0.001988072	0.057262708
KEGG_PYRIMIDINE_METABOLISM	96	0.52235556	1.8008125	0.005952381	0.059701934
KEGG_BASE_EXCISION_REPAIR	33	0.64210325	1.744873	0.002024292	0.09585684
KEGG_SPLICEOSOME	124	0.5726161	1.7331576	0.002004008	0.09630436
KEGG_ONE_CARBON_POOL_BY_FOLATE	17	0.6265623	1.7208614	0.004166667	0.09855121
KEGG_N_GLYCAN_BIOSYNTHESIS	46	0.5590183	1.7089907	0.012072435	0.10039359
KEGG_HUNTINGTONS_DISEASE	172	0.4956008	1.6758672	0.038934425	0.12364742
KEGG_AMINOACYL_TRNA_BIOSYNTHESIS	41	0.612716	1.6366665	0.016771488	0.15375604
KEGG_RNA_POLYMERASE	29	0.5731571	1.6191819	0.030425964	0.1654411
KEGG_RNA_DEGRADATION	57	0.54382604	1.6059408	0.024096385	0.17073308
KEGG_OOCYTE_MEIOSIS	111	0.45920682	1.5974178	0.025586354	0.17047107
KEGG_ALZHEIMERS_DISEASE	156	0.43739977	1.5367693	0.09306931	0.24166852
**C5**					
**Biological process (646 terms)**					
GO_CELL_CYCLE_G1_S_PHASE_TRANSITION	108	0.6594155	2.079041	0	0.17302747
GO_MITOTIC_CYTOKINESIS	31	0.77121973	2.0544686	0	0.12073248
GO_METAPHASE_PLATE_CONGRESSION	40	0.7632659	2.0319428	0	0.112749554
GO_CHROMOSOME_SEGREGATION	259	0.6232573	2.0318973	0	0.08456216
GO_MITOTIC_SISTER_CHROMATID_SEGREGATION	87	0.70989907	2.0139432	0	0.09089815
GO_DNA_PACKAGING	186	0.5678415	2.0118885	0	0.07762929
GO_SISTER_CHROMATID_SEGREGATION	169	0.67860454	2.009597	0	0.068468295
GO_NUCLEAR_CHROMOSOME_SEGREGATION	216	0.6231709	2.0071008	0	0.06222519
GO_MITOTIC_NUCLEAR_DIVISION	349	0.61095	2.006609	0	0.05602736
GO_MITOTIC_CELL_CYCLE	738	0.59008014	2.003901	0	0.053431753
GO_NEGATIVE_REGULATION_OF_CELL_DIVISION	58	0.6338843	2.0016801	0.002066116	0.050330997
GO_CELL_DIVISION	449	0.57844305	2.0005815	0	0.046962485
GO_POSITIVE_REGULATION_OF_CYTOKINESIS	35	0.6032073	1.9870645	0	0.053053077
GO_CHROMOSOME_LOCALIZATION	59	0.6648509	1.9844478	0	0.05093084
GO_ORGANELLE_FISSION	471	0.561181	1.9843669	0	0.04763153
GO_REGULATION_OF_CYTOKINESIS	63	0.5958506	1.9737135	0	0.05116467
GO_CHROMOSOME_CONDENSATION	29	0.7009857	1.9726745	0	0.048777815

GO_DNA_CONFORMATION_CHANGE	263	0.56042385	1.9689437	0.00204499	0.048354197
GO_TELOMERE_ORGANIZATION	103	0.60528266	1.9628264	0.002096436	0.049389604
GO_DNA_REPLICATION_INDEPENDENT_NUCLEOSOME_ORGANIZATION	51	0.66559476	1.9589338	0	0.04963587
GO_ATP_DEPENDENT_CHROMATIN_REMODELING	72	0.6025981	1.9567213	0	0.049041003
GO_SISTER_CHROMATID_COHESION	107	0.68042785	1.9516385	0	0.05041581
GO_HISTONE_EXCHANGE	51	0.6394713	1.9339594	0	0.061044134
GO_CELL_CYCLE_CHECKPOINT	185	0.5870263	1.9336329	0	0.05865187
GO_ANAPHASE_PROMOTING_COMPLEX_DEPENDENT_CATABOLIC_PROCESS	76	0.7079842	1.9265618	0.002012072	0.06160793
GO_CELL_CYCLE_PHASE_TRANSITION	249	0.5743212	1.924497	0	0.060587894
GO_REGULATION_OF_NUCLEAR_DIVISION	159	0.53920203	1.9222364	0	0.060322974
GO_POSITIVE_REGULATION_OF_LIGASE_ACTIVITY	108	0.6125599	1.9175805	0.001956947	0.062291212
GO_DNA_BIOSYNTHETIC_PROCESS	112	0.60502017	1.9162903	0	0.06116454
GO_NUCLEOBASE_CONTAINING_SMALL_MOLECULE_INTERCONVERSION	21	0.71639425	1.9149318	0.002118644	0.06018737
GO_DNA_RECOMBINATION	199	0.57265526	1.9129227	0	0.059630796
GO_DNA_REPAIR	454	0.5581603	1.9126428	0	0.05802184
GO_PROTEIN_DNA_COMPLEX_SUBUNIT_ORGANIZATION	223	0.53689474	1.9116246	0	0.056921497
GO_REGULATION_OF_INTRINSIC_APOPTOTIC_SIGNALING_PATHWAY_IN_RESPONSE_TO_DNA_DAMAGE_BY_P53_CLASS_MEDIATOR	14	0.7134073	1.9109195	0	0.05590264
GO_CHROMATIN_ASSEMBLY_OR_DISASSEMBLY	170	0.5439884	1.9093914	0	0.055217147
GO_MITOTIC_CELL_CYCLE_CHECKPOINT	133	0.58693933	1.9079821	0.002028398	0.05436471
GO_RECOMBINATIONAL_REPAIR	72	0.6230198	1.9062333	0	0.054252245
GO_REGULATION_OF_CELL_DIVISION	265	0.49105945	1.9006313	0	0.056953292
GO_CENTROMERE_COMPLEX_ASSEMBLY	46	0.6779127	1.8996428	0.002141328	0.056270037
GO_MACROMOLECULAR_COMPLEX_DISASSEMBLY	180	0.56804246	1.8982546	0	0.055735156
GO_DNA_STRAND_ELONGATION	30	0.754878	1.8978914	0.004081633	0.054624617
GO_TRANSCRIPTION_COUPLED_NUCLEOTIDE_EXCISION_REPAIR	72	0.6381792	1.8932533	0	0.056440137
GO_DNA_STRAND_ELONGATION_INVOLVED_IN_DNA_REPLICATION	25	0.772086	1.8922454	0.006048387	0.055737335
GO_NEGATIVE_REGULATION_OF_MITOTIC_NUCLEAR_DIVISION	32	0.67065674	1.8917006	0.006122449	0.054746598
GO_NEGATIVE_REGULATION_OF_NUCLEAR_DIVISION	44	0.6242278	1.8906703	0.002070393	0.054341964
GO_CELL_CYCLE_PROCESS	1031	0.5272319	1.8901148	0	0.05352587
GO_CYTOSKELETON_DEPENDENT_CYTOKINESIS	39	0.67397314	1.8823988	0	0.057473022
GO_DOUBLE_STRAND_BREAK_REPAIR	158	0.5644916	1.8800385	0	0.057996474
GO_MITOTIC_RECOMBINATION	41	0.7160155	1.8800086	0.004106776	0.056845613
GO_REGULATION_OF_MICROTUBULE_POLYMERIZATION_OR_DEPOLYMERIZATION	170	0.55327034	1.8796449	0	0.0559638
GO_CELL_DIFFERENTIATION_INVOLVED_IN_EMBRYONIC_PLACENTA_DEVELOPMENT	25	0.6224229	1.8785065	0	0.055537615
GO_DEOXYRIBONUCLEOTIDE_BIOSYNTHETIC_PROCESS	12	0.7682248	1.8755125	0	0.056754433
GO_REGULATION_OF_ATTACHMENT_OF_SPINDLE_MICROTUBULES_TO_KINETOCHORE	10	0.8647281	1.874774	0	0.056090236
GO_POSTREPLICATION_REPAIR	51	0.6270003	1.8745784	0	0.055233143
GO_REGULATION_OF_EXIT_FROM_MITOSIS	15	0.7092638	1.8734727	0.001984127	0.055012096
GO_CELLULAR_PROTEIN_COMPLEX_DISASSEMBLY	121	0.60866874	1.8680725	0.006024096	0.05695752
GO_NEGATIVE_REGULATION_OF_PROTEIN_MODIFICATION_BY_SMALL_PROTEIN_CONJUGATION_OR_REMOVAL	135	0.5763723	1.8665744	0.003984064	0.057070322
GO_RETROGRADE_VESICLE_MEDIATED_TRANSPORT_GOLGI_TO_ER	76	0.59436154	1.8658687	0.001926782	0.05659839
GO_MITOTIC_SPINDLE_ORGANIZATION	67	0.6308847	1.8642325	0	0.056519132

GO_NUCLEOTIDE_EXCISION_REPAIR	111	0.6047418	1.8637582	0	0.055921607
GO_REGULATION_OF_TRANSCRIPTION_INVOLVED_IN_G1_S_TRANSITION_OF_MITOTIC_CELL_CYCLE	25	0.71017885	1.8603126	0	0.05764584
GO_CELL_CYCLE	1260	0.5062813	1.8590708	0.002024292	0.057687998
GO_DNA_REPLICATION_INITIATION	29	0.7229915	1.8582195	0.012195122	0.057261914
GO_REGULATION_OF_CHROMOSOME_SEGREGATION	82	0.6246313	1.8563925	0.001988072	0.057641525
GO_DNA_SYNTHESIS_INVOLVED_IN_DNA_REPAIR	72	0.600466	1.8559506	0.00610998	0.056985766
GO_GUANOSINE_CONTAINING_COMPOUND_BIOSYNTHETIC_PROCESS	14	0.72929907	1.8556812	0.006048387	0.056431606
GO_DNA_DAMAGE_RESPONSE_DETECTION_OF_DNA_DAMAGE	36	0.67355824	1.853649	0	0.057087526
GO_DNA_REPLICATION	199	0.58835423	1.8531232	0.004032258	0.056539003
GO_DNA_GEOMETRIC_CHANGE	79	0.64581376	1.8512492	0	0.056980796
GO_REGULATION_OF_MITOTIC_CELL_CYCLE	453	0.5071375	1.850487	0	0.05660831
GO_REGULATION_OF_CENTROSOME_CYCLE	38	0.6647558	1.8489791	0.002028398	0.056536384
GO_REGULATION_OF_LIGASE_ACTIVITY	128	0.57687914	1.8432896	0.002008032	0.059795626
GO_REGULATION_OF_TRANSLATION_IN_RESPONSE_TO_STRESS	19	0.7035845	1.8424728	0	0.059348173
GO_NUCLEOTIDE_PHOSPHORYLATION	57	0.5553161	1.842338	0.00631579	0.058638163
GO_DNA_METABOLIC_PROCESS	717	0.5147718	1.8394678	0	0.059833974
GO_DNA_INTEGRITY_CHECKPOINT	140	0.56185144	1.8364042	0.002020202	0.06106539
GO_REGULATION_OF_PROTEIN_UBIQUITINATION_INVOLVED_IN_UBIQUITIN_DEPENDENT_PROTEIN_CATABOLIC_PROCESS	102	0.59517676	1.8353814	0	0.060981054
GO_CYTOKINESIS	84	0.57650286	1.8325056	0.004166667	0.06209914
GO_ADP_METABOLIC_PROCESS	45	0.5654191	1.8309293	0	0.062392663
GO_NUCLEOSIDE_MONOPHOSPHATE_BIOSYNTHETIC_PROCESS	79	0.5514237	1.830807	0.006134969	0.06177915
GO_REGULATION_OF_CELL_CYCLE_PROCESS	542	0.4906111	1.8300204	0	0.061481383
GO_MITOTIC_DNA_INTEGRITY_CHECKPOINT	96	0.57074857	1.8278122	0.00204918	0.062323567
GO_CELL_SEPARATION_AFTER_CYTOKINESIS	17	0.7482304	1.8264953	0	0.06255374
GO_REGULATION_OF_CELL_CYCLE_PHASE_TRANSITION	314	0.5196457	1.823072	0	0.064318925
GO_DNA_DEPENDENT_DNA_REPLICATION	93	0.6308172	1.8222849	0.006185567	0.064130776
GO_REGULATION_OF_SUBSTRATE_ADHESION_DEPENDENT_CELL_SPREADING	42	0.5442426	1.8202811	0	0.06478797
GO_POSITIVE_REGULATION_OF_MITOTIC_NUCLEAR_DIVISION	50	0.53670204	1.8185797	0.002079002	0.065406114
GO_MITOTIC_SPINDLE_ASSEMBLY	40	0.6259853	1.8131715	0.002040816	0.06872748
GO_CELL_CYCLE_DNA_REPLICATION	11	0.8093078	1.8111064	0.00204918	0.06932733
GO_ATTACHMENT_OF_SPINDLE_MICROTUBULES_TO_KINETOCHORE	14	0.7738783	1.8090951	0	0.07022309
GO_TELOMERE_MAINTENANCE_VIA_RECOMBINATION	32	0.71336704	1.8080405	0.012552301	0.07017772
GO_RNA_CAPPING	35	0.6500747	1.8052832	0.004048583	0.0715783
GO_ATP_GENERATION_FROM_ADP	37	0.5888407	1.8035351	0.004385965	0.07209955
GO_G1_DNA_DAMAGE_CHECKPOINT	70	0.56345177	1.8030847	0.001992032	0.071644515
GO_REGULATION_OF_SISTER_CHROMATID_SEGREGATION	65	0.6092445	1.802754	0.005952381	0.0711142
GO_REGULATION_OF_PROTEIN_COMPLEX_DISASSEMBLY	208	0.50912297	1.801465	0	0.07115715
GO_REGULATION_OF_TRANSLATIONAL_INITIATION_IN_RESPONSE_TO_STRESS	13	0.7393982	1.8002415	0	0.07148471
GO_REGULATION_OF_DNA_REPAIR	72	0.5643014	1.7994635	0.00203252	0.07127585
GO_MULTI_ORGANISM_LOCALIZATION	66	0.60049486	1.7993892	0.001992032	0.07061423
GO_PROTEIN_LOCALIZATION_TO_CHROMOSOME_CENTROMERIC_REGION	12	0.83075815	1.7988199	0	0.07028531
GO_NEGATIVE_REGULATION_OF_MITOTIC_CELL_CYCLE	188	0.52133536	1.7979151	0.00409836	0.070325114
GO_KINETOCHORE_ORGANIZATION	11	0.80802447	1.7962064	0.002066116	0.07082972

GO_MEIOTIC_CELL_CYCLE	176	0.4838582	1.7961382	0.002222222	0.0701811
GO_NEGATIVE_REGULATION_OF_INTRINSIC_APOPTOTIC_SIGNALING_PATHWAY_IN_RESPONSE_TO_DNA_DAMAGE	25	0.56131184	1.7943506	0.00203666	0.07084026
GO_NEGATIVE_REGULATION_OF_CELL_CYCLE_PROCESS	206	0.50823057	1.7938737	0.004032258	0.070462845
GO_TRANSLATIONAL_ELONGATION	110	0.629715	1.7927814	0.016460905	0.070737235
GO_REGULATION_OF_SPINDLE_ORGANIZATION	19	0.7267397	1.7925236	0.002028398	0.07014979
GO_NEGATIVE_REGULATION_OF_CELL_CYCLE_PHASE_TRANSITION	141	0.52799565	1.7925135	0.00407332	0.069550104
GO_POSITIVE_REGULATION_OF_VIRAL_PROCESS	86	0.548578	1.7916471	0.004024145	0.069623984
GO_TRANSLATIONAL_TERMINATION	92	0.66141635	1.7912972	0.012295082	0.069281995
GO_REGULATION_OF_MICROTUBULE_BASED_PROCESS	232	0.5188817	1.7911026	0	0.068790466
GO_DEOXYRIBONUCLEOTIDE_METABOLIC_PROCESS	33	0.6126696	1.7908406	0.004246285	0.0683846
GO_PURINE_DEOXYRIBONUCLEOTIDE_METABOLIC_PROCESS	14	0.69748753	1.7874415	0	0.070244305
GO_MICROTUBULE_DEPOLYMERIZATION	12	0.7090188	1.7863799	0.01417004	0.07037838
GO_INTERSTRAND_CROSS_LINK_REPAIR	43	0.6013596	1.7846135	0.002057613	0.070859075
GO_CELLULAR_RESPONSE_TO_DNA_DAMAGE_STIMULUS	683	0.50166297	1.7844547	0.004032258	0.07030248
GO_NEGATIVE_REGULATION_OF_PROTEIN_COMPLEX_DISASSEMBLY	163	0.5090511	1.7836775	0.002028398	0.070243925
GO_VIRION_ASSEMBLY	36	0.6286639	1.7814549	0.002	0.07128069
GO_REGULATION_OF_CELLULAR_AMINO_ACID_METABOLIC_PROCESS	66	0.5912529	1.7800603	0.008368201	0.07176949
GO_MULTI_ORGANISM_ORGANELLE_ORGANIZATION	23	0.68883085	1.7789183	0.001996008	0.07195913
GO_TROPHOBLAST_GIANT_CELL_DIFFERENTIATION	12	0.69022644	1.7769783	0.010799136	0.07286079
GO_POSITIVE_REGULATION_OF_VIRAL_RELEASE_FROM_HOST_CELL	15	0.7426813	1.7744824	0.005758158	0.07419394
GO_NEGATIVE_REGULATION_OF_CHROMOSOME_SEGREGATION	27	0.66377854	1.7719053	0.008032128	0.07558655
GO_NUCLEAR_ENVELOPE_REASSEMBLY	17	0.7206396	1.7717979	0	0.075102516
GO_NEGATIVE_REGULATION_OF_PROTEIN_TYROSINE_KINASE_ACTIVITY	20	0.64486015	1.7716019	0.001980198	0.07463592
GO_DEOXYRIBONUCLEOSIDE_TRIPHOSPHATE_METABOLIC_PROCESS	16	0.69725454	1.768188	0.008333334	0.076560736
GO_FOLIC_ACID_CONTAINING_COMPOUND_BIOSYNTHETIC_PROCESS	12	0.71220076	1.7679068	0.00409836	0.076205686
GO_REGULATION_OF_EXOSOMAL_SECRETION	17	0.6799501	1.767176	0.009940358	0.076118976
GO_PROTEIN_LOCALIZATION_TO_CHROMOSOME	42	0.6209102	1.7666308	0.012269938	0.075891905
GO_CELL_CYCLE_G2_M_PHASE_TRANSITION	135	0.539866	1.7662115	0.00204918	0.075547256
GO_MITOCHONDRIAL_TRANSLATION	105	0.6411833	1.7661473	0.018518519	0.0750145
GO_PROTEASOMAL_PROTEIN_CATABOLIC_PROCESS	267	0.5174959	1.7644219	0.004048583	0.07577184
GO_POSITIVE_REGULATION_OF_MITOTIC_SISTER_CHROMATID_SEPARATION	14	0.73466486	1.7625535	0.004024145	0.07654687
GO_NIK_NF_KAPPAB_SIGNALING	83	0.58414763	1.7624601	0.006134969	0.07605178
GO_DNA_TEMPLATED_TRANSCRIPTION_ELONGATION	92	0.57401276	1.7610134	0.002008032	0.07656615
GO_G2_DNA_DAMAGE_CHECKPOINT	33	0.6117437	1.7604189	0.002074689	0.07645226
GO_NUCLEOTIDE_EXCISION_REPAIR_DNA_INCISION	39	0.616001	1.7570999	0.002040816	0.078350075
GO_NON_RECOMBINATIONAL_REPAIR	70	0.5445053	1.7564317	0.008350731	0.07827828
GO_NUCLEOSIDE_SALVAGE	17	0.6594303	1.7537136	0.004140787	0.07985341
GO_SIGNAL_TRANSDUCTION_IN_RESPONSE_TO_DNA_DAMAGE	93	0.52890885	1.7519087	0.001996008	0.080803335
GO_MEIOTIC_CELL_CYCLE_PROCESS	143	0.48117754	1.7515141	0.008849558	0.08058891
GO_TRANSCRIPTION_ELONGATION_FROM_RNA_POLYMERASE_II_PROMOTER	77	0.57432973	1.7462572	0	0.08439547
GO_EXIT_FROM_MITOSIS	12	0.6857492	1.7448534	0.003960396	0.08524574
GO_TELOMERE_CAPPING	29	0.5822982	1.7446738	0.021008404	0.084795065
GO_APICAL_JUNCTION_ASSEMBLY	39	0.5617546	1.7444959	0.008298756	0.08436369

GO_SPINDLE_CHECKPOINT	24	0.6894951	1.7430066	0.014583333	0.08512426
GO_POSITIVE_REGULATION_OF_CELL_CYCLE_ARREST	82	0.5195382	1.7426795	0.00203252	0.08480592
GO_PEPTIDYL_PROLINE_MODIFICATION	53	0.50628847	1.742659	0.010869565	0.08426755
GO_REGULATION_OF_INTRINSIC_APOPTOTIC_SIGNALING_PATHWAY_BY_P53_CLASS_MEDIATOR	22	0.57435966	1.7398579	0.009784736	0.08606029
GO_MITOTIC_CHROMOSOME_CONDENSATION	14	0.75140095	1.7383711	0.007889546	0.086522676
GO_POSITIVE_REGULATION_OF_PROTEIN_MODIFICATION_BY_SMALL_PROTEIN_CONJUGATION_OR_REMOVAL	192	0.50574815	1.7349168	0	0.08886197
GO_POSITIVE_REGULATION_OF_CELL_CYCLE_PROCESS	240	0.45668328	1.7347728	0.004149378	0.08845597
GO_DNA_REPLICATION_CHECKPOINT	14	0.7143572	1.7333574	0.004166667	0.08910595
GO_POSITIVE_REGULATION_OF_SUBSTRATE_ADHESION_DEPENDENT_CELL_SPREADING	27	0.5420212	1.7315581	0.002262444	0.09013324
GO_SPLICEOSOMAL_SNRNP_ASSEMBLY	37	0.6423509	1.7309353	0.010121457	0.09010104
GO_NEGATIVE_REGULATION_OF_SIGNAL_TRANSDUCTION_BY_P53_CLASS_MEDIATOR	27	0.568996	1.7298098	0.011904762	0.09053733
GO_NUCLEAR_ENVELOPE_ORGANIZATION	77	0.58041215	1.7294037	0.004024145	0.09026247
GO_NUCLEUS_ORGANIZATION	131	0.5249544	1.7291876	0	0.08989718
GO_PROTEIN_LOCALIZATION_TO_KINETOCHORE	10	0.8234618	1.7276508	0.00203252	0.090741135
GO_PURINE_NUCLEOSIDE_MONOPHOSPHATE_BIOSYNTHETIC_PROCESS	58	0.54420274	1.7263952	0.016597511	0.09123835
GO_NUCLEOBASE_METABOLIC_PROCESS	38	0.5366523	1.7263659	0.008298756	0.09069273
GO_CHROMOSOME_ORGANIZATION	970	0.4727875	1.7259496	0.00814664	0.09049012
GO_ESTABLISHMENT_OF_PROTEIN_LOCALIZATION_TO_ORGANELLE	353	0.50328386	1.7258406	0.020491803	0.09003132
GO_POSITIVE_REGULATION_OF_TELOMERE_MAINTENANCE	46	0.59496236	1.72571	0.00408998	0.08962003
GO_REGULATION_OF_CENTROSOME_DUPLICATION	31	0.6211932	1.7253408	0.002028398	0.089393295
GO_GLYCOSYL_COMPOUND_BIOSYNTHETIC_PROCESS	113	0.48679727	1.7219335	0.004149378	0.09169525
GO_MONOSACCHARIDE_BIOSYNTHETIC_PROCESS	54	0.4940323	1.7170415	0.010845987	0.095835604
GO_PYRIMIDINE_NUCLEOSIDE_BIOSYNTHETIC_PROCESS	29	0.57662636	1.7162814	0.008368201	0.095952
GO_SIGNAL_TRANSDUCTION_BY_P53_CLASS_MEDIATOR	123	0.48191005	1.7117918	0.004032258	0.099519394
GO_RIBONUCLEOSIDE_DIPHOSPHATE_METABOLIC_PROCESS	62	0.49100196	1.7112136	0.00617284	0.099623956
GO_REGULATION_OF_DOUBLE_STRAND_BREAK_REPAIR	35	0.574451	1.710933	0.008247423	0.099326074
GO_MEIOSIS_I	81	0.48167685	1.7104652	0.004750594	0.0991738
GO_AMP_METABOLIC_PROCESS	13	0.6802312	1.7096415	0.003968254	0.09943079
GO_REGULATION_OF_TELOMERASE_RNA_LOCALIZATION_TO_CAJAL_BODY	15	0.7894643	1.7080733	0.014227643	0.100377515
GO_POSITIVE_REGULATION_OF_CELLULAR_PROTEIN_CATABOLIC_PROCESS	189	0.48823372	1.7070585	0.008016032	0.100768484
GO_TRANSLESION_SYNTHESIS	39	0.5749676	1.7067393	0.002061856	0.10053349
GO_NUCLEOTIDE_EXCISION_REPAIR_DNA_GAP_FILLING	24	0.64418495	1.7066292	0.010224949	0.10004112
GO_REGULATION_OF_RESPONSE_TO_DNA_DAMAGE_STIMULUS	140	0.49218425	1.7065214	0.002053388	0.099613644
GO_DNA_TEMPLATED_TRANSCRIPTION_TERMINATION	98	0.57550466	1.7051836	0.007920792	0.100293
GO_AMIDE_BIOSYNTHETIC_PROCESS	490	0.4984211	1.705073	0.033755273	0.09980847
GO_REGULATION_OF_INTRINSIC_APOPTOTIC_SIGNALING_PATHWAY_IN_RESPONSE_TO_DNA_DAMAGE	32	0.51905906	1.7044178	0.006036217	0.09975252
GO_REGULATION_OF_RNA_STABILITY	137	0.5245605	1.7044063	0.008097166	0.09921055
GO_MATURATION_OF_5_8S_RRNA	28	0.665308	1.7020051	0.016563147	0.10082497
GO_CELLULAR_COMPONENT_DISASSEMBLY	499	0.4264399	1.7010064	0	0.10127034
GO_NUCLEOSIDE_DIPHOSPHATE_METABOLIC_PROCESS	80	0.46829695	1.7008116	0.010309278	0.10089137
GO_BASE_EXCISION_REPAIR	39	0.5885106	1.7006632	0.014112903	0.10054234
GO_NEGATIVE_REGULATION_OF_RESPONSE_TO_DNA_DAMAGE_STIMULUS	49	0.52988714	1.7006015	0.008196721	0.10008834
GO_PROTEIN_TARGETING_TO_MITOCHONDRION	47	0.5936182	1.7004097	0.03869654	0.09972516

GO_SPINDLE_ASSEMBLY	69	0.5640803	1.6996964	0.010224949	0.099836
GO_RRNA_3_END_PROCESSING	13	0.76342064	1.6992583	0.014644352	0.099616945
GO_REGULATION_OF_ESTABLISHMENT_OF_PLANAR_POLARITY	110	0.501114	1.698461	0.016701462	0.09979157
GO_PYRUVATE_METABOLIC_PROCESS	62	0.48094097	1.6975366	0	0.10015196
GO_POSITIVE_REGULATION_OF_CHROMOSOME_SEGREGATION	25	0.6638723	1.6973723	0.012345679	0.099797376
GO_NCRNA_METABOLIC_PROCESS	509	0.532935	1.6961759	0.034907598	0.10051061
GO_REGULATION_OF_CELLULAR_AMINE_METABOLIC_PROCESS	88	0.4971241	1.6955172	0.016806724	0.100522935
GO_PENTOSE_PHOSPHATE_SHUNT	12	0.69673735	1.6938003	0.009881423	0.10167802
GO_NUCLEOTIDE_EXCISION_REPAIR_PREINCISION_COMPLEX_ASSEMBLY	29	0.6174684	1.6916432	0.003992016	0.10314338
GO_AMINO_ACID_ACTIVATION	51	0.61593795	1.6914419	0.016632017	0.10283297
GO_NUCLEOSIDE_MONOPHOSPHATE_METABOLIC_PROCESS	232	0.4728614	1.6911051	0.031185031	0.102691725
GO_REGULATION_OF_CELL_CYCLE_ARREST	103	0.4763836	1.6910665	0.004008016	0.10220632
GO_REGULATION_OF_DNA_TEMPLATED_TRANSCRIPTION_IN_RESPONSE_TO_STRESS	67	0.4943058	1.6900123	0.016427105	0.102614716
GO_POSITIVE_REGULATION_OF_GENE_EXPRESSION_EPIGENETIC	77	0.5043767	1.6896877	0.019480519	0.102489196
GO_MONOSACCHARIDE_CATABOLIC_PROCESS	57	0.49460027	1.6875899	0.009009009	0.1037311
GO_NUCLEOBASE_BIOSYNTHETIC_PROCESS	18	0.6439601	1.6863327	0.021097047	0.10433999
GO_MATURATION_OF_SSU_RRNA	41	0.656936	1.6852481	0.027253669	0.10492139
GO_MULTIVESICULAR_BODY_ORGANIZATION	30	0.6336862	1.6851829	0.014056225	0.10445602
GO_GLOBAL_GENOME_NUCLEOTIDE_EXCISION_REPAIR	32	0.6082239	1.68373	0.008016032	0.10541214
GO_BICELLULAR_TIGHT_JUNCTION_ASSEMBLY	31	0.5562197	1.6834726	0.022774328	0.10513558
GO_CELLULAR_MACROMOLECULAR_COMPLEX_ASSEMBLY	687	0.44487453	1.682835	0.002083333	0.10522068
GO_POSITIVE_REGULATION_OF_DNA_REPAIR	37	0.5645221	1.6824293	0.012244898	0.10507682
GO_REGULATION_OF_CELLULAR_PROTEIN_CATABOLIC_PROCESS	267	0.47290155	1.6824261	0.007984032	0.10458209
GO_PROTEASOME_ASSEMBLY	14	0.72373945	1.6820838	0.015686275	0.10443988
GO_BLASTOCYST_GROWTH	16	0.709049	1.6801829	0.012244898	0.10572605
GO_ANTIGEN_PROCESSING_AND_PRESENTATION_OF_PEPTIDE_ANTIGEN_VIA_MHC_CLASS_I	90	0.5393969	1.6791266	0.031380754	0.10627534
GO_POSITIVE_REGULATION_OF_DNA_METABOLIC_PROCESS	182	0.45509538	1.6778299	0.008179959	0.10707712
GO_NADH_METABOLIC_PROCESS	34	0.5654207	1.677182	0.02079002	0.107167825
GO_RIBOSOME_BIOGENESIS	300	0.5818069	1.675016	0.055900622	0.10884301
GO_RECIPROCAL_DNA_RECOMBINATION	39	0.53736556	1.6745574	0.019823788	0.10875994
GO_PURINE_NUCLEOBASE_BIOSYNTHETIC_PROCESS	12	0.6861267	1.674366	0.02258727	0.10841905
GO_RIBONUCLEOPROTEIN_COMPLEX_BIOGENESIS	424	0.5539283	1.6738896	0.048780486	0.1083543
GO_REGULATION_OF_SIGNAL_TRANSDUCTION_BY_P53_CLASS_MEDIATOR	160	0.49074864	1.6728058	0.008130081	0.10882209
GO_REGULATION_OF_SPINDLE_ASSEMBLY	14	0.7212587	1.6718248	0.003960396	0.109294064
GO_PROTEIN_LOCALIZATION_TO_ORGANELLE	538	0.46319988	1.6710272	0.00408998	0.10957328
GO_ETHER_METABOLIC_PROCESS	12	0.6292144	1.6698705	0.018099548	0.11035504
GO_MITOTIC_G2_M_TRANSITION_CHECKPOINT	18	0.6400335	1.6696733	0.026970955	0.1100414
GO_CHROMOSOME_ORGANIZATION_INVOLVED_IN_MEIOTIC_CELL_CYCLE	47	0.49190363	1.6691711	0.016470589	0.1100737
GO_RRNA_METABOLIC_PROCESS	250	0.5796282	1.668882	0.062626265	0.10985583
GO_PEPTIDE_METABOLIC_PROCESS	548	0.46339744	1.6677742	0.036796536	0.11044193
GO_MISMATCH_REPAIR	27	0.6059561	1.6654557	0.016666668	0.11227384
GO_PURINE_NUCLEOSIDE_BIOSYNTHETIC_PROCESS	83	0.47392043	1.6642703	0.020876827	0.1130194
GO_MATURATION_OF_5_8S_RRNA_FROM_TRICISTRONIC_RRNA_TRANSCRIPT_SSU_RRNA_5_8S_RRNA_LSU_RRNA_	19	0.6925778	1.6642603	0.016666668	0.11255172

GO_PROTEIN_HETEROTETRAMERIZATION	38	0.55923903	1.663671	0.03267974	0.11261031
GO_DNA_LIGATION	16	0.6472004	1.6635885	0.012448133	0.11219521
GO_GLUCOSE_CATABOLIC_PROCESS	28	0.5748583	1.6634889	0.025531914	0.11179181
GO_NUCLEAR_TRANSCRIBED_MRNA_CATABOLIC_PROCESS_EXONUCLEOLYTIC	30	0.6257131	1.663338	0.015936255	0.111445874
GO_REGULATION_OF_CELL_CYCLE	916	0.42964968	1.6619779	0.004140787	0.11218119
GO_STRAND_DISPLACEMENT	26	0.66460353	1.6617657	0.027659575	0.11195323
GO_NEGATIVE_REGULATION_OF_INTRINSIC_APOPTOTIC_SIGNALING_PATHWAY_BY_P53_CLASS_MEDIAT OR	18	0.5776873	1.660324	0.008064516	0.11282457
GO_MATURATION_OF_SSU_RRNA_FROM_TRICISTRONIC_RRNA_TRANSCRIPT_SSU_RRNA_5_8S_RRNA_L SU_RRNA_	33	0.6593365	1.6602219	0.027484143	0.1124324
GO_REGULATION_OF_DOUBLE_STRAND_BREAK_REPAIR_VIA_HOMOLOGOUS_RECOMBINATION	16	0.62631017	1.6599126	0.012552301	0.112225205
GO_NEGATIVE_REGULATION_OF_CYTOSKELETON_ORGANIZATION	212	0.45684937	1.659608	0.00621118	0.11206614
GO_CYTOPLASMIC_TRANSLATION	40	0.674575	1.6594002	0.05	0.11182244
GO_NCRNA_PROCESSING	371	0.55377424	1.6589243	0.046121594	0.11179535
GO_POSITIVE_REGULATION_OF_CELL_DIVISION	129	0.41216528	1.6579272	0.004464286	0.11228066
GO_TRNA_METABOLIC_PROCESS	168	0.5328607	1.6576976	0.04453441	0.11209809
GO_GENE_SILENCING_BY_RNA	132	0.4748704	1.6576579	0.012605042	0.11166818
GO_MITOCHONDRION_ORGANIZATION	554	0.45329762	1.6568488	0.030674847	0.11190521
GO_REGULATION_OF_TRANSLATIONAL_FIDELITY	14	0.66256046	1.6560736	0.022222223	0.112191744
GO_PROTEIN_SUMOYLATION	114	0.53670955	1.6559697	0.020366598	0.11185084
GO_PTERIDINE_CONTAINING_COMPOUND_BIOSYNTHETIC_PROCESS	17	0.6455646	1.6542842	0.02244898	0.1129871
GO_CENTROSOME_CYCLE	45	0.5848687	1.6542404	0.022044089	0.1125969
GO_HEMIDESMOSOME_ASSEMBLY	12	0.7616932	1.6532965	0.031380754	0.113046475
GO_PROTEIN_FOLDING	213	0.45727825	1.6531543	0.0385439	0.1127647
GO_POSITIVE_REGULATION_OF_TELOMERE_MAINTENANCE_VIA_TELOMERE_LENGTHENING	33	0.59140277	1.6509857	0.024242423	0.11453892
GO_CYTOPLASMIC_SEQUESTERING_OF_PROTEIN	40	0.5050585	1.6493893	0.022633744	0.11540048
GO_ANTIGEN_PROCESSING_AND_PRESENTATION_OF_EXOGENOUS_PEPTIDE_ANTIGEN_VIA_MHC_CLAS S_I	65	0.58247536	1.6493425	0.037815128	0.114988394
GO_INNER_CELL_MASS_CELL_PROLIFERATION	10	0.74379396	1.6480967	0.004056795	0.11565598
GO_FORMATION_OF_TRANSLATION_PREINITIATION_COMPLEX	20	0.7011439	1.6479492	0.022540983	0.11534986
GO_PROTEIN_NEDDYLATION	13	0.66709745	1.6473082	0.021186441	0.11552463
GO_REGULATION_OF_PROTEIN_MODIFICATION_BY_SMALL_PROTEIN_CONJUGATION_OR_REMOVAL	273	0.464782	1.6467485	0.00814664	0.11564379
GO_REGULATION_OF_DNA_DAMAGE_RESPONSE_SIGNAL_TRANSDUCTION_BY_P53_CLASS_MEDIATOR	28	0.55277413	1.6463332	0.012711864	0.115595415
GO_REGULATION_OF_VIRAL_RELEASE_FROM_HOST_CELL	31	0.59295875	1.6463189	0.013916501	0.115165494
GO_REGULATION_BY_VIRUS_OF_VIRAL_PROTEIN_LEVELS_IN_HOST_CELL	11	0.7313822	1.6461384	0.008179959	0.11488493
GO_HISTONE_MRNA_METABOLIC_PROCESS	28	0.6104358	1.6438702	0.020408163	0.1167043
GO_PYRIMIDINE_CONTAINING_COMPOUND_BIOSYNTHETIC_PROCESS	39	0.5239098	1.6433322	0.006651885	0.116701595
GO_POSITIVE_REGULATION_OF_DNA_REPLICATION	86	0.46408218	1.6417035	0.012631579	0.117811665
GO_REGULATION_OF_DNA_REPLICATION	157	0.46481165	1.6413407	0.01446281	0.11775781
GO_LAGGING_STRAND_ELONGATION	11	0.7205379	1.6412145	0.018255578	0.117417365
GO_EPIDERMIS_DEVELOPMENT	251	0.40702897	1.6406761	0.011848342	0.11760686
GO_NUCLEAR_TRANSCRIBED_MRNA_CATABOLIC_PROCESS_DEADENYLATION_DEPENDENT_DECAY	55	0.5646253	1.639923	0.008064516	0.11803533
GO_RNA_PHOSPHODIESTER_BOND_HYDROLYSIS_EXONUCLEOLYTIC	34	0.5772202	1.6398538	0.014	0.11765547
GO_POSITIVE_REGULATION_OF_RESPONSE_TO_DNA_DAMAGE_STIMULUS	62	0.5005591	1.6389098	0.008438818	0.11819216

GO_REGULATION_OF_PROTEIN_LOCALIZATION_TO_CHROMOSOME_TELOMERIC_REGION	14	0.71783954	1.6386812	0.029239766	0.117948964
GO_MITOCHONDRIAL_TRANSPORT	164	0.46030748	1.6375666	0.03469388	0.11859662
GO_CHAPERONE_MEDIATED_PROTEIN_FOLDING	46	0.5198293	1.6370066	0.036480688	0.118789576
GO_NEGATIVE_REGULATION_OF_CELLULAR_PROTEIN_CATABOLIC_PROCESS	60	0.5202055	1.6367737	0.006276151	0.11858529
GO_TRANSCRIPTION_FROM_RNA_POLYMERASE_I_PROMOTER	35	0.5784832	1.6346914	0.016260162	0.120059446
GO_DNA_REPLICATION_DEPENDENT_NUCLEOSOME_ORGANIZATION	32	0.594156	1.6328253	0.05458515	0.121492095
GO_TRANSLATIONAL_INITIATION	143	0.6316149	1.6323658	0.084886126	0.1215511
GO_SPONGIOTROPHOBLAST_LAYER_DEVELOPMENT	13	0.6083587	1.632331	0.02826087	0.12114935
GO_PROTEIN_LOCALIZATION_TO_CYTOSKELETON	28	0.5787906	1.6322637	0.010204081	0.1207968
GO_CLEAVAGE_INVOLVED_IN_RRNA_PROCESSING	19	0.67891294	1.6319609	0.030737706	0.12066312
GO_GENE_SILENCING	198	0.4489919	1.6313207	0.014705882	0.120854415
GO_FOLIC_ACID_METABOLIC_PROCESS	17	0.5700676	1.630616	0.02244898	0.121070765
GO_HISTONE_PHOSPHORYLATION	25	0.59341866	1.6303482	0.013015185	0.120930165
GO_ACTIVATION_OF_ANAPHASE_PROMOTING_COMPLEX_ACTIVITY	14	0.66654783	1.6300902	0.022403259	0.12071544
GO_POSITIVE_REGULATION_OF_CELL_CYCLE	321	0.4178142	1.6296318	0.00617284	0.120778345
GO_MEIOTIC_CHROMOSOME_SEGREGATION	59	0.46690586	1.6283472	0.027149322	0.121669434
GO_NEGATIVE_REGULATION_OF_ORGANELLE_ORGANIZATION	373	0.43668905	1.6274787	0.008163265	0.122104004
GO_NUCLEOTIDE_EXCISION_REPAIR_DNA_DAMAGE_RECOGNITION	23	0.62186843	1.6274781	0.019880716	0.12168296
GO_CENTRIOLE_ASSEMBLY	19	0.6235702	1.6272955	0.036885247	0.121420175
GO_EPHRIN_RECEPTOR_SIGNALING_PATHWAY	84	0.46086365	1.6263669	0.019313306	0.12191179
GO__DE_NOVO_PROTEIN_FOLDING	19	0.6387575	1.6258576	0.037190083	0.12205555
GO_RESPONSE_TO_X_RAY	30	0.55028033	1.6240091	0.012552301	0.12345899
GO_PROTEIN_LOCALIZATION_TO_CHROMATIN	13	0.6629752	1.6239226	0.023762377	0.12314838
GO_RNA_3_END_PROCESSING	96	0.5354875	1.6234496	0.025948104	0.12318276
GO_NUCLEOSIDE_TRIPHOSPHATE_METABOLIC_PROCESS	217	0.46043256	1.6225238	0.049792532	0.12376629
GO_POSITIVE_REGULATION_OF_NUCLEAR_DIVISION	61	0.4481835	1.622456	0.012793177	0.123424254
GO_POSITIVE_REGULATION_OF_CANONICAL_WNT_SIGNALING_PATHWAY	117	0.45392507	1.6224071	0.014583333	0.12306769
GO_REGULATION_OF_TELOMERE_MAINTENANCE	65	0.54234195	1.620608	0.022357723	0.12441463
GO_MEMBRANE_DISASSEMBLY	45	0.5983302	1.6203101	0.012145749	0.1242771
GO_PYRIMIDINE_NUCLEOTIDE_BIOSYNTHETIC_PROCESS	28	0.5435781	1.6186004	0.018907564	0.12564668
GO_NEGATIVE_REGULATION_OF_RECEPTOR_MEDIATED_ENDOCYTOSIS	17	0.5736048	1.617386	0.017621145	0.12645203
GO_FEMALE_MEIOTIC_DIVISION	24	0.5519421	1.6165161	0.021786492	0.12698773
GO_ORGANONITROGEN_COMPOUND_BIOSYNTHETIC_PROCESS	990	0.39255878	1.6154091	0.033826638	0.12777627
GO_CHROMATIN_REMODELING	145	0.4551741	1.6117985	0.025210084	0.13104786
GO_SOMATIC_DIVERSIFICATION_OF_IMMUNE_RECEPTORS_VIA_SOMATIC_MUTATION	12	0.66634965	1.611468	0.025052192	0.13099684
GO_ENDOPLASMIC_RETICULUM_CALCIUM_ION_HOMEOSTASIS	20	0.5683146	1.6114258	0.03909465	0.13060898
GO_REGULATION_OF_TELOMERE_MAINTENANCE_VIA_TELOMERE_LENGTHENING	49	0.5492812	1.6104445	0.027833002	0.13109685
GO_VIRAL_LIFE_CYCLE	283	0.47923476	1.6103368	0.05625	0.13078111
GO_PURINE_NUCLEOBASE_METABOLIC_PROCESS	20	0.5457782	1.6083285	0.034764826	0.1325733
GO_CELLULAR_RESPONSE_TO_EXOGENOUS_DSRNA	12	0.6279286	1.6082007	0.01898734	0.13228145
GO_KERATINOCYTE_DIFFERENTIATION	100	0.46730345	1.6079144	0.040358745	0.13210614
GO_RNA_PROCESSING	804	0.4871173	1.6044775	0.037924152	0.13527974
GO_PROTEIN_FOLDING_IN_ENDOPLASMIC_RETICULUM	8	0.7624372	1.6040546	0.039130434	0.13528039
GO_CHROMATIN_SILENCING_AT_RDNA	37	0.53959227	1.6036577	0.05869565	0.13528517

GO_REGULATION_OF_ESTABLISHMENT_OF_PROTEIN_LOCALIZATION_TO_CHROMOSOME	10	0.7669568	1.6025994	0.029296875	0.13602458
GO_RIBOSOMAL_SMALL_SUBUNIT_BIOGENESIS	56	0.61942846	1.6018542	0.05376344	0.13634804
GO_TRNA_TRANSPORT	33	0.6363494	1.6008004	0.024340771	0.13708006
GO_CYTOPLASMIC_TRANSLATIONAL_INITIATION	14	0.7204476	1.6001648	0.025157232	0.13722196
GO_NEGATIVE_REGULATION_OF_SUBSTRATE_ADHESION_DEPENDENT_CELL_SPREADING	12	0.64454997	1.6001253	0.03846154	0.13684778
GO_NEGATIVE_REGULATION_OF_BINDING	130	0.42551756	1.6000961	0.022727273	0.13645495
GO_POSITIVE_REGULATION_OF_VIRAL_TRANSCRIPTION	35	0.5302324	1.5996712	0.02734375	0.13639127
GO_NEGATIVE_REGULATION_OF_CELL_CYCLE_G1_S_PHASE_TRANSITION	94	0.46996018	1.5983187	0.02414487	0.13745455
GO_PROTEIN_TARGETING	398	0.44846377	1.5976114	0.045360826	0.13782918
GO_CHROMATIN_SILENCING	89	0.46880132	1.5972772	0.042643923	0.13778754
GO_REGULATION_OF_PROTEASOMAL_PROTEIN_CATABOLIC_PROCESS	177	0.46515867	1.5959437	0.020242915	0.13883571
GO_REGULATION_OF_MITOCHONDRIAL_OUTER_MEMBRANE_PERMEABILIZATION_INVOLVED_IN_APOPTOTIC_SIGNALING_PATHWAY	42	0.50944906	1.5957978	0.029761905	0.13855402
GO_TERMINATION_OF_RNA_POLYMERASE_II_TRANSCRIPTION	61	0.56394786	1.5956404	0.021956088	0.13827363
GO_PROTEIN_LOCALIZATION_TO_MITOCHONDRION	65	0.5114081	1.5952528	0.052525252	0.13820371
GO_TRNA_PROCESSING	108	0.51782244	1.5951381	0.05894309	0.13789539
GO_REGULATION_OF_ENDOPLASMIC_RETICULUM_UNFOLDED_PROTEIN_RESPONSE	27	0.5559191	1.5937105	0.03125	0.13896428
GO_POSITIVE_REGULATION_OF_PROTEIN_CATABOLIC_PROCESS	258	0.4348542	1.5933176	0.016260162	0.13892047
GO_PYRIMIDINE_NUCLEOTIDE_METABOLIC_PROCESS	43	0.5001171	1.5929148	0.03258656	0.13890141
GO_ORGANELLE_INHERITANCE	10	0.7226244	1.5925819	0.027613413	0.13885458
GO_RNA_DEPENDENT_DNA_BIOSYNTHETIC_PROCESS	20	0.61242217	1.5921737	0.022821577	0.13886362
GO_BLASTOCYST_DEVELOPMENT	60	0.4743266	1.5919619	0.020618556	0.13867922
GO_REGULATION_OF_MITOPHAGY	42	0.51737005	1.5904069	0.017892644	0.1399737
GO_REGULATION_OF_CELL_AGING	33	0.5077976	1.5899751	0.029106028	0.14006491
GO_HEPATOCYTE_APOPTOTIC_PROCESS	13	0.6284453	1.5893102	0.025896415	0.14025025
GO_RESPONSE_TO_GAMMA_RADIATION	50	0.47149116	1.5890555	0.031380754	0.14007652
GO_CELLULAR_RESPONSE_TO_GAMMA_RADIATION	19	0.5660698	1.5883001	0.030120483	0.14047687
GO_CYTOKINETIC_PROCESS	14	0.6248528	1.58805	0.016096579	0.14036182
GO_NEGATIVE_REGULATION_OF_PROTEIN_BINDING	78	0.4521903	1.5868472	0.026748972	0.14122596
GO_PROTEIN_DEPOLYMERIZATION	24	0.5136619	1.5866829	0.022774328	0.14099534
GO_NEGATIVE_REGULATION_OF_DNA_DAMAGE_RESPONSE_SIGNAL_TRANSDUCTION_BY_P53_CLASS_MEDIATOR	13	0.622219	1.5866578	0.035416666	0.14062527
GO_NAD_METABOLIC_PROCESS	50	0.47350103	1.5859405	0.03821656	0.14101388
GO_REGULATION_OF_PROTEIN_PHOSPHATASE_TYPE_2A_ACTIVITY	24	0.5866307	1.5857997	0.023904383	0.14074191
GO_CELLULAR_AMIDE_METABOLIC_PROCESS	700	0.40960628	1.5854009	0.053418804	0.14071782
GO_NUCLEOTIDE_EXCISION_REPAIR_DNA_DUPLEX_UNWINDING	22	0.58831346	1.5849158	0.024048096	0.1408914
GO_RNA_CATABOLIC_PROCESS	220	0.5371845	1.5844659	0.07966457	0.14102334
GO_VIRAL_LATENCY	11	0.62504584	1.5840694	0.033333335	0.14097312
GO_REGULATION_OF_TRANSLATIONAL_INITIATION	80	0.506341	1.5838306	0.026262626	0.14081226
GO_RNA_SURVEILLANCE	13	0.692264	1.5836991	0.030800821	0.14057864
GO_SNRNA_METABOLIC_PROCESS	75	0.5207528	1.5828741	0.031128405	0.14098318
GO_MULTI_ORGANISM_MEMBRANE_ORGANIZATION	30	0.5617273	1.5828017	0.03773585	0.14065894
GO_ENDOSOME_ORGANIZATION	59	0.5256305	1.5824311	0.04016064	0.1406737
GO_POSITIVE_REGULATION_OF_MITOTIC_CELL_CYCLE	122	0.44230005	1.5811651	0.026748972	0.14158994

GO_REGULATION_OF_TRANSLATIONAL_ELONGATION	22	0.5712228	1.5802245	0.028397566	0.14221674
GO_TELOMERE_MAINTENANCE_VIA_TELOMERASE	16	0.62255204	1.5768968	0.038306452	0.14551127
GO_EMBRYONIC_PLACENTA_MORPHOGENESIS	22	0.5112771	1.5762943	0.024948025	0.1457848
GO_POSITIVE_REGULATION_OF_CELL_CYCLE_PHASE_TRANSITION	67	0.49255556	1.5761569	0.027253669	0.1455381
GO_MITOCHONDRION_MORPHOGENESIS	19	0.5927601	1.5761557	0.04016064	0.14513718
GO_NUCLEOSIDE_TRIPHOSPHATE_BIOSYNTHETIC_PROCESS	57	0.4899331	1.5761284	0.057082452	0.14475194
GO_NUCLEAR_IMPORT	129	0.45581442	1.574707	0.022357723	0.14588198
GO_PYRIMIDINE_NUCLEOSIDE_TRIPHOSPHATE_BIOSYNTHETIC_PROCESS	17	0.58119744	1.5745853	0.031847134	0.14565429
GO_RESPONSE_TO_INTERFERON_BETA	22	0.5781779	1.574044	0.056338027	0.1458214
GO_MICROTUBULE_ORGANIZING_CENTER_ORGANIZATION	82	0.5133101	1.5730438	0.028571429	0.14641729
GO_PSEUDOURIDINE_SYNTHESIS	17	0.6395971	1.5726429	0.04048583	0.14642988
GO_EPIDERMAL_CELL_DIFFERENTIATION	141	0.4144382	1.5724658	0.025581395	0.14619198
GO_MEMBRANE_BUDDING	112	0.4724145	1.5715499	0.03992016	0.14678794
GO_TELOMERE_MAINTENANCE_VIA_TELOMERE_LENGTHENING	25	0.57518685	1.5698383	0.036511157	0.14826018
GO_ANTIGEN_PROCESSING_AND_PRESENTATION_OF_PEPTIDE_ANTIGEN	172	0.45528126	1.569804	0.04526749	0.14790924
GO_RNA_SPLICING_VIA_TRANSESTERIFICATION_REACTIONS	261	0.51012	1.5690255	0.030181086	0.14841086
GO_RIBONUCLEOPROTEIN_COMPLEX_LOCALIZATION	116	0.52188843	1.5679911	0.04715128	0.14922139
GO_ESTABLISHMENT_OR_MAINTENANCE_OF_BIPOLAR_CELL_POLARITY	35	0.46850136	1.567368	0.026258206	0.14952666
GO_POSITIVE_REGULATION_OF_MITOCHONDRIAL_OUTER_MEMBRANE_PERMEABILIZATION_INVOLVED_IN_APOPTOTIC_SIGNALING_PATHWAY	36	0.52516276	1.5652249	0.031936128	0.1515109
GO_NEGATIVE_REGULATION_OF_NECROTIC_CELL_DEATH	10	0.6354812	1.5651423	0.031512607	0.15120251
GO_IMP_BIOSYNTHETIC_PROCESS	11	0.6579039	1.5648502	0.043032788	0.15117213
GO_MICROTUBULE_CYTOSKELETON_ORGANIZATION	324	0.43703225	1.564712	0.03131524	0.15096769
GO_HEXOSE_CATABOLIC_PROCESS	48	0.46404567	1.5639964	0.022371365	0.15133919
GO_AEROBIC_RESPIRATION	50	0.5302928	1.563956	0.07942974	0.15098658
GO_MACROMOLECULE_CATABOLIC_PROCESS	888	0.41344407	1.5636342	0.016494846	0.15096347
GO_NEGATIVE_REGULATION_OF_CELL_CYCLE	413	0.4108412	1.56357	0.010224949	0.15064198
GO_MITOCHONDRIAL_RESPIRATORY_CHAIN_COMPLEX_ASSEMBLY	63	0.5802417	1.5628382	0.104627766	0.15108581
GO_CELLULAR_PROTEIN_COMPLEX_LOCALIZATION	22	0.5339042	1.5625414	0.025531914	0.15095669
GO_EPITHELIAL_CELL_APOPTOTIC_PROCESS	25	0.5139568	1.5615313	0.018595042	0.15154304
GO_PENTOSE_METABOLIC_PROCESS	12	0.6390608	1.5604236	0.046875	0.15235753
GO_PROTEIN_IMPORT_INTO_MITOCHONDRIAL_MATRIX	13	0.6604067	1.560142	0.062240664	0.15226544
GO_MITOCHONDRIAL_MEMBRANE_ORGANIZATION	90	0.46126264	1.560042	0.04526749	0.15198179
GO_NCRNA_3_END_PROCESSING	20	0.6232819	1.5592606	0.06369427	0.15247053
GO_GOLGI_LOCALIZATION	11	0.64478964	1.5590006	0.044806518	0.15238899
GO_CELLULAR_RESPONSE_TO_STRESS	1505	0.39090124	1.5584394	0.012658228	0.15266372
GO_MITOCHONDRIAL_ELECTRON_TRANSPORT_CYTOCHROME_C_TO_OXYGEN	16	0.6677399	1.5579678	0.09445585	0.15278412
GO_ERROR_PRONE_TRANSLESION_SYNTHESIS	19	0.61055756	1.5566612	0.034623217	0.15391776
GO_REGULATION_OF_PROTEASOMAL_UBIQUITIN_DEPENDENT_PROTEIN_CATABOLIC_PROCESS	144	0.4616643	1.5556101	0.028282829	0.1547059
GO_PYRIMIDINE_NUCLEOSIDE_TRIPHOSPHATE_METABOLIC_PROCESS	19	0.5683157	1.5554326	0.047311828	0.15452549
GO_TRNA_MODIFICATION	55	0.53642595	1.5552013	0.070564516	0.15436977
GO_CELLULAR_RESPONSE_TO_ARSENIC_CONTAINING_SUBSTANCE	14	0.5889746	1.5545489	0.052192066	0.15467556
GO_PROTEIN_UBIQUITINATION_INVOLVED_IN_UBIQUITIN_DEPENDENT_PROTEIN_CATABOLIC_PROCESS	130	0.47086424	1.554432	0.032786883	0.15445262
GO_PROTEIN_COMPLEX_SUBUNIT_ORGANIZATION	1472	0.3757891	1.5540224	0.013215859	0.15449212

GO_REGULATION_OF_DNA_DEPENDENT_DNA_REPLICATION	40	0.5456863	1.5534828	0.051759835	0.15469943
GO_PROTEIN_TRANSMEMBRANE_TRANSPORT	50	0.48659703	1.5519848	0.06198347	0.1559742
GO_RESPONSE_TO_UV	121	0.4420489	1.5516354	0.028169014	0.15596138
GO_ER_NUCLEUS_SIGNALING_PATHWAY	32	0.5530536	1.5516127	0.069767445	0.15560655
GO_POSTTRANSCRIPTIONAL_REGULATION_OF_GENE_EXPRESSION	431	0.43722954	1.550897	0.027196653	0.1560048
GO_CELL_AGING	67	0.44464937	1.5501338	0.023255814	0.15646519
GO_REGULATION_OF_PROTEIN_CATABOLIC_PROCESS	383	0.40868995	1.549618	0.02385686	0.15668575
GO_RETROGRADE_TRANSPORT_ENDOSOME_TO_PLASMA_MEMBRANE	14	0.6091372	1.5493841	0.02970297	0.1566431
GO_REGULATION_OF_CELLULAR_RESPONSE_TO_HEAT	74	0.52601004	1.5486622	0.04761905	0.15710159
GO_OXIDATIVE_PHOSPHORYLATION	81	0.58444434	1.5473527	0.13253012	0.15830076
GO_RIBONUCLEOPROTEIN_COMPLEX_SUBUNIT_ORGANIZATION	188	0.49146363	1.5466876	0.064	0.15863594
GO_NEGATIVE_REGULATION_OF_CHROMOSOME_ORGANIZATION	94	0.48971176	1.5462658	0.058232933	0.158704
GO_NEGATIVE_REGULATION_OF_MEGAKARYOCYTE_DIFFERENTIATION	18	0.5954302	1.5462632	0.06753813	0.15832974
GO_INTERSPECIES_INTERACTION_BETWEEN_ORGANISMS	646	0.42536652	1.5455391	0.021052632	0.15873803
GO_CELLULAR_RESPIRATION	138	0.5058192	1.545427	0.10229645	0.1584965
GO_MRNA_METABOLIC_PROCESS	590	0.47116327	1.5440335	0.0583501	0.15977114
GO_CELLULAR_RESPONSE_TO_IONIZING_RADIATION	50	0.48978946	1.5438718	0.05042017	0.1595537
GO_REGULATION_OF_SISTER_CHROMATID_COHESION	17	0.6117025	1.5435853	0.04233871	0.15950096
GO_GLYCERALDEHYDE_3_PHOSPHATE_METABOLIC_PROCESS	17	0.5725192	1.5435529	0.049356222	0.15915067
GO_NUCLEAR_PORE_ORGANIZATION	14	0.7047688	1.5428927	0.022357723	0.15952346
GO_REGULATION_OF_CELL_CYCLE_G1_S_PHASE_TRANSITION	142	0.4407111	1.5422535	0.018480493	0.15994358
GO_PYRIMIDINE_NUCLEOBASE_METABOLIC_PROCESS	19	0.5484239	1.5415641	0.029288704	0.1602636
GO_POSITIVE_REGULATION_OF_RELEASE_OF_CYTOCHROME_C_FROM_MITOCHONDRIA	27	0.49841425	1.5395498	0.043568466	0.16220939
GO_RESPONSE_TO_IONIZING_RADIATION	139	0.43480495	1.5387628	0.035416666	0.16276686
GO_CELLULAR_RESPONSE_TO_AMINO_ACID_STARVATION	25	0.52588487	1.5368571	0.032719836	0.16464622
GO_PROTEIN_POLYUBIQUITINATION	241	0.4554047	1.5351281	0.03	0.16622384
GO_INTRINSIC_APOPTOTIC_SIGNALING_PATHWAY_IN_RESPONSE_TO_ENDOPLASMIC_RETICULUM_STRESS	31	0.49813926	1.5348908	0.04883227	0.16607612
GO_REGULATION_OF_INTRINSIC_APOPTOTIC_SIGNALING_PATHWAY	139	0.39194217	1.5341789	0.024590164	0.16653709
GO_INTRINSIC_APOPTOTIC_SIGNALING_PATHWAY_IN_RESPONSE_TO_DNA_DAMAGE_BY_P53_CLASS_MEDIATOR	29	0.5153681	1.5337777	0.0372807	0.16661108
GO_REGULATION_OF_CENTRIOLE_REPLICATION	13	0.6356969	1.533775	0.04225352	0.16623415
GO_MUSCLE_CELL_MIGRATION	18	0.5630288	1.5336554	0.05970149	0.16599831
GO_REGULATION_OF_FIBROBLAST_GROWTH_FACTOR_RECEPTOR_SIGNALING_PATHWAY	25	0.4835207	1.5334377	0.059196617	0.16585717
GO_POSITIVE_REGULATION_OF_TRANSCRIPTION_FROM_RNA_POLYMERASE_II_PROMOTER_IN_RESPONSE_TO_STRESS	23	0.538537	1.5329794	0.07592191	0.16597195
GO_MITOCHONDRIAL_RESPIRATORY_CHAIN_COMPLEX_I_BIOGENESIS	51	0.5931561	1.5315089	0.13017751	0.1673122
GO_NEGATIVE_REGULATION_OF_DEFENSE_RESPONSE_TO_VIRUS	18	0.5742912	1.5313616	0.04742268	0.1671008
GO_NEGATIVE_REGULATION_OF_RECEPTOR_BINDING	12	0.5855992	1.5302474	0.06430155	0.16804732
GO_HETEROCHROMATIN_ORGANIZATION	11	0.59971744	1.5295434	0.064449064	0.16852069
GO_MRNA_3_END_PROCESSING	71	0.5349652	1.5295151	0.04828974	0.16815786
GO_MITOTIC_G2_DNA_DAMAGE_CHECKPOINT	13	0.62313336	1.5286505	0.05353319	0.16874343
GO_NEGATIVE_REGULATION_OF_DNA_REPAIR	13	0.6194612	1.5284623	0.039175257	0.16858019
GO_REGULATION_OF_CYCLIN_DEPENDENT_PROTEIN_KINASE_ACTIVITY	93	0.4231957	1.5284573	0.020920502	0.16821282

GO_POSITIVE_REGULATION_OF_PROTEOLYSIS	359	0.3930322	1.5283394	0.02834008	0.16795316
GO_ESTABLISHMENT_OF_MITOTIC_SPINDLE_LOCALIZATION	23	0.57097465	1.5270542	0.03805497	0.16902728
GO_MULTI_ORGANISM_METABOLIC_PROCESS	137	0.5849009	1.5248636	0.13333334	0.17129175
GO_ENDOPLASMIC_RETICULUM_ORGANIZATION	38	0.5100401	1.5235986	0.054435484	0.17244771
GO_DNA_LIGATION_INVOLVED_IN_DNA_REPAIR	12	0.6199461	1.5219305	0.04158004	0.17403997
GO_MAINTENANCE_OF_LOCATION_IN_CELL	94	0.41798985	1.5212244	0.038383838	0.17455986
GO_OOCYTE_MATURATION	18	0.5282279	1.5204601	0.044871796	0.17521882
GO_NON_CANONICAL_WNT_SIGNALING_PATHWAY	136	0.4259627	1.5199434	0.039215688	0.17552137
GO_PROTEIN_LOCALIZATION_TO_ENDOPLASMIC_RETICULUM	123	0.5859238	1.5198927	0.1446541	0.17516784
GO_NUCLEOSIDE_PHOSPHATE_BIOSYNTHETIC_PROCESS	176	0.3935531	1.5194492	0.033259425	0.17531246
GO_NEGATIVE_REGULATION_OF_KERATINOCYTE_PROLIFERATION	11	0.63567877	1.5194094	0.06889353	0.17498218
GO_NUCLEAR_TRANSCRIBED_MRNA_CATABOLIC_PROCESS_NONSENSE_MEDIATED_DECAY	118	0.6110302	1.5186458	0.12394958	0.17551385
GO_MITOCHONDRIAL_TRANSMEMBRANE_TRANSPORT	53	0.51150393	1.5181314	0.09090909	0.17582342
GO_REGULATION_OF_NUCLEASE_ACTIVITY	23	0.5285359	1.5179935	0.055785123	0.17559029
GO_NUCLEAR_TRANSCRIBED_MRNA_POLY_A_TAIL_SHORTENING	24	0.59765035	1.5173901	0.042424243	0.1759111
GO_RRNA_MODIFICATION	26	0.5619706	1.515691	0.085020244	0.17768057
GO_REGULATION_OF_CELL_CYCLE_G2_M_PHASE_TRANSITION	58	0.47179985	1.5156221	0.047210302	0.1773969
GO_CELL_ADHESION_MEDIATED_BY_INTEGRIN	12	0.6202394	1.515035	0.08387097	0.17766862
GO_CENTROSOME_LOCALIZATION	17	0.57272005	1.5141715	0.041753653	0.17831294
GO_ESTABLISHMENT_OF_PROTEIN_LOCALIZATION_TO_CHROMOSOME	12	0.645052	1.5135477	0.06483301	0.17864473
GO_MACROMOLECULAR_COMPLEX_ASSEMBLY	1344	0.37074906	1.5134256	0.017167382	0.17841312
GO_MATURATION_OF_LSU_RRNA	13	0.6626204	1.513074	0.07660455	0.17846975
GO_CELLULAR_RESPONSE_TO_INTERFERON_BETA	15	0.56751174	1.5128471	0.060728744	0.17838947
GO_INTRA_S_DNA_DAMAGE_CHECKPOINT	12	0.6365468	1.5124797	0.0373444	0.17844746
GO_NEGATIVE_REGULATION_OF_DNA_BINDING	46	0.43709004	1.512281	0.031982943	0.17833325
GO_MOLTING_CYCLE	82	0.39085853	1.510801	0.047727272	0.17973287
GO_POSITIVE_REGULATION_OF_DNA_BIOSYNTHETIC_PROCESS	58	0.46895972	1.5098438	0.05102041	0.180545
GO_REGULATION_OF_DNA_METABOLIC_PROCESS	329	0.40646434	1.5085998	0.045738045	0.18170743
GO_REGULATION_OF_CELLULAR_SENESCENCE	26	0.5117104	1.5085652	0.047311828	0.18136767
GO_NUCLEIC_ACID_PHOSPHODIESTER_BOND_HYDROLYSIS	237	0.4335418	1.5078535	0.06584362	0.18185654
GO_REGULATION_OF_CHROMOSOME_ORGANIZATION	270	0.4302104	1.5078403	0.054766733	0.18148182
GO_IMP_METABOLIC_PROCESS	14	0.6106762	1.5074241	0.06584362	0.18160848
GO_SEMAPHORIN_PLEXIN_SIGNALING_PATHWAY_INVOLVED_IN_NEURON_PROJECTION_GUIDANCE	13	0.62460417	1.5070964	0.057494868	0.18161672
GO_ERROR_FREE_TRANSLESION_SYNTHESIS	18	0.6109432	1.5069528	0.05338809	0.18140732
GO_GLYCOSYL_COMPOUND_METABOLIC_PROCESS	355	0.37844005	1.5063379	0.06355932	0.18174796
GO_SKIN_DEVELOPMENT	209	0.37679955	1.5057054	0.04195804	0.18225016
GO_NEGATIVE_REGULATION_OF_TUMOR_NECROSIS_FACTOR_MEDIATED_SIGNALING_PATHWAY	14	0.5270898	1.5054826	0.036247335	0.18214546
GO_ESTABLISHMENT_OF_PROTEIN_LOCALIZATION_TO_ENDOPLASMIC_RETICULUM	104	0.62269175	1.503305	0.15843621	0.18454817
GO_NEGATIVE_REGULATION_OF_GENE_EXPRESSION_EPIGENETIC	106	0.43300185	1.5026797	0.060085837	0.18491058
GO_POST_TRANSLATIONAL_PROTEIN_MODIFICATION	34	0.45841885	1.5026714	0.06638116	0.1845344
GO_REGULATION_OF_PROTEIN_EXPORT_FROM_NUCLEUS	33	0.48168427	1.5022527	0.0546875	0.18459988
GO_NCRNA_TRANSCRIPTION	81	0.4867627	1.5022146	0.05367793	0.18425298
GO_POSITIVE_REGULATION_OF_MULTI_ORGANISM_PROCESS	151	0.39292046	1.5012039	0.029045643	0.18512414
GO_NEGATIVE_REGULATION_OF_CANONICAL_WNT_SIGNALING_PATHWAY	159	0.4011032	1.5000222	0.050438598	0.18608472

GO_REGULATION_OF_DNA_BINDING	92	0.38619563	1.4980425	0.017699115	0.18819477
GO_CELLULAR_RESPONSE_TO_UV	65	0.44896537	1.4976366	0.04106776	0.1883516
GO_RIBONUCLEOSIDE_TRIPHOSPHATE_BIOSYNTHETIC_PROCESS	45	0.47853106	1.4973111	0.082474224	0.18828984
GO_RNA_SPLICING_VIA_ENDONUCLEOLYTIC_CLEAVAGE_AND_LIGATION	15	0.58370966	1.4968566	0.07883818	0.18848501
GO_RRNA_CATABOLIC_PROCESS	17	0.57743335	1.4950815	0.08835341	0.1902312
GO_ALDITOL_METABOLIC_PROCESS	20	0.5028575	1.4937595	0.057268724	0.19142371
GO_RIBOSOMAL_LARGE_SUBUNIT_BIOGENESIS	48	0.5669852	1.4929674	0.15113872	0.19198897
GO_ELECTRON_TRANSPORT_CHAIN	92	0.53031886	1.4927338	0.14285715	0.19193782
GO_CELL_SUBSTRATE_JUNCTION_ASSEMBLY	40	0.4952055	1.4926171	0.07048458	0.1917013
GO_INNATE_IMMUNE_RESPONSE_ACTIVATING_CELL_SURFACE_RECEPTOR_SIGNALING_PATHWAY	106	0.46091464	1.4920572	0.056565657	0.19205773
GO_CELLULAR_SENESCENCE	33	0.4568801	1.491538	0.0491453	0.1923158
GO_REGULATION_OF_APOPTOTIC_SIGNALING_PATHWAY	354	0.36081424	1.4915352	0.028688524	0.19193213
GO_NUCLEAR_PORE_COMPLEX_ASSEMBLY	10	0.70784354	1.4912796	0.04918033	0.1918406
GO_KERATINIZATION	50	0.52368057	1.4912466	0.0995671	0.19149546
GO_REGULATION_OF_CHROMATIN_SILENCING	21	0.543905	1.4909911	0.09465021	0.19144797
GO_LUNG_EPITHELIUM_DEVELOPMENT	34	0.4672559	1.4901252	0.057446808	0.1921905
GO_MITOCHONDRIAL_RNA_PROCESSING	12	0.63876474	1.489744	0.07862903	0.19229023
GO_MICROTUBULE_POLYMERIZATION_OR_DEPOLYMERIZATION	40	0.4849424	1.4892792	0.06517312	0.19249447
GO_SOMATIC_DIVERSIFICATION_OF_IMMUNOGLOBULINS	26	0.54636294	1.48913	0.08649789	0.19230746
GO_NEGATIVE_REGULATION_OF_ENDOPLASMIC_RETICULUM_UNFOLDED_PROTEIN_RESPONSE	10	0.6652538	1.4890409	0.05533597	0.19206189
GO_PROTEIN_IMPORT	153	0.41852522	1.488504	0.024948025	0.19233322
GO_FOLIC_ACID_CONTAINING_COMPOUND_METABOLIC_PROCESS	28	0.47451204	1.488491	0.06666667	0.19198042
GO_RNA_SPLICING	355	0.46885148	1.4876264	0.07692308	0.19274046
GO_IRON_ION_IMPORT	12	0.574514	1.4874718	0.059957173	0.19254595
GO_REGULATION_OF_MEMBRANE_PERMEABILITY	69	0.44457835	1.4862523	0.06762295	0.19366565
GO_REGULATION_OF_PROTEOLYSIS	694	0.33735624	1.4858111	0.01746725	0.19388075
GO_RNA_LOCALIZATION	178	0.4730566	1.484771	0.067073174	0.19484966
GO_HOMOLOGOUS_CHROMOSOME_SEGREGATION	43	0.4375008	1.4846144	0.062052507	0.1946737
GO_RRNA_CONTAINING_RIBONUCLEOPROTEIN_COMPLEX_EXPORT_FROM_NUCLEUS	11	0.6575775	1.4842494	0.070393376	0.19474292
GO_NEGATIVE_REGULATION_OF_WNT_SIGNALING_PATHWAY	192	0.38099268	1.483885	0.051948052	0.19482088
GO_RNA_PHOSPHODIESTER_BOND_HYDROLYSIS	110	0.44614342	1.4834741	0.083333336	0.19496189
GO_EARLY_ENDOSOME_TO_GOLGI_TRANSPORT	11	0.62660444	1.4829842	0.06451613	0.1951682
GO_SYNAPSIS	34	0.4589449	1.4824505	0.056650247	0.19535756
GO_REGULATION_OF_GENE_EXPRESSION_EPIGENETIC	217	0.40698326	1.4811561	0.051172707	0.19654217
GO_ESTABLISHMENT_OF_SPINDLE_ORIENTATION	25	0.5218277	1.4808928	0.05327869	0.19648266
GO_PROTEIN_TETRAMERIZATION	133	0.37001997	1.4806575	0.037647057	0.19641604
GO_VENTRICULAR_SYSTEM_DEVELOPMENT	26	0.4945192	1.4800029	0.06852248	0.19683646
GO_TETRAHYDROFOLATE_METABOLIC_PROCESS	20	0.5024933	1.4778327	0.08351178	0.1992829
GO_REGULATION_OF_ORGAN_MORPHOGENESIS	241	0.36304462	1.4767836	0.044345897	0.20021512
GO_INNER_MITOCHONDRIAL_MEMBRANE_ORGANIZATION	16	0.5830128	1.4756051	0.0951417	0.20122449
GO_NEURON_APOPTOTIC_PROCESS	34	0.4694683	1.4748383	0.057026476	0.20184852
GO_PROTEIN_EXPORT_FROM_NUCLEUS	29	0.5255518	1.4739771	0.047325104	0.20260467
GO_PROTEIN_HETEROOLIGOMERIZATION	112	0.3709261	1.4736912	0.038636364	0.2026625
GO_POSITIVE_REGULATION_OF_TELOMERASE_ACTIVITY	28	0.51299244	1.4733267	0.057142857	0.20273837

GO_SINGLE_ORGANISM_MEMBRANE_BUDDING	71	0.46066266	1.473266	0.08266129	0.20244548
GO_NEGATIVE_REGULATION_OF_DNA_TEMPLATED_TRANSCRIPTION_ELONGATION	10	0.61814266	1.4728879	0.0782241	0.2026123
GO_PLACENTA_DEVELOPMENT	136	0.3719189	1.472862	0.04077253	0.20226535
GO_NUCLEOTIDE_EXCISION_REPAIR_PREINCISION_COMPLEX_STABILIZATION	21	0.5603863	1.4720036	0.047034767	0.2030191
GO_NUCLEAR_EXPORT	143	0.4790644	1.4716072	0.09218437	0.20314384
GO_PROTEIN_CATABOLIC_PROCESS	552	0.39969206	1.4711798	0.059917357	0.20330524
GO_REGULATION_OF_IRE1_MEDIATED_UNFOLDED_PROTEIN_RESPONSE	13	0.6109477	1.4710661	0.08436214	0.20307177
GO_REGULATION_OF_RELEASE_OF_CYTOCHROME_C_FROM_MITOCHONDRIA	43	0.44566274	1.4701965	0.07053942	0.20392127
GO_RNA_MODIFICATION	112	0.46641412	1.468932	0.10887097	0.20522493
GO_NEGATIVE_REGULATION_OF_INTRINSIC_APOPTOTIC_SIGNALING_PATHWAY	84	0.3942602	1.4685829	0.04375	0.20535375
GO_RNA_POLYADENYLATION	29	0.54238045	1.4684963	0.070393376	0.20508876
GO_SNRNA_PROCESSING	21	0.5797777	1.4679121	0.09147609	0.20549107
GO_CELLULAR_RESPONSE_TO_RADIATION	134	0.4012889	1.4676086	0.04680851	0.20551635
GO_PYRIMIDINE_CONTAINING_COMPOUND_SALVAGE	12	0.5575156	1.4673659	0.06530612	0.20540048
GO_REGULATION_OF_PROTEIN_INSERTION_INTO_MITOCHONDRIAL_MEMBRANE_INVOLVED_IN_APOPTOTIC_SIGNALING_PATHWAY	29	0.50916857	1.4651835	0.06122449	0.2079532
GO_POSITIVE_REGULATION_OF_TRANSCRIPTION_FROM_RNA_POLYMERASE_II_PROMOTER_IN_RESPONSE_TO_ENDOPLASMIC_RETICULUM_STRESS	12	0.5878166	1.4651124	0.085653104	0.20766713
GO_OXIDOREDUCTION_COENZYME_METABOLIC_PROCESS	99	0.3975	1.4629766	0.06889353	0.21020266
GO_UTP_METABOLIC_PROCESS	10	0.57772774	1.4616547	0.06680162	0.21148053
GO_TRNA_THREONYLCARBAMOYLADENOSINE_METABOLIC_PROCESS	10	0.6488712	1.4593706	0.08179959	0.21417874
GO_REGULATION_OF_CYSTEINE_TYPE_ENDOPEPTIDASE_ACTIVITY	210	0.35909238	1.4585828	0.032608695	0.21483399
GO_PROTEIN_COMPLEX_BIOGENESIS	1090	0.34844548	1.4574643	0.01978022	0.2159715
GO_ER_ASSOCIATED_UBIQUITIN_DEPENDENT_PROTEIN_CATABOLIC_PROCESS	60	0.45484906	1.4571611	0.09330629	0.215973
GO_INTRINSIC_APOPTOTIC_SIGNALING_PATHWAY	150	0.37742627	1.4571247	0.036511157	0.21563792
GO_POSITIVE_REGULATION_OF_APOPTOTIC_SIGNALING_PATHWAY	169	0.38517627	1.4571152	0.047717843	0.21525581
GO_GLUCOSE_6_PHOSPHATE_METABOLIC_PROCESS	21	0.49969208	1.456996	0.075	0.21502712
GO_NEGATIVE_REGULATION_OF_TELOMERE_MAINTENANCE_VIA_TELOMERASE	11	0.6402931	1.4560418	0.07258064	0.21586408
GO_RETROGRADE_PROTEIN_TRANSPORT_ER_TO_CYTOSOL	15	0.5572315	1.4559267	0.10663984	0.21563052
GO_REGULATION_OF_UBIQUITIN_PROTEIN_LIGASE_ACTIVITY	17	0.5713142	1.4555545	0.059670784	0.21573532
GO_NUCLEAR_TRANSPORT	348	0.40952337	1.4550376	0.057142857	0.21605115
GO_DNA_DEPENDENT_DNA_REPLICATION_MAINTENANCE_OF_FIDELITY	21	0.5674606	1.4536737	0.09504132	0.21750094
GO_DNA_DOUBLE_STRAND_BREAK_PROCESSING	20	0.5471035	1.4527555	0.08418891	0.21832769
GO_NEGATIVE_REGULATION_OF_CELL_CYCLE_G2_M_PHASE_TRANSITION	24	0.51811874	1.4516387	0.08074534	0.21944042
GO_REGULATION_OF_CYTOSKELETON_ORGANIZATION	483	0.3616694	1.4514031	0.04192872	0.21938741
GO_GUANOSINE_CONTAINING_COMPOUND_METABOLIC_PROCESS	44	0.42632088	1.4510466	0.058467742	0.219484
GO_KERATAN_SULFATE_BIOSYNTHETIC_PROCESS	27	0.46947467	1.4504627	0.0824295	0.2198553
GO_MULTIVESICULAR_BODY_SORTING_PATHWAY	12	0.60667384	1.4482514	0.08213552	0.22245868
GO_TUMOR_NECROSIS_FACTOR_MEDIATED_SIGNALING_PATHWAY	117	0.41685018	1.4472725	0.09148936	0.22347729
GO_CELLULAR_METABOLIC_COMPOUND_SALVAGE	37	0.45077518	1.4471354	0.075203255	0.22329395
GO_NEGATIVE_REGULATION_OF_PEPTIDYL_TYROSINE_PHOSPHORYLATION	38	0.43080264	1.4470136	0.056112226	0.22305422
GO_CARBOHYDRATE_CATABOLIC_PROCESS	109	0.37255985	1.4464288	0.04587156	0.22342595
GO_PROTEIN_K63_LINKED_UBIQUITINATION	35	0.49382013	1.4460695	0.09406953	0.22357745
GO_PTERIDINE_CONTAINING_COMPOUND_METABOLIC_PROCESS	35	0.44051778	1.4453897	0.087248325	0.22409159

GO_UV_PROTECTION	12	0.5691848	1.4438189	0.06458333	0.2257971
GO_NEGATIVE_REGULATION_OF_CELLULAR_COMPONENT_ORGANIZATION	664	0.3575015	1.4437197	0.042918455	0.22553395
GO_NEGATIVE_REGULATION_OF_HEMATOPOIETIC_PROGENITOR_CELL_DIFFERENTIATION	24	0.5132615	1.4435034	0.10643016	0.22542474
GO_RNA_SECONDARY_STRUCTURE_UNWINDING	40	0.50787556	1.4423504	0.098121084	0.22657688
GO_MICROTUBULE_BASED_PROCESS	486	0.38647392	1.4421577	0.067653276	0.22640604
GO_POSITIVE_REGULATION_OF_WNT_SIGNALING_PATHWAY	150	0.38947695	1.441331	0.07112971	0.22717477
GO_GENERATION_OF_PRECURSOR_METABOLITES_AND_ENERGY	281	0.3831179	1.4404978	0.10537634	0.22795004
GO_REGULATION_OF_TRANSCRIPTION_ELONGATION_FROM_RNA_POLYMERASE_II_PROMOTER	21	0.5538261	1.4403216	0.07272727	0.2278075
GO_REGULATION_OF_ORGANELLE_ORGANIZATION	1131	0.36017844	1.4402056	0.05394191	0.22756171
GO_NEURAL_TUBE_FORMATION	94	0.41673422	1.4397032	0.08955224	0.22786528
GO_NEGATIVE_REGULATION_OF_PROTEIN_CATABOLIC_PROCESS	104	0.39640492	1.4392163	0.06854839	0.22813666
GO_EMBRYONIC_PLACENTA_DEVELOPMENT	83	0.39085948	1.43858	0.05764967	0.2286164
GO_VENTRICULAR_CARDIAC_MUSCLE_CELL_DEVELOPMENT	13	0.50955546	1.4384964	0.07692308	0.22835054
GO_PROTEIN_HYDROXYLATION	15	0.5507586	1.4380302	0.10262009	0.2285407
GO_RESPONSE_TO_INTERFERON_ALPHA	20	0.5538519	1.4379902	0.14107884	0.22818534
GO_SNORNA_METABOLIC_PROCESS	11	0.62633044	1.4378388	0.10878661	0.2279992
GO_REGULATION_OF_VIRAL_INDUCED_CYTOPLASMIC_PATTERN_RECOGNITION_RECEPTOR_SIGNALING_PATHWAY	15	0.53910124	1.4377257	0.09453782	0.22775784
GO_PROTEIN_EXIT_FROM_ENDOPLASMIC_RETICULUM	19	0.54391444	1.4375317	0.10973085	0.22761299
GO_REGULATION_OF_TRANSCRIPTION_FROM_RNA_POLYMERASE_I_PROMOTER	23	0.5013259	1.4373362	0.085365854	0.22751053
GO_RELEASE_OF_CYTOCHROME_C_FROM_MITOCHONDRIA	22	0.4873981	1.437061	0.12896407	0.22747451
GO_RESPONSE_TO_INCREASED_OXYGEN_LEVELS	23	0.45457274	1.4367915	0.0809628	0.22747615
GO_POSITIVE_REGULATION_OF_DNA_TEMPLATED_TRANSCRIPTION_ELONGATION	22	0.5418124	1.4359347	0.086	0.22826068
GO_REGULATION_OF_CYSTEINE_TYPE_ENDOPEPTIDASE_ACTIVITY_INVOLVED_IN_APOPTOTIC_SIGNALING_PATHWAY	22	0.5082441	1.4350964	0.11089109	0.22899482
GO_PHARYNGEAL_SYSTEM_DEVELOPMENT	17	0.4973697	1.4350053	0.09799554	0.22875942
GO_SPINDLE_LOCALIZATION	37	0.49652103	1.4341915	0.07484408	0.22948314
GO_RESPONSE_TO_TYPE_I_INTERFERON	67	0.4771617	1.4333689	0.1411043	0.23015103
GO_POSITIVE_REGULATION_OF_EXTRINSIC_APOPTOTIC_SIGNALING_PATHWAY	51	0.42312166	1.433278	0.07933194	0.22987813
GO_IN_UTERO_EMBRYONIC_DEVELOPMENT	307	0.36661145	1.4327234	0.06485356	0.23022531
GO_PYRIMIDINE_NUCLEOSIDE_METABOLIC_PROCESS	47	0.41960624	1.4321733	0.05172414	0.23053145
GO_RESPONSE_TO_TOPOLOGICALLY_INCORRECT_PROTEIN	158	0.3997333	1.4309685	0.13502109	0.23181802
GO_CARBOHYDRATE_BIOSYNTHETIC_PROCESS	120	0.3617729	1.4309012	0.05032823	0.23152944
GO_NUCLEOTIDE_SUGAR_METABOLIC_PROCESS	33	0.4780291	1.4298053	0.116228074	0.23259605
GO_POSITIVE_REGULATION_OF_TRANSCRIPTION_ELONGATION_FROM_RNA_POLYMERASE_II_PROMOTER	13	0.5997665	1.4280106	0.08678501	0.23462704
GO_MITOCHONDRIAL_ELECTRON_TRANSPORT_UBIQUINOL_TO_CYTOCHROME_C	13	0.6326901	1.427622	0.14693877	0.2347817
GO_POSITIVE_REGULATION_OF_LAMELLIPODIUM_ASSEMBLY	15	0.5307552	1.4276093	0.10548523	0.23441756
GO_POSITIVE_REGULATION_OF_PROTEASOMAL_PROTEIN_CATABOLIC_PROCESS	98	0.41793364	1.4266988	0.09475806	0.23531146
GO_REGULATION_OF_SYMBIOSIS_ENCOMPASSING_MUTUALISM_THROUGH_PARASITISM	197	0.3803984	1.4260347	0.07942974	0.23579445
GO_NEGATIVE_REGULATION_OF_CYSTEINE_TYPE_ENDOPEPTIDASE_ACTIVITY	87	0.36800772	1.4245623	0.05668934	0.23739609
GO_CELLULAR_CARBOHYDRATE_METABOLIC_PROCESS	136	0.36185429	1.4245013	0.057569295	0.23707862
GO_GLYCOPROTEIN_CATABOLIC_PROCESS	15	0.5134987	1.4240898	0.09151786	0.23729669
GO_VESICLE_MEDIATED_TRANSPORT_BETWEEN_ENDOSOMAL_COMPARTMENTS	19	0.48755735	1.4238993	0.08074534	0.23717298

GO_POSITIVE_REGULATION_OF_VIRAL_GENOME_REPLICATION	30	0.48889267	1.4229779	0.107942976	0.23808959
GO_PYRIMIDINE_DEOXYRIBONUCLEOTIDE_METABOLIC_PROCESS	16	0.539462	1.4225523	0.09484536	0.23831993
GO__DE_NOVO_POSTTRANSLATIONAL_PROTEIN_FOLDING	14	0.59737015	1.422233	0.12288135	0.23835641
GO_VESICLE_TARGETING	76	0.44035625	1.4214916	0.0995935	0.23895232
GO_RESPONSE_TO_COPPER_ION	27	0.42024258	1.4207448	0.06966292	0.23958413
GO_ORGAN_REGENERATION	82	0.3648199	1.4196901	0.062222224	0.24064414
GO_XENOPHAGY	102	0.36214796	1.4183961	0.049327355	0.24206313
GO_CELL_JUNCTION_ASSEMBLY	127	0.4021139	1.4183797	0.10089686	0.24169518
GO_ESTABLISHMENT_OR_MAINTENANCE_OF_MONOPOLAR_CELL_POLARITY	14	0.5201075	1.4183027	0.09110629	0.24141425
GO_RIBOSOME_ASSEMBLY	51	0.5295766	1.418097	0.17547569	0.24131612
GO_CYTOPLASMIC_PATTERN_RECOGNITION_RECEPTOR_SIGNALING_PATHWAY	32	0.49847338	1.4180194	0.108606555	0.2410393
GO_ANTIGEN_PROCESSING_AND_PRESENTATION	208	0.39964935	1.4174423	0.12215321	0.2414706
GO_NEGATIVE_REGULATION_OF_TRANSFERASE_ACTIVITY	344	0.35006714	1.4170276	0.049484536	0.2416179
GO_POSITIVE_REGULATION_OF_CHROMOSOME_ORGANIZATION	146	0.41882142	1.4169807	0.10483871	0.24130231
GO_ESTABLISHMENT_OF_PROTEIN_LOCALIZATION_TO_MEMBRANE	262	0.4132945	1.4163158	0.14437367	0.24186458
GO_REGULATION_OF_MEIOTIC_CELL_CYCLE	40	0.38980982	1.415836	0.073913045	0.24209775
GO_ENERGY_DERIVATION_BY_OXIDATION_OF_ORGANIC_COMPOUNDS	209	0.40071943	1.4155234	0.1368421	0.24216908
GO_REGULATION_OF_LAMELLIPODIUM_ASSEMBLY	25	0.47570863	1.4149483	0.08955224	0.24251461
GO_CELLULAR_CARBOHYDRATE_BIOSYNTHETIC_PROCESS	50	0.4084888	1.4149425	0.06320542	0.24214344
GO_NEGATIVE_REGULATION_OF_DNA_REPLICATION	54	0.44962394	1.4137537	0.11632653	0.24332906
GO_FC_EPSILON_RECEPTOR_SIGNALING_PATHWAY	124	0.43849242	1.4136603	0.11155379	0.24310654
GO_REGULATION_OF_CANONICAL_WNT_SIGNALING_PATHWAY	231	0.35564774	1.412885	0.06113537	0.24383551
GO_SOMATIC_RECOMBINATION_OF_IMMUNOGLOBULIN_GENE_SEGMENTS	21	0.5353969	1.4124781	0.11578947	0.24400632
GO_CELLULAR_PROTEIN_COMPLEX_ASSEMBLY	322	0.36791855	1.4111583	0.064935066	0.24545921
GO_CELLULAR_MODIFIED_AMINO_ACID_BIOSYNTHETIC_PROCESS	51	0.3933611	1.4106404	0.09544469	0.24579279
GO_NUCLEOBASE_CONTAINING_COMPOUND_TRANSPORT	192	0.42502254	1.4099041	0.126294	0.24653637
GO_REGULATION_OF_MITOCHONDRION_ORGANIZATION	205	0.36713192	1.4093359	0.07515658	0.24690057
GO_INTRACELLULAR_PROTEIN_TRANSPORT	760	0.37135062	1.4093143	0.07157464	0.24653347
GO_MAINTENANCE_OF_PROTEIN_LOCALIZATION_IN_ORGANELLE	29	0.45176756	1.4092094	0.093495935	0.24628109
GO_REGULATION_OF_OLIGODENDROCYTE_DIFFERENTIATION	28	0.42844018	1.4091908	0.08908686	0.24591477
GO_MITOCHONDRIAL_RNA_METABOLIC_PROCESS	24	0.553498	1.4091585	0.13195877	0.24558549
GO_REGULATION_OF_GENE_SILENCING	51	0.43264243	1.408258	0.10606061	0.24644935
GO_NEGATIVE_REGULATION_OF_TELOMERE_MAINTENANCE_VIA_TELOMERE_LENGTHENING	16	0.5693925	1.4056624	0.115234375	0.24963623
**Cellular component (203 terms)**					
GO_SPINDLE_MIDZONE	26	0.84104335	2.3260758	0	0
GO_CONDENSED_CHROMOSOME	184	0.625094	2.0458548	0	0.01949567
GO_NUCLEOTIDE_EXCISION_REPAIR_COMPLEX	14	0.7983497	2.0102675	0	0.038233623
GO_CONDENSED_CHROMOSOME_CENTROMERIC_REGION	94	0.6848244	1.9877609	0	0.038488626
GO_KINETOCHORE	112	0.657697	1.9568988	0	0.04419017
GO_CONDENSED_NUCLEAR_CHROMOSOME	80	0.584971	1.9561487	0	0.036825143
GO_MITOTIC_SPINDLE	51	0.6454512	1.9554551	0	0.03201563
GO_REPLICATION_FORK	62	0.6866982	1.9434314	0.001972387	0.03494824
GO_CHROMOSOMAL_REGION	319	0.59015507	1.9427758	0	0.031548277
GO_CONDENSED_NUCLEAR_CHROMOSOME_CENTROMERIC_REGION	16	0.8323831	1.9366175	0	0.030137176

GO_PROTEASOME_COMPLEX	76	0.66312754	1.9265217	0.007858546	0.030978143
GO_CHROMOCENTER	13	0.7435613	1.9160497	0.002123142	0.032441642
GO_ORGANELLE_ENVELOPE_LUMEN	76	0.5594049	1.9022834	0.004040404	0.036134213
GO_CHROMOSOME_CENTROMERIC_REGION	166	0.6220622	1.9008061	0	0.034466125
GO_SPINDLE_MICROTUBULE	57	0.6339331	1.9004064	0	0.032366376
GO_REPLISOME	29	0.69838977	1.892537	0.001941748	0.033068
GO_METHYLOSOME	12	0.89705026	1.8873161	0	0.033075836
GO_MIDBODY	129	0.5590375	1.8792971	0	0.03451685
GO_SPINDLE	275	0.54570806	1.8760564	0	0.03406348
GO_CHROMOSOME	849	0.51045597	1.85601	0	0.041126177
GO_NUCLEAR_CHROMOSOME	506	0.49960688	1.8502165	0.00203666	0.0412078
GO_CHROMOSOME_TELOMERIC_REGION	158	0.54874	1.8380488	0.002057613	0.046140872
GO_XY_BODY	15	0.71142715	1.8375856	0.002004008	0.04421172
GO_NUCLEAR_REPLICATION_FORK	39	0.66956687	1.8347232	0.002016129	0.043765962
GO_NUCLEAR_CHROMOSOME_TELOMERIC_REGION	128	0.5549181	1.8295096	0.004115226	0.04469737
GO_PROTEIN_DNA_COMPLEX	171	0.5284183	1.8286493	0.006237006	0.043512262
GO_SMN_SM_PROTEIN_COMPLEX	17	0.75621843	1.8266293	0.004115226	0.04252426
GO_PROTEASOME_ACCESSORY_COMPLEX	24	0.7513342	1.8184818	0.00204499	0.045735843
GO_SPINDLE_POLE	116	0.5731225	1.8079107	0.002	0.051044542
GO_U1_SNRNP	17	0.7229952	1.7972847	0.008130081	0.055034705
GO_CONDENSED_CHROMOSOME_OUTER_KINETOCHORE	11	0.9060836	1.7920536	0.004016064	0.056582082
GO_COPI_COATED_VESICLE_MEMBRANE	16	0.7472838	1.783408	0.003944773	0.059525277
GO_ENDOPLASMIC_RETICULUM_GOLGI_INTERMEDIATE_COMPARTMENT_MEMBRANE	58	0.5610592	1.7821074	0.010351967	0.058385517
GO_DNA_PACKAGING_COMPLEX	104	0.5660737	1.7751032	0.02244898	0.060003523
GO_PRECATALYTIC_SPLICEOSOME	21	0.7182518	1.7679787	0.009881423	0.062912434
GO_ORGANELLAR_RIBOSOME	69	0.6611375	1.7659861	0.024340771	0.062298488
GO_NUCLEAR_PORE	74	0.59543335	1.7587587	0.00984252	0.06515336
GO_PRONUCLEUS	15	0.7300796	1.7550066	0.00203252	0.065573655
GO_ORGANELLAR_LARGE_RIBOSOMAL_SUBUNIT	31	0.6932568	1.7451748	0.028	0.07033719
GO_ORGANELLAR_SMALL_RIBOSOMAL_SUBUNIT	25	0.69598883	1.7440757	0.03	0.06922811
GO_PRERIBOSOME	59	0.6383732	1.7440171	0.018518519	0.067539625
GO_INTRINSIC_COMPONENT_OF_MITOCHONDRIAL_INNER_MEMBRANE	17	0.6711382	1.7391809	0.009784736	0.06890651
GO_TELOMERASE_HOLOENZYME_COMPLEX	19	0.6935331	1.7383791	0.014198783	0.06787225
GO_SPLICEOSOMAL_TRI_SNRNP_COMPLEX	26	0.6877061	1.7232084	0.033009708	0.076120496
GO_COPI_VESICLE_COAT	12	0.745048	1.7228241	0.006012024	0.07458845
GO_RIBONUCLEOPROTEIN_COMPLEX	693	0.5154228	1.7174256	0.020120725	0.076408155
GO_ENDOPLASMIC_RETICULUM_GOLGI_INTERMEDIATE_COMPARTMENT	97	0.501618	1.7138463	0.008048289	0.076830775
GO_DNA_REPAIR_COMPLEX	38	0.5748586	1.7115395	0.008032128	0.07716386
GO_FILAMENTOUS_ACTIN	20	0.599922	1.6987783	0.016771488	0.08463159
GO_NUCLEOID	40	0.59771866	1.6982188	0.021956088	0.08314815
GO_ESCRT_COMPLEX	24	0.6488868	1.6947006	0.012170386	0.08428198
GO_ESCRT_III_COMPLEX	11	0.74569863	1.686286	0.010080645	0.08892367
GO_KINESIN_COMPLEX	54	0.5088976	1.6823125	0.014112903	0.090632655
GO_SMALL_NUCLEAR_RIBONUCLEOPROTEIN_COMPLEX	59	0.578973	1.6813229	0.027613413	0.089919195

GO_NUCLEOLAR_PART	61	0.590519	1.6805143	0.020833334	0.08898666
GO_SITE_OF_DOUBLE_STRAND_BREAK	31	0.5890624	1.6788667	0.023483366	0.08832552
GO_DESMOSOME	26	0.5904874	1.6775085	0.01754386	0.08793189
GO_CYTOPLASMIC_EXOSOME_RNASE_COMPLEX_	15	0.68985546	1.6749554	0.00811359	0.08796088
GO_CUL4_RING_E3_UBIQUITIN_LIGASE_COMPLEX	25	0.6250275	1.674746	0.00984252	0.08664821
GO_RIBOSOME	217	0.60081834	1.6742573	0.074152544	0.08562632
GO_NUCLEOLUS	820	0.469929	1.6734222	0.02008032	0.08486685
GO_U12_TYPE_SPLICEOSOMAL_COMPLEX	25	0.641022	1.664671	0.027944112	0.089238115
GO_METHYLTRANSFERASE_COMPLEX	83	0.55210423	1.6615765	0.010060363	0.090183884
GO_DNA_DIRECTED_RNA_POLYMERASE_I_COMPLEX	13	0.678317	1.6611584	0.018595042	0.08910698
GO_90S_PRERIBOSOME	23	0.65391266	1.649779	0.018789144	0.096765004
GO_HOLO_TFIIH_COMPLEX	12	0.7022134	1.6420639	0.028225806	0.10165976
GO_MITOCHONDRIAL_PROTEIN_COMPLEX	130	0.5698359	1.6396762	0.070393376	0.10252093
GO_CATALYTIC_STEP_2_SPLICEOSOME	86	0.57532	1.6386323	0.031189084	0.10166273
GO_ORGANELLE_INNER_MEMBRANE	498	0.45414358	1.635548	0.03773585	0.102840886
GO_NUCLEAR_NUCLEOSOME	40	0.5954632	1.6316879	0.048117153	0.10475935
GO_DNA_DIRECTED_RNA_POLYMERASE_II_HOLOENZYME	95	0.5098183	1.6307847	0.012	0.10408149
GO_SEX_CHROMOSOME	27	0.59284085	1.6248122	0.015936255	0.107553735
GO_NUCLEAR_PERIPHERY	118	0.4930427	1.61825	0.018367346	0.11191613
GO_DNA_DIRECTED_RNA_POLYMERASE_III_COMPLEX	18	0.6140867	1.6176693	0.023952097	0.11102001
GO_RIBOSOMAL_SUBUNIT	158	0.6377086	1.6158568	0.0968421	0.11104481
GO_FANCONI_ANAEMIA_NUCLEAR_COMPLEX	13	0.67521495	1.6158319	0.016359918	0.1095837
GO_NUCLEAR_EXOSOME_RNASE_COMPLEX_	15	0.6742558	1.6134888	0.02258727	0.11010733
GO_EXOSOME_RNASE_COMPLEX_	21	0.6221308	1.6128706	0.022177419	0.10920379
GO_CHROMATIN	430	0.4313483	1.6126702	0.01048218	0.10792079
GO_CARBOXY_TERMINAL_DOMAIN_PROTEIN_KINASE_COMPLEX	22	0.6263262	1.6096051	0.014492754	0.10906407
GO_NUCLEAR_CHROMATIN	285	0.42781344	1.6067563	0.008281574	0.110226825
GO_RNA_POLYMERASE_COMPLEX	120	0.49278396	1.6023597	0.027722772	0.11247446
GO_PERICENTRIC_HETEROCHROMATIN	14	0.67543846	1.602022	0.023904383	0.111368574
GO_EUKARYOTIC_TRANSLATION_INITIATION_FACTOR_3_COMPLEX	16	0.6755767	1.6000555	0.031936128	0.11198203
GO_U2_SNRNP	18	0.6504549	1.6000228	0.040650405	0.11066459
GO_ANAPHASE_PROMOTING_COMPLEX	20	0.6247281	1.5992827	0.015936255	0.110066384
GO_SMALL_SUBUNIT_PROCESSOME	33	0.60705775	1.597637	0.04192872	0.11036505
GO_NUCLEAR_UBIQUITIN_LIGASE_COMPLEX	40	0.52782065	1.5963944	0.02918288	0.11022969
GO_MITOCHONDRIAL_MATRIX	397	0.44345337	1.5951979	0.045360826	0.11008649
GO_NUCLEAR_HETEROCHROMATIN	32	0.5408817	1.5940279	0.024390243	0.109908015
GO_MITOCHONDRIAL_PART	900	0.41722912	1.5907418	0.052738335	0.11160001
GO_EXTRINSIC_COMPONENT_OF_ORGANELLE_MEMBRANE	25	0.5463017	1.590667	0.017274473	0.11041337
GO_ARP2_3_PROTEIN_COMPLEX	11	0.6767838	1.5901756	0.037401576	0.109803736
GO_DNA_DIRECTED_RNA_POLYMERASE_II_CORE_COMPLEX	16	0.5928018	1.5896355	0.025742574	0.108954884
GO_SMALL_RIBOSOMAL_SUBUNIT	67	0.63669467	1.588632	0.10782241	0.10863769
GO_ENVELOPE	1034	0.39451194	1.5882437	0.01778656	0.10787465
GO_U4_U6_X_U5_TRI_SNRNP_COMPLEX	21	0.6486582	1.5877584	0.061023623	0.10709463
GO_MITOCHONDRIAL_ENVELOPE	651	0.4246095	1.5872039	0.049586777	0.10661438

GO_TRANSCRIPTION_FACTOR_TFTC_COMPLEX	14	0.6385319	1.5843153	0.020449897	0.10783634
GO_INTRINSIC_COMPONENT_OF_MITOCHONDRIAL_MEMBRANE	44	0.5077217	1.5821255	0.059760958	0.108412735
GO_COHESIN_COMPLEX	11	0.6819116	1.5775199	0.04112554	0.11088169
GO_DNA_POLYMERASE_COMPLEX	13	0.6344448	1.5756717	0.03846154	0.11123737
GO_MLL1_2_COMPLEX	26	0.5921828	1.5754778	0.018255578	0.11033714
GO_CELL_SUBSTRATE_JUNCTION	393	0.43214065	1.574573	0.04592902	0.110123545
GO_LATERAL_ELEMENT	10	0.64834774	1.572871	0.05605381	0.11041852
GO_PROTEASOME_REGULATORY_PARTICLE_BASE_SUBCOMPLEX	12	0.7154797	1.5725623	0.026156941	0.10965078
GO_COPI_COATED_VESICLE	22	0.5840315	1.5723255	0.046692606	0.10884525
GO_CYTOSOLIC_PROTEASOME_COMPLEX	11	0.7212852	1.5721248	0.026209677	0.10805043
GO_SPLICEOSOMAL_COMPLEX	165	0.51497704	1.5676765	0.05882353	0.11020962
GO_HETEROCHROMATIN	66	0.48235494	1.5633675	0.018556701	0.11293165
GO_PRERIBOSOME_LARGE_SUBUNIT_PRECURSOR	20	0.67208725	1.5618517	0.04621849	0.11303397
GO_CELL_DIVISION_SITE	52	0.478522	1.5618477	0.026584867	0.112032674
GO_LARGE_RIBOSOMAL_SUBUNIT	91	0.6388327	1.5562295	0.10683761	0.11507954
GO_EUCHROMATIN	30	0.5025108	1.5548385	0.030864198	0.11530045
GO_PIGMENT_GRANULE	101	0.45336327	1.5444121	0.055785123	0.12283112
GO_SMN_COMPLEX	12	0.6385872	1.541536	0.053497944	0.124197826
GO_INNER_MITOCHONDRIAL_MEMBRANE_PROTEIN_COMPLEX	101	0.5738572	1.5405756	0.10973085	0.123900265
GO_NUCLEAR_MATRIX	95	0.47337177	1.5404632	0.024793388	0.12290171
GO_ANCHORING_JUNCTION	483	0.39881375	1.535496	0.04761905	0.12622277
GO_MITOCHONDRIAL_MEMBRANE_PART	161	0.49835575	1.5349631	0.10580913	0.12542067
GO_DNA_HELICASE_COMPLEX	14	0.64322674	1.5339364	0.036217302	0.12520336
GO_CYTOCHROME_COMPLEX	20	0.5842063	1.5322926	0.088607594	0.12542772
GO_MICROTUBULE_END	21	0.5945179	1.5303905	0.0513347	0.12607974
GO_ORGANELLE_MEMBRANE_CONTACT_SITE	11	0.6966256	1.5293269	0.027079303	0.1259721
GO_INVADOPODIUM	12	0.59802914	1.5277249	0.060344826	0.12614769
GO_CAJAL_BODY	49	0.51509035	1.5222515	0.062626265	0.1298703
GO_RESPIRATORY_CHAIN	78	0.5824595	1.5213636	0.11958763	0.12955233
GO_TRANSLATION_PREINITIATION_COMPLEX	16	0.64571077	1.5183176	0.0734127	0.13123667
GO_NUCLEAR_EUCHROMATIN	23	0.5188934	1.5174655	0.033195022	0.13102703
GO_BETA_CATENIN_DESTRUCTION_COMPLEX	14	0.5651986	1.5098767	0.033126295	0.13640216
GO_MITOCHONDRION	1546	0.37719896	1.5048269	0.059063137	0.14022863
GO_INTERCELLULAR_BRIDGE	40	0.48296908	1.503907	0.043824703	0.14003058
GO_GERM_CELL_NUCLEUS	18	0.50249916	1.5007719	0.055201698	0.1417771
GO_PROTEIN_PHOSPHATASE_TYPE_2A_COMPLEX	20	0.55312675	1.5004094	0.044806518	0.14105962
GO_VESICLE_COAT	44	0.49916184	1.5000247	0.06420233	0.14043976
GO_CYCLIN_DEPENDENT_PROTEIN_KINASE_HOLOENZYME_COMPLEX	30	0.52727294	1.4953043	0.050314464	0.14352527
GO_INO80_TYPE_COMPLEX	21	0.5713214	1.4863981	0.058704454	0.15045401
GO_PRP19_COMPLEX	13	0.6547913	1.4801719	0.06990291	0.15514763
GO_PRESPLICEOSOME	21	0.54631174	1.4782227	0.08863199	0.15592083
GO_CLATHRIN_COAT_OF_ENDOCYTIC_VESICLE	12	0.58347094	1.4724025	0.06097561	0.16057241
GO_OXIDOREDUCTASE_COMPLEX	92	0.46642926	1.4689081	0.126294	0.16312899
GO_SMALL_NUCLEOLAR_RIBONUCLEOPROTEIN_COMPLEX	19	0.5963133	1.4648672	0.09876543	0.16626109

GO_DNA_REPLICATION_FACTOR_A_COMPLEX	15	0.5857178	1.4594969	0.06187625	0.17042628
GO_CORNIFIED_ENVELOPE	45	0.5670365	1.4584446	0.09567198	0.17036583
GO_GOLGI_ASSOCIATED_VESICLE_MEMBRANE	48	0.43847212	1.455886	0.057312254	0.17188369
GO_COMPACT_MYELIN	14	0.5562161	1.4535052	0.07505071	0.17300765
GO_MICROTUBULE_ORGANIZING_CENTER_PART	138	0.41464427	1.4502409	0.068136275	0.17500137
GO_CENTROSOME	467	0.39898914	1.4440563	0.074	0.17991252
GO_SYNAPTONEMAL_COMPLEX	33	0.44054693	1.4431202	0.058139537	0.17960514
GO_MICROTUBULE_ORGANIZING_CENTER	589	0.3896266	1.4401463	0.06288032	0.1814978
GO_TOR_COMPLEX	13	0.5768153	1.4379207	0.072265625	0.18276168
GO_MICROTUBULE_CYTOSKELETON	1012	0.37152585	1.4359473	0.05858586	0.1836914
GO_ENDOPLASMIC_RETICULUM_QUALITY_CONTROL_COMPARTMENT	13	0.603353	1.4357442	0.10958904	0.18267743
GO_ENDOPLASMIC_RETICULUM_CHAPERONE_COMPLEX	11	0.6550231	1.4351535	0.09856263	0.18209048
GO_MRNA_CLEAVAGE_AND_POLYADENYLATION_SPECIFICITY_FACTOR_COMPLEX	12	0.60519123	1.4257753	0.108606555	0.19079481
GO_MICROTUBULE_PLUS_END	16	0.5741014	1.424729	0.09343936	0.190661
GO_MYELIN_SHEATH	165	0.39438027	1.4230833	0.1	0.19094916
GO_NUCLEAR_CYCLIN_DEPENDENT_PROTEIN_KINASE_HOLOENZYME_COMPLEX	15	0.5912651	1.4204445	0.10204082	0.19279106
GO_PHOSPHATASE_COMPLEX	44	0.48636755	1.4184752	0.134	0.19343357
GO_CYTOSOLIC_PART	213	0.44941416	1.4160222	0.1695279	0.19505422
GO_CLATHRIN_COAT_OF_COATED_PIT	16	0.5293268	1.4157741	0.112	0.19418441
GO_CYTOPLASMIC_STRESS_GRANULE	31	0.50593203	1.4157735	0.12273642	0.19298574
GO_SCHMIDT_LANTERMAN_INCISURE	10	0.5664076	1.4141964	0.08782435	0.19345312
GO_NADH_DEHYDROGENASE_COMPLEX	42	0.587203	1.4137212	0.18609408	0.19280127
GO_INTRINSIC_COMPONENT_OF_ENDOPLASMIC_RETICULUM_MEMBRANE	120	0.39054215	1.4105048	0.08382066	0.19528994
GO_MCM_COMPLEX	11	0.6448941	1.4092671	0.1252485	0.19549616
GO_N_TERMINAL_PROTEIN_ACETYLTRANSFERASE_COMPLEX	9	0.60607094	1.4059713	0.077731095	0.19751412
GO_LATERAL_PLASMA_MEMBRANE	49	0.4071368	1.4014344	0.11214953	0.20105983
GO_CYTOPLASMIC_UBIQUITIN_LIGASE_COMPLEX	13	0.5510336	1.390843	0.08565737	0.21121041
GO_CYTOSOLIC_RIBOSOME	109	0.58284867	1.387264	0.24472573	0.21404941
GO_CYTOSOLIC_SMALL_RIBOSOMAL_SUBUNIT	43	0.62217426	1.3870012	0.20940171	0.21305254
GO_MRNA_CLEAVAGE_FACTOR_COMPLEX	17	0.560563	1.3845685	0.118609406	0.21439931
GO_CYTOSOLIC_LARGE_RIBOSOMAL_SUBUNIT	58	0.61769307	1.3824157	0.23868313	0.21563461
GO_SIN3_TYPE_COMPLEX	16	0.54448086	1.3814529	0.115234375	0.21535446
GO_RIBONUCLEOPROTEIN_GRANULE	142	0.39726046	1.3798908	0.12298387	0.2159885
GO_CYTOSKELETAL_PART	1375	0.32888514	1.3790755	0.04831933	0.21564575
GO_SEMAPHORIN_RECEPTOR_COMPLEX	11	0.5403336	1.376138	0.13821138	0.217876
GO_U5_SNRNP	16	0.5630189	1.3748124	0.15445544	0.21816726
GO_IKAPPAB_KINASE_COMPLEX	11	0.5401259	1.3737681	0.12394958	0.2180395
GO_RNA_POLYMERASE_II_TRANSCRIPTION_FACTOR_COMPLEX	101	0.38475907	1.3713241	0.116564415	0.21951196
GO_TRANSFERASE_COMPLEX	671	0.38780746	1.3692687	0.14457831	0.22069615
GO_STAGA_COMPLEX	14	0.54380524	1.3684026	0.107421875	0.22051162
GO_HISTONE_METHYLTRANSFERASE_COMPLEX	64	0.4613078	1.366467	0.16071428	0.2213981
GO_CATALYTIC_COMPLEX	995	0.36449766	1.3624283	0.13572854	0.22445054
GO_CENTRIOLE	96	0.40761477	1.3590596	0.13114753	0.22682957
GO_GAMMA_TUBULIN_COMPLEX	19	0.48909497	1.3567177	0.13095239	0.2280362

GO_MALE_GERM_CELL_NUCLEUS	14	0.47775525	1.3541172	0.12298387	0.23000774
GO_MICROTUBULE_ASSOCIATED_COMPLEX	143	0.37958485	1.3536754	0.11554622	0.22930379
GO_PROTON_TRANSPORTING_TWO_SECTOR_ATPASE_COMPLEX_CATALYTIC_DOMAIN	17	0.49567354	1.3482997	0.16227181	0.23439552
GO_CYTOPLASMIC_MICROTUBULE	52	0.402194	1.3462011	0.15913978	0.23570903
GO_PROTEIN_PHOSPHATASE_TYPE_1_COMPLEX	10	0.60121113	1.34456	0.15662651	0.2362129
GO_TRANSFERASE_COMPLEX_TRANSFERRING_PHOSPHORUS_CONTAINING_GROUPS	233	0.38622758	1.3428963	0.15587045	0.23687142
GO_ENDOPLASMIC_RETICULUM_SUBCOMPARTMENT	16	0.5017041	1.3425262	0.13778706	0.23611408
GO_ENDORIBONUCLEASE_COMPLEX	17	0.51173073	1.3410311	0.15800416	0.23668586
GO_PROTEIN_KINASE_COMPLEX	89	0.3974052	1.3408469	0.13292433	0.23562504
GO_GEMINI_OF_COILED_BODIES	10	0.5861722	1.3382926	0.1680498	0.23715729
GO_EXON_EXON_JUNCTION_COMPLEX	21	0.52045196	1.3318206	0.14910537	0.24316098
GO_NUCLEAR_ENVELOPE	400	0.34301764	1.3304824	0.14663951	0.24342686
GO_CLATHRIN_VESICLE_COAT	23	0.45778513	1.3296793	0.138833	0.24322869
GO_OUTER_MITOCHONDRIAL_MEMBRANE_PROTEIN_COMPLEX	12	0.5612569	1.3252932	0.16221766	0.24678905
GO_SUPRAMOLECULAR_FIBER	646	0.3024545	1.3243201	0.081196584	0.24669227
GO_CENTRIOLAR_SATELLITE	24	0.45929345	1.3214265	0.12830958	0.24896678
GO_TRANSCRIPTIONALLY_ACTIVE_CHROMATIN	19	0.4687087	1.3193872	0.15062761	0.24999443
**Molecular function (57 terms)**					
GO_CYCLIN_DEPENDENT_PROTEIN_KINASE_ACTIVITY	34	0.59596914	1.772709	0.002004008	0.2264464
GO_DISULFIDE_OXIDOREDUCTASE_ACTIVITY	31	0.5639808	1.7726935	0.004	0.20380178
GO_UNFOLDED_PROTEIN_BINDING	93	0.5652977	1.7709087	0.013861386	0.1883973
GO_DNA_SECONDARY_STRUCTURE_BINDING	24	0.6747988	1.7625717	0.005964215	0.18935034
GO_DEOXYRIBONUCLEASE_ACTIVITY	65	0.54601496	1.7546927	0.003838772	0.19224492
GO_FOUR_WAY_JUNCTION_DNA_BINDING	15	0.73733616	1.7443064	0.003992016	0.19441648
GO_TELOMERASE_RNA_BINDING	13	0.73477966	1.7424701	0.005780347	0.18500839
GO_DNA_APURINIC_OR_APYRIMIDINIC_SITE_LYASE_ACTIVITY	14	0.67090636	1.7395418	0.005736138	0.1779046
GO_TRANSLATION_FACTOR_ACTIVITY_RNA_BINDING	85	0.5673898	1.7317507	0.01417004	0.18196087
GO_SNORNA_BINDING	26	0.66639566	1.7308675	0.0227704	0.17317854
GO_ENDODEOXYRIBONUCLEASE_ACTIVITY	49	0.5563542	1.730388	0.003875969	0.16459635
GO_RNA_POLYMERASE_ACTIVITY	43	0.5890996	1.7267307	0.009633912	0.16133244
GO_EXODEOXYRIBONUCLEASE_ACTIVITY	15	0.6851024	1.724979	0.007633588	0.15597253
GO_DNA_POLYMERASE_BINDING	12	0.7362106	1.7079195	0.011952192	0.17373806
GO_RNA_DNA_HYBRID_RIBONUCLEASE_ACTIVITY	7	0.85012937	1.702509	0.007797271	0.1755684
GO_POLY_A_RNA_BINDING	1136	0.5163767	1.7017269	0.006085193	0.16976267
GO_EXONUCLEASE_ACTIVITY_ACTIVE_WITH_EITHER_RIBO_OR_DEOXYRIBONUCLEIC_ACIDS_AND_PRODUCING_5_PHOSPHOMONOESTERS	42	0.6049815	1.7005074	0.003921569	0.16501117
GO_OXIDOREDUCTASE_ACTIVITY_OXIDIZING_METAL_IONS	18	0.59686893	1.6979871	0.015968064	0.16195983
GO_RIBONUCLEOPROTEIN_COMPLEX_BINDING	94	0.5515675	1.6961001	0.01004016	0.15839937
GO_DNA_HELICASE_ACTIVITY	52	0.6337112	1.6949878	0.011904762	0.15478322
GO_ATP_DEPENDENT_MICROTUBULE_MOTOR_ACTIVITY	18	0.620893	1.6936761	0.002079002	0.15116644
GO_LIGASE_ACTIVITY_FORMING_CARBON_OXYGEN_BONDS	44	0.61798465	1.6883807	0.020283977	0.15364894
GO_NUCLEOTIDYLTRANSFERASE_ACTIVITY	123	0.5028087	1.67752	0.0078125	0.16441652
GO_HISTONE_KINASE_ACTIVITY	19	0.645991	1.6714854	0.02631579	0.16863915
GO_RNA_BINDING	1538	0.4852454	1.6673499	0.014112903	0.17003563

GO_AMINOACYL_TRNA_EDITING_ACTIVITY	9	0.7373682	1.6592922	0.01417004	0.17785034
GO_PHOSPHATASE_ACTIVATOR_ACTIVITY	11	0.6918341	1.6559213	0.013182675	0.17761366
GO_L_ASCORBIC_ACID_BINDING	17	0.6026333	1.6534616	0.014403292	0.17594169
GO_MACROLIDE_BINDING	18	0.568697	1.6533175	0.033663366	0.17139137
GO_INTRAMOLECULAR_TRANSFERASE_ACTIVITY	27	0.57158434	1.6518219	0.012219959	0.16927741
GO_RNA_POLYMERASE_II_CARBOXY_TERMINAL_DOMAIN_KINASE_ACTIVITY	16	0.6771608	1.6509368	0.005964215	0.16651683
GO_STRUCTURE_SPECIFIC_DNA_BINDING	118	0.4839008	1.6368496	0.010245902	0.18350117
GO_OXIDOREDUCTASE_ACTIVITY_ACTING_ON_A_SULFUR_GROUP_OF_DONORS	48	0.48290274	1.6363481	0.018255578	0.1798862
GO_ATP_DEPENDENT_DNA_HELICASE_ACTIVITY	33	0.62332255	1.6339344	0.01192843	0.17932701
GO_UBIQUITIN_LIKE_PROTEIN_CONJUGATING_ENZYME_ACTIVITY	29	0.59377337	1.6145074	0.025145067	0.20471442
GO_ENDODEOXYRIBONUCLEASE_ACTIVITY_PRODUCING_5_PHOSPHOMONOESTERS	11	0.63884395	1.6143475	0.021653544	0.20025939
GO_METAL_CLUSTER_BINDING	61	0.48897392	1.6123128	0.01968504	0.19880192
GO_CIS_TRANS_ISOMERASE_ACTIVITY	45	0.49063978	1.6090858	0.024096385	0.20019035
GO_ISOMERASE_ACTIVITY	156	0.40923655	1.6086198	0.012345679	0.19650543
GO_STRUCTURAL_CONSTITUENT_OF_NUCLEAR_PORE	13	0.7319034	1.5983399	0.010183299	0.20838094
GO_NUCLEOSIDE_KINASE_ACTIVITY	13	0.62519777	1.5966307	0.019157087	0.20682418
GO_NF_KAPPAB_BINDING	30	0.5529674	1.5868734	0.031936128	0.21856599
GO_RIBOSOMAL_SMALL_SUBUNIT_BINDING	12	0.6759848	1.5858672	0.024742268	0.2159485
GO_EXONUCLEASE_ACTIVITY	73	0.51389986	1.5818905	0.028846154	0.21884224
GO_ENDONUCLEASE_ACTIVITY_ACTIVE_WITH_EITHER_RIBO_OR_DEOXYRIBONUCLEIC_ACIDS_AND_PRODUCING_5_PHOSPHOMONOESTERS	34	0.5370378	1.5810101	0.035019454	0.2161074
GO_3_5_EXONUCLEASE_ACTIVITY	47	0.5491874	1.570972	0.031558186	0.23034796
GO_STRUCTURAL_CONSTITUENT_OF_RIBOSOME	202	0.5533821	1.5599234	0.10515873	0.24592353
GO_METHYL_CPG_BINDING	20	0.573909	1.5590467	0.03041825	0.24335279
GO_INTRAMOLECULAR_OXIDOREDUCTASE_ACTIVITY_INTERCONVERTING_ALDOSES_AND_KETOSES	11	0.6543069	1.5538702	0.021912351	0.24854536
**C6 Oncological signature (28 terms)**					
BIOCARTA_PTC1_PATHWAY	11	0.8459149	1.945383	0.002118644	0.06398674
BIOCARTA_P27_PATHWAY	13	0.7969798	1.9289968	0	0.038441528
BIOCARTA_G2_PATHWAY	24	0.6917375	1.9036189	0.006160164	0.03788111
BIOCARTA_CELLCYCLE_PATHWAY	23	0.65959394	1.8606957	0.00408998	0.045502532
BIOCARTA_PROTEASOME_PATHWAY	28	0.77732474	1.8319854	0.007936508	0.04707304
BIOCARTA_GLYCOLYSIS_PATHWAY	10	0.79025996	1.7912003	0.010351967	0.06358903
BIOCARTA_MCM_PATHWAY	18	0.71597147	1.7694954	0.018556701	0.06876878
BIOCARTA_HIF_PATHWAY	15	0.6840519	1.7625527	0.004366812	0.065344326
BIOCARTA_SRCRPTP_PATHWAY	11	0.71615475	1.7487165	0.010615711	0.06870219
BIOCARTA_RB_PATHWAY	13	0.6846301	1.7439233	0	0.06439214
BIOCARTA_RANMS_PATHWAY	10	0.82737935	1.7220055	0.016	0.073742755
BIOCARTA_SKP2E2F_PATHWAY	10	0.702789	1.7103677	0.005928854	0.075584196
BIOCARTA_SALMONELLA_PATHWAY	13	0.7176766	1.7035477	0.007968128	0.07478445
BIOCARTA_EIF2_PATHWAY	11	0.76882476	1.7004058	0.002024292	0.07195907
BIOCARTA_RAB_PATHWAY	12	0.70288587	1.6705955	0.021868788	0.08710322
BIOCARTA_EIF_PATHWAY	16	0.6816011	1.6425833	0.021868788	0.10431794
BIOCARTA_ATRBRCA_PATHWAY	21	0.63465095	1.6402888	0.017716536	0.10076495
BIOCARTA_RACCYCD_PATHWAY	26	0.5962543	1.6380337	0.02008032	0.096681274

BIOCARTA_FREE_PATHWAY	9	0.6763291	1.5989605	0.023861172	0.12729096
BIOCARTA_UCALPAIN_PATHWAY	18	0.60840744	1.580332	0.031578947	0.14142391
BIOCARTA_CDC42RAC_PATHWAY	16	0.65308535	1.5594262	0.033864543	0.15784384
BIOCARTA_G1_PATHWAY	28	0.5214182	1.5509028	0.046938777	0.1612215
BIOCARTA_MTA3_PATHWAY	18	0.538474	1.5497025	0.039130434	0.15579833
BIOCARTA_P35ALZHEIMERS_PATHWAY	11	0.60613376	1.4991324	0.03976143	0.21654968
BIOCARTA_DNAFRAGMENT_PATHWAY	10	0.628387	1.4850376	0.072434604	0.22850932
BIOCARTA_ATM_PATHWAY	20	0.5571135	1.4751178	0.064449064	0.23431115
BIOCARTA_ACTINY_PATHWAY	20	0.54589474	1.4745562	0.07185629	0.22656365
BIOCARTA_PTDINS_PATHWAY	23	0.5363502	1.45985	0.071428575	0.23828006
**Terms enriched in low risk group**					
**C2 KEGG (1 terms)**					
KEGG_NEUROACTIVE_LIGAND_RECEPTOR_INTERACTION	270	-0.5301249	-1.9103235	0	0.09315833
**C5**					
**Biological process (0 terms)**					
**Cellular component (3 terms)**					
GO_MAIN_AXON	57	-0.6137751	-1.9344873	0	0.13338119
GO_APICAL_DENDRITE	13	-0.68443125	-1.8339114	0.001848429	0.20765187
GO_NEURON_PROJECTION_MEMBRANE	34	-0.5705935	-1.6494124	0.012433393	0.24696921
**Molecular function (30 terms)**					
GO_POTASSIUM_CHANNEL_ACTIVITY	119	-0.5401616	-1.881483	0	0.18842526
GO_SIGNALING_RECEPTOR_ACTIVITY	1321	-0.40520954	-1.8378897	0	0.22552337
GO_G_PROTEIN_COUPLED_RECEPTOR_ACTIVITY	817	-0.38562855	-1.8358582	0	0.17340446
GO_PEPTIDE_RECEPTOR_ACTIVITY	127	-0.54304916	-1.8121959	0.003584229	0.18765643
GO_DELAYED_RECTIFIER_POTASSIUM_CHANNEL_ACTIVITY	36	-0.6197212	-1.8067665	0.003478261	0.16693386
GO_PHOSPHORIC_DIESTER_HYDROLASE_ACTIVITY	90	-0.4992297	-1.7794026	0	0.19396083
GO_RECEPTOR_ACTIVITY	1563	-0.39239866	-1.777063	0.001838235	0.17344797
GO_ADRENERGIC_RECEPTOR_BINDING	19	-0.6341564	-1.7739949	0.006036217	0.15868044
GO_CARBOXYPEPTIDASE_ACTIVITY	38	-0.53562874	-1.7690663	0.001703578	0.15114729
GO_VOLTAGE_GATED_CATION_CHANNEL_ACTIVITY	133	-0.49740162	-1.7464465	0.004926108	0.17381446
GO_SERINE_TYPE_EXOPEPTIDASE_ACTIVITY	18	-0.6049076	-1.7460067	0.003745318	0.16047662
GO_GATED_CHANNEL_ACTIVITY	319	-0.454151	-1.7320489	0.001652893	0.1702009
GO_CATION_CHANNEL_ACTIVITY	290	-0.4486865	-1.7271901	0.00331675	0.16735497
GO_LIGAND_GATED_CHANNEL_ACTIVITY	138	-0.46703282	-1.7210503	0.001636661	0.16623475
GO_G_PROTEIN_COUPLED_CHEMOATTRACTANT_RECEPTOR_ACTIVITY	22	-0.7278793	-1.7166375	0.020183487	0.16316581
GO_CYCLIC_NUCLEOTIDE_PHOSPHODIESTERASE_ACTIVITY	25	-0.6101103	-1.7129616	0.005736138	0.15961972
GO_PHOSPHOLIPASE_C_ACTIVITY	31	-0.5476513	-1.7128425	0.007518797	0.15093626
GO_EXTRACELLULAR_LIGAND_GATED_ION_CHANNEL_ACTIVITY	75	-0.51395744	-1.7126123	0.006568145	0.14321831
GO_3_5_CYCLIC_GMP_PHOSPHODIESTERASE_ACTIVITY	14	-0.67524856	-1.7057961	0.015296367	0.145294
GO_VOLTAGE_GATED_SODIUM_CHANNEL_ACTIVITY	20	-0.65179735	-1.6942664	0.014159292	0.15501155
GO_VOLTAGE_GATED_ION_CHANNEL_ACTIVITY	188	-0.4572963	-1.677874	0.006644518	0.17324595
GO_CYTOKINE_RECEPTOR_ACTIVITY	85	-0.5309697	-1.6516032	0.030797102	0.21209529
GO_PEPTIDE_HORMONE_BINDING	36	-0.5490085	-1.645897	0.009345794	0.2134605
GO_CYCLASE_ACTIVITY	21	-0.57964176	-1.644735	0.018656716	0.20693746

GO_3_5_CYCLIC_AMP_PHOSPHODIESTERASE_ACTIVITY	15	-0.6743193	-1.6427832	0.0227704	0.20302416
GO_INWARD_RECTIFIER_POTASSIUM_CHANNEL_ACTIVITY	22	-0.5571651	-1.6124709	0.021558871	0.24808282
GO_SIGNAL_TRANSDUCER_ACTIVITY	1652	-0.34759322	-1.5912715	0.007590133	0.24472035
GO_SERINE_TYPE_CARBOXYPEPTIDASE_ACTIVITY	14	-0.5939026	-1.5881804	0.03564728	0.24348828
GO_IMMUNOGLOBULIN_RECEPTOR_BINDING	7	-0.8023157	-1.5816555	0.020522388	0.24862528
GO_CAMP_BINDING	22	-0.553671	-1.5807339	0.021778584	0.24358347
**C6 Oncological signature (terms)**					
BIOCARTA_GRANULOCYTES_PATHWAY	13	-0.8286528	-1.8326796	0.001858736	0.14008604
BIOCARTA_THELPER_PATHWAY	12	-0.8545251	-1.545204	0.029126214	0.23535249
BIOCARTA_TCYTOTOXIC_PATHWAY	12	-0.8540751	-1.5399984	0.034951456	0.23010892

Supplementary figure legends

Figure S1 LASSO analysis of the 36 prognostic DE-MTGs in PDAC. (A) LASSO coefficient profiles of the 36 prognostic DE-MTGs in PDAC. (B) Lasso deviance profiles of the 36 prognostic DE-MTGs in PDAC.

Figure S2 (A–C) ROC curves for 1-, 2-, and 3-year overall survival predictions for the MTG-based gene signature compared with those of the AJCC staging system and three previously defined gene signatures in the TCGA-PAAD datasets. (D–F) ROC curves for 1-, 2-, and 3-year overall survival predictions for the MTG-based gene signature compared with those of the AJCC staging system and three previously defined gene signatures in the external validation dataset GSE62452. (G–I) ROC curves for 1-, 2-, and 3-year overall survival predictions for the MTG-based gene signature compared with those of the three previously defined gene signatures in the external validation dataset ICGC.

Figure S3 (A–C) ROC curves for 1-, 2-, and 3-year overall survival predictions of the nomogram compared with those of the AJCC staging system in TCGA-PAAD dataset. (D–F) ROC curves for 1-, 2-, and 3-year overall survival predictions of the nomogram compared with those of the gene signature proposed by Zhou C et al. in TCGA-PAAD dataset. (G–I) ROC curves for 1-, 2-, and 3-year overall survival predictions of the nomogram compared with those of the gene signature proposed by Chen H et al. in TCGA-PAAD dataset. (J–L) ROC curves for 1-, 2-, and 3-year overall survival predictions of the nomogram compared with those of the gene signature proposed by Liao X et al. in TCGA-PAAD dataset.

Figure S1

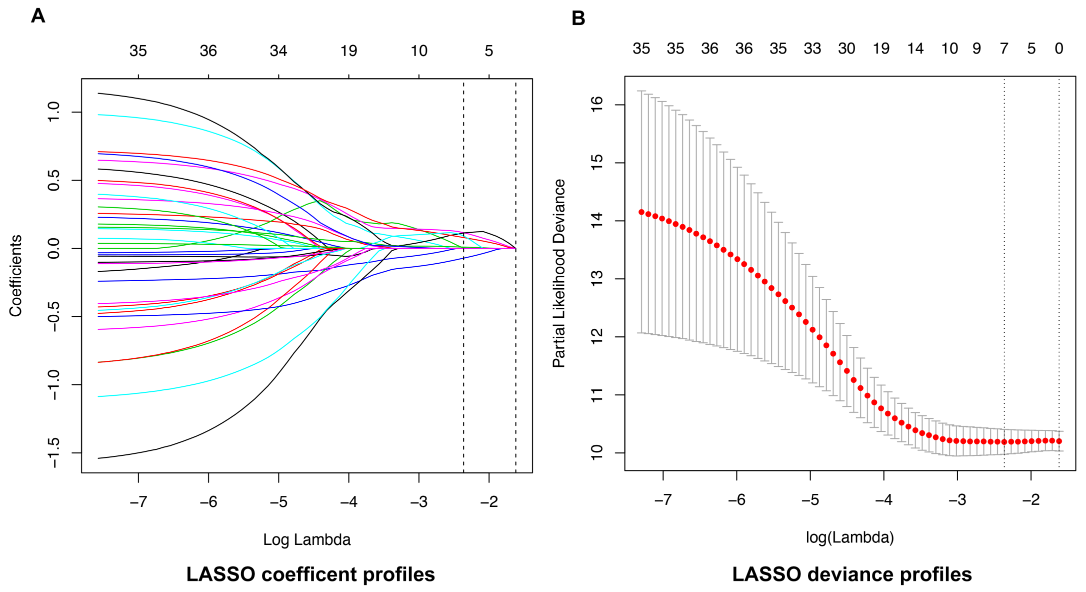
A
35 36 34 19 10 5
Coefficients
Log Lambda
LASSO coefficent profiles
B
35 35 36 36 35 33 30 19 14 10 9 7 5 0
Partial Likelihood Deviance
log(Lambda)
LASSO deviance profiles

Figure S2

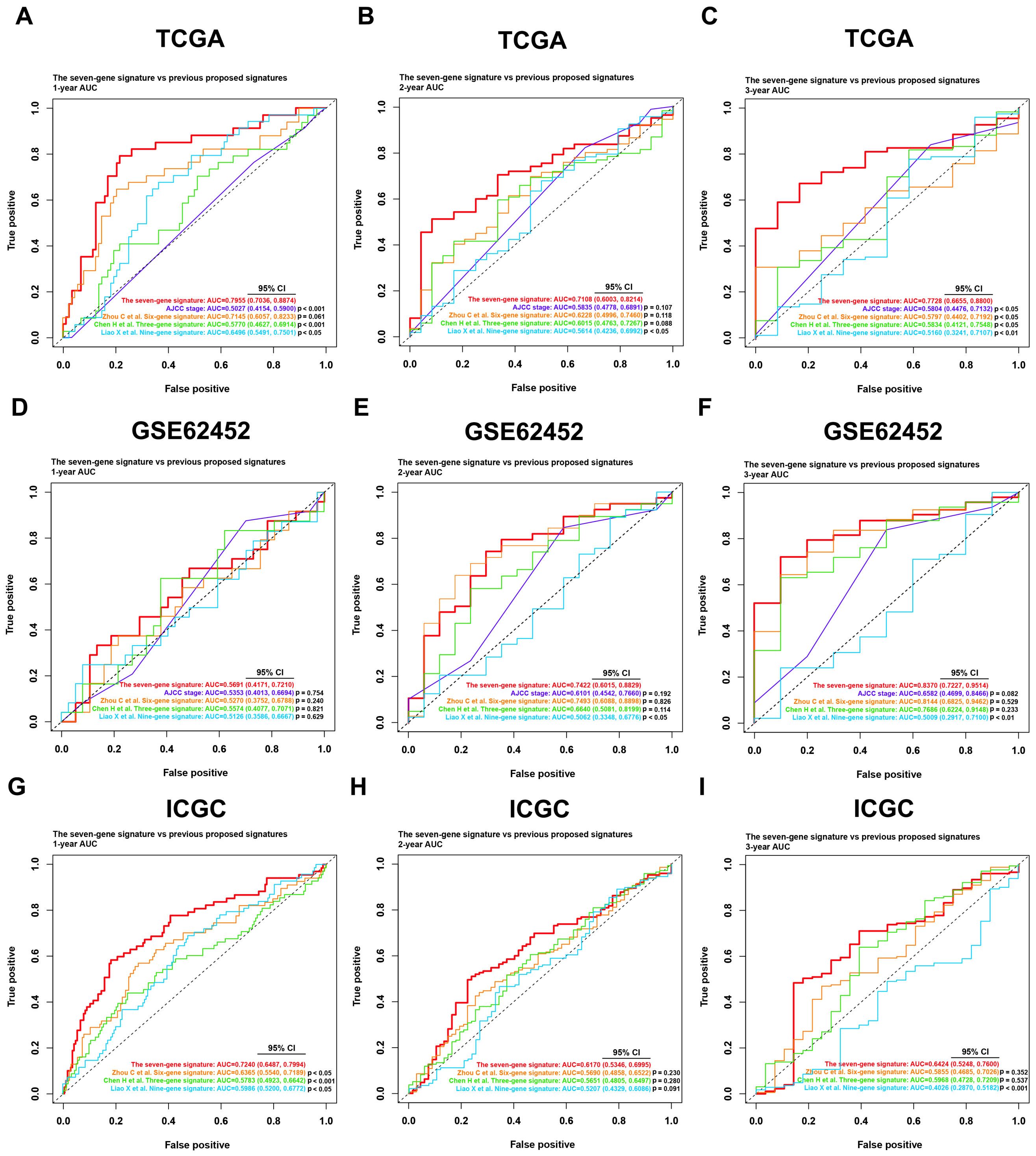
A
TCGA
The seven-gene signature vs previous proposed signatures
1-year AUC
95% CI
The seven-gene signature: AUC=0.7955 (0.7036, 0.8874)
AJCC stage: AUC=0.5027 (0.4154, 0.5900) p < 0.001
Zhou C et al. Six-gene signature: AUC=0.7145 (0.6057, 0.8233) p = 0.061
Chen H et al. Three-gene signature: AUC=0.5770 (0.4627, 0.6914) p < 0.001
Liao X et al. Nine-gene signature: AUC=0.6496 (0.5491, 0.7501) p < 0.05
True positive
False positive
B
TCGA
The seven-gene signature vs previous proposed signatures
2-year AUC
95% CI
The seven-gene signature: AUC=0.7108 (0.6003, 0.8214)
AJCC stage: AUC=0.5835 (0.4778, 0.6891) p = 0.107
Zhou C et al. Six-gene signature: AUC=0.6228 (0.4996, 0.7460) p = 0.118
Chen H et al. Three-gene signature: AUC=0.6015 (0.4763, 0.7267) p = 0.088
Liao X et al. Nine-gene signature: AUC=0.5614 (0.4236, 0.6992) p < 0.05
C
TCGA
The seven-gene signature vs previous proposed signatures
3-year AUC
95% CI
The seven-gene signature: AUC=0.7728 (0.6655, 0.8800)
AJCC stage: AUC=0.5804 (0.4476, 0.7132) p < 0.05
Zhou C et al. Six-gene signature: AUC=0.5797 (0.4402, 0.7192) p < 0.05
Chen H et al. Three-gene signature: AUC=0.5834 (0.4121, 0.7548) p < 0.05
Liao X et al. Nine-gene signature: AUC=0.5160 (0.3241, 0.7107) p < 0.01
D
GSE62452
The seven-gene signature vs previous proposed signatures
1-year AUC
95% CI
The seven-gene signature: AUC=0.5691 (0.4171, 0.7210)
AJCC stage: AUC=0.5353 (0.4013, 0.6694) p = 0.754
Zhou C et al. Six-gene signature: AUC=0.5270 (0.3752, 0.6788) p = 0.240
Chen H et al. Three-gene signature: AUC=0.5574 (0.4077, 0.7071) p = 0.821
Liao X et al. Nine-gene signature: AUC=0.5126 (0.3586, 0.6667) p = 0.629
E
GSE62452
The seven-gene signature vs previous proposed signatures
2-year AUC
95% CI
The seven-gene signature: AUC=0.7422 (0.6015, 0.8829)
AJCC stage: AUC=0.6101 (0.4542, 0.7660) p = 0.192
Zhou C et al. Six-gene signature: AUC=0.7493 (0.6088, 0.8898) p = 0.826
Chen H et al. Three-gene signature: AUC=0.6640 (0.5081, 0.8199) p = 0.114
Liao X et al. Nine-gene signature: AUC=0.5062 (0.3348, 0.6776) p < 0.05
F
GSE62452
The seven-gene signature vs previous proposed signatures
3-year AUC
95% CI
The seven-gene signature: AUC=0.8370 (0.7227, 0.9514)
AJCC stage: AUC=0.6582 (0.4699, 0.8466) p = 0.082
Zhou C et al. Six-gene signature: AUC=0.8144 (0.6825, 0.9462) p = 0.529
Chen H et al. Three-gene signature: AUC=0.7686 (0.6224, 0.9148) p = 0.233
Liao X et al. Nine-gene signature: AUC=0.5009 (0.2917, 0.7100) p < 0.01
G
ICGC
The seven-gene signature vs previous proposed signatures
1-year AUC
95% CI
The seven-gene signature: AUC=0.7240 (0.6487, 0.7994)
Zhou C et al. Six-gene signature: AUC=0.6365 (0.5540, 0.7189) p < 0.05
Chen H et al. Three-gene signature: AUC=0.5783 (0.4923, 0.6642) p < 0.001
Liao X et al. Nine-gene signature: AUC=0.5986 (0.5200, 0.6772) p < 0.05
H
ICGC
The seven-gene signature vs previous proposed signatures
2-year AUC
95% CI
The seven-gene signature: AUC=0.6170 (0.5346, 0.6995)
Zhou C et al. Six-gene signature: AUC=0.5690 (0.4858, 0.6522) p = 0.230
Chen H et al. Three-gene signature: AUC=0.5651 (0.4805, 0.6497) p = 0.280
Liao X et al. Nine-gene signature: AUC=0.5207 (0.4329, 0.6086) p = 0.091
I
ICGC
The seven-gene signature vs previous proposed signatures
3-year AUC
95% CI
The seven-gene signature: AUC=0.6424 (0.5248, 0.7600)
Zhou C et al. Six-gene signature: AUC=0.5855 (0.4685, 0.7026) p = 0.352
Chen H et al. Three-gene signature: AUC=0.5968 (0.4728, 0.7209) p = 0.537
Liao X et al. Nine-gene signature: AUC=0.4026 (0.2870, 0.5182) p < 0.001

Figure S3

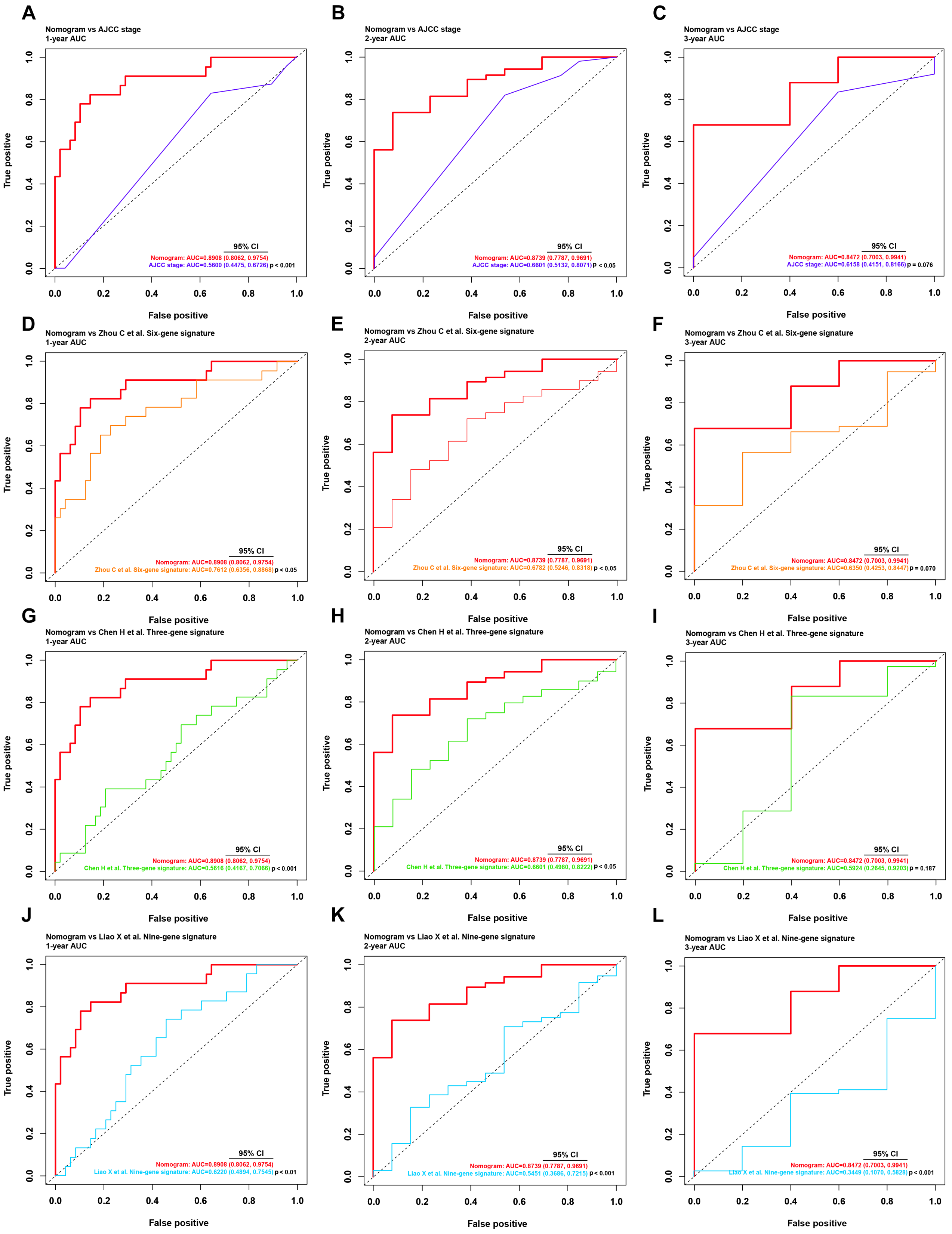
A
Nomogram vs AJCC stage
1-year AUC
Nomogram: AUC=0.8908 (0.8062, 0.9754)
AJCC stage: AUC=0.5600 (0.4475, 0.6726) p < 0.001
B
Nomogram vs AJCC stage
2-year AUC
Nomogram: AUC=0.8739 (0.7787, 0.9691)
AJCC stage: AUC=0.6601 (0.5132, 0.8071) p < 0.05
C
Nomogram vs AJCC stage
3-year AUC
Nomogram: AUC=0.8472 (0.7003, 0.9941)
AJCC stage: AUC=0.6158 (0.4151, 0.8166) p = 0.076
D
Nomogram vs Zhou C et al. Six-gene signature
1-year AUC
Nomogram: AUC=0.8908 (0.8062, 0.9754)
Zhou C et al. Six-gene signature: AUC=0.7612 (0.6356, 0.8868) p < 0.05
E
Nomogram vs Zhou C et al. Six-gene signature
2-year AUC
Nomogram: AUC=0.8739 (0.7787, 0.9691)
Zhou C et al. Six-gene signature: AUC=0.6782 (0.5246, 0.8318) p < 0.05
F
Nomogram vs Zhou C et al. Six-gene signature
3-year AUC
Nomogram: AUC=0.8472 (0.7003, 0.9941)
Zhou C et al. Six-gene signature: AUC=0.6350 (0.4253, 0.8447) p = 0.070
G
Nomogram vs Chen H et al. Three-gene signature
1-year AUC
Nomogram: AUC=0.8908 (0.8062, 0.9754)
Chen H et al. Three-gene signature: AUC=0.5616 (0.4167, 0.7066) p < 0.001
H
Nomogram vs Chen H et al. Three-gene signature
2-year AUC
Nomogram: AUC=0.8739 (0.7787, 0.9691)
Chen H et al. Three-gene signature: AUC=0.6601 (0.4980, 0.8222) p < 0.05
I
Nomogram vs Chen H et al. Three-gene signature
3-year AUC
Nomogram: AUC=0.8472 (0.7003, 0.9941)
Chen H et al. Three-gene signature: AUC=0.5924 (0.2645, 0.9203) p = 0.187
J
Nomogram vs Liao X et al. Nine-gene signature
1-year AUC
Nomogram: AUC=0.8908 (0.8062, 0.9754)
Liao X et al. Nine-gene signature: AUC=0.6220 (0.4894, 0.7545) p < 0.01
K
Nomogram vs Liao X et al. Nine-gene signature
2-year AUC
Nomogram: AUC=0.8739 (0.7787, 0.9691)
Liao X et al. Nine-gene signature: AUC=0.5451 (0.3686, 0.7215) p < 0.001
L
Nomogram vs Liao X et al. Nine-gene signature
3-year AUC
Nomogram: AUC=0.8472 (0.7003, 0.9941)
Liao X et al. Nine-gene signature: AUC=0.3449 (0.1070, 0.5828) p < 0.001
95% CI
True positive
False positive
